# Links between fish abundance and ocean biogeochemistry as recorded in marine sediments

**DOI:** 10.1371/journal.pone.0199420

**Published:** 2018-08-01

**Authors:** Lucas Kavanagh, Eric Galbraith

**Affiliations:** 1 Department of Earth and Planetary Sciences, McGill University, Montréal, Québec, Canada; 2 ICREA, Pg. Lluís Companys 23, Barcelona, Spain; 3 Institut de Ciencia i Tecnologia Ambientals (ICTA), Universitat Autònoma de Barcelona, Spain; Technical University of Denmark, DENMARK

## Abstract

Fish populations are linked to ocean biogeochemistry by their reliance on primary production for food, and dissolved oxygen to breathe. It is also possible that marine fish modify biogeochemical dynamics, as do freshwater fish, through top-down trophic cascades, but there has been relatively little consideration of this possibility. This lack of consideration may reflect a lack of importance; alternatively, it may simply reflect the lack of appropriate observations with which to constrain such relationships. Here, we draw attention to the potential use of marine sediments as long-term simultaneous monitors of both fish abundance and marine biogeochemical dynamics. We compile published sediment proxy records of fish abundance from the west coasts of the Americas, and compare them with biogeochemical proxy measurements made at the same sites. Despite the challenges of using sediment records and the potential convolution of ecological and climatic signals, we find a small number of statistically significant relationships between fish debris and biogeochemical variables, at least some of which are likely to reflect causal relationships. Considering TOC, the most commonly-measured biogeochemical variable, some positive correlations with fish abundance are found, consistent with bottom-up control of fish abundance by primary production, or a planktivore-herbivore-phytoplankton trophic cascade. Negative correlations are also found, which could reflect sedimentary processes, the influence of upwelling-driven oxygen and nutrient dynamics on primary production and fish populations, and/or impacts of fish stocks on carbon fluxes by altering the recycling of carbon within the water column. Although the number of available measurements is too small to draw strong conclusions, the results point to plausible cases of bottom-up forcing, trophic cascades, and influence of dissolved oxygen concentrations on fish habitat.

## Introduction

Marine fish are inextricably linked to biogeochemical cycles through their reliance on photosynthetically-captured energy and dissolved oxygen. Phytoplankton transform dissolved inorganic carbon into organic matter, at a rate that depends on nutrient limitation, water temperature, light exposure, and grazing control of phytoplankton biomass [1]. The organic matter is then transferred to fish via heterotrophic pathways, so that the abundance of fish depends to some degree on photosynthesis [2]. Dissolved oxygen also exerts a critical environmental influence on fish, given that fish communities are metabolically constrained by the combination of water temperature and oxygen concentrations [3].

It is typically assumed that the reverse dependency, of ocean biogeochemistry on fish, is negligible in the ocean due to the relatively slow metabolic rates of fish. However, fish have been shown to significantly affect biogeochemical cycling in lakes through trophic cascades [4], and it has previously been argued that similar processes may occur to a lesser, but perhaps still significant degree in the ocean [5–10]. These changes could be manifested as changes in the export fraction (i.e. the f-ratio) and transfer efficiency of sinking particles [11, 12], with consequences for the biological ‘soft tissue’ carbon pump and oxygen consumption in the deep ocean. Thus, it remains an open question whether the abundance of fish, and their impact on the structure of marine communities, alters the cycling of nutrients, carbon and oxygen in the ocean in a consequential way. This question has implications for the long-term evolution of ocean ecology [13, 14], and gains a sense of urgency in the context of industrial fishing, which may have depleted the biomass of large predatory fish by 90% since preindustrial times [15]; if fish do influence biogeochemical cycling to a significant extent, their removal may have already had unappreciated consequences for ocean biogeochemistry.

Bottom-up control, widely believed to dominate in the ocean [16] implies that environmentally-driven changes in primary productivity control the abundance of ‘higher’ trophic levels (by which we mean all organisms above zooplankton), by modifying the supply of food. As such, all trophic levels would be expected to flourish and decline together. Top-down control, on the other hand, implies that the biomass structure within a population is significantly altered by predation, so that the abundance of predators and their prey would vary inversely over time [6, 17, 18]. Top-down control provides a potential mechanism for fish to impact biogeochemistry, despite their relatively slow metabolic rates, by controlling the abundance of the trophic levels below them. Top-down control is widely recognized in fresh water ecosystems as ‘trophic cascades’, whereby the reduction of biomass at one trophic level releases their prey from predatory pressure, causing them to increase in abundance. The next trophic level down would be depleted, in turn. This pattern of depleting the biomass of alternate trophic levels was first observed in freshwater fish removal experiments [4, 19] and was shown to have profound biogeochemical impacts. Trophic cascades induced by the addition or removal of fish from lakes can abruptly increase phytoplankton biomass, which lowers water column transparency, reduces the depth of light penetration and causes the thermal stratification and mixing depth to shoal [20]. The increased thermal stratification, combined with a greater amount of slow-sinking phytodetritus can increase sub-surface respiration and significantly deplete hypolimnetic oxygen concentrations [21].

The simple trophic cascade mechanism documented in lakes is not directly transferable to marine ecosystems due to their higher diversity, fewer discrete trophic levels, and the relative ease with which organisms can migrate and/or be transported by ocean currents over long distances [10]. However, there are indications that some forms of trophic cascades can occur in the marine environment [22]. Top-down control in North Atlantic ecosystems has been suggested by observed negative correlations in timeseries of predator-prey abundances [17]. Trophic cascades have been reported as a result of human activities in the Peruvian Upwelling [5], Baltic Sea [8], Black Sea [9], Scotian Shelf [7] and Namibian upwelling [23]. All of these putative trophic cascades are related to intensive industrial fishing, and appear to correspond to observed and documented biogeochemical changes: an increase in chlorophyll *a* concentrations in the Baltic Sea, oxygen depletion in the Black Sea, an increase in water column nitrogen on the Scotian Shelf, and an increase in organic matter flux to sediments in the Peruvian and Namibian upwellings. These observations have been used to suggest that upper trophic levels can exert a significant influence on the marine biogeochemical environment, at least in certain environments and ecosystems. ‘Wasp-waist’ ecosystems, in which a relatively small number of forage fish with high turnover rates acts as a critical intermediary between lower and upper trophic levels, have been identified as particularly prone to trophic instability [24].

The consumption of herbivorous zooplankton by planktivorous forage fish can alter the abundance and species composition of phytoplankton, as observed in the Scotian Shelf trophic cascade [7]. In environments where nutrients or light are available to phytoplankton in excess (i.e. not limiting primary production), the consequent impacts on phytoplankton biomass may alter overall rates of primary production. In addition, control of the zooplankton population and community structure could modify the depth at which organic matter is respired, or ‘remineralized’ within the water column. Zooplankton fecal pellets account for a highly variable (between 0 and 100%) but often significant amount of carbon collected in sediment traps [25]. Zooplankton biomass, size, and community structure can have a significant influence on the remineralization profile of the water column and, thus, the export efficiency of particulate organic carbon [26]. This effect has been observed in fresh water systems where a shift towards smaller zooplankton results in less efficient grazing of phytoplankton and smaller, slower sinking particulate matter [21]. In addition, it is possible that the presence of predatory fish alters the behaviour of zooplankton [27], such as providing an incentive to undertake Diel Vertical Migration (DVM), which has been shown to have a significant impact on organic matter remineralization in the global thermocline [28].

In addition to controlling the biomass of zooplankton, fish can exert a direct control on the fluxes of organic matter and minerals. Fish create large fecal pellets that sink up to an order of magnitude faster than those of zooplankton [29]. Few observational studies distinguish fish pellets from zooplankton faeces, but anchovy fecal pellets have been found to account for up to 17% of organic carbon in sediment traps placed in the Peruvian Upwelling Zone [30]. Mesopelagic fish, particularly myctophids, also engage in DVM that facilitate the active transport of carbon out of the upper ocean by feeding near the surface at night and resting at depth during the day. It has been estimated that DVM by mesopelagic fish accounts for 15-17% of carbon export in the California Current [31]. The excretion of calcium carbonate in fish feces has also been argued to provide a surprisingly large vertical flux of carbonate [32].

**Fig 1:**
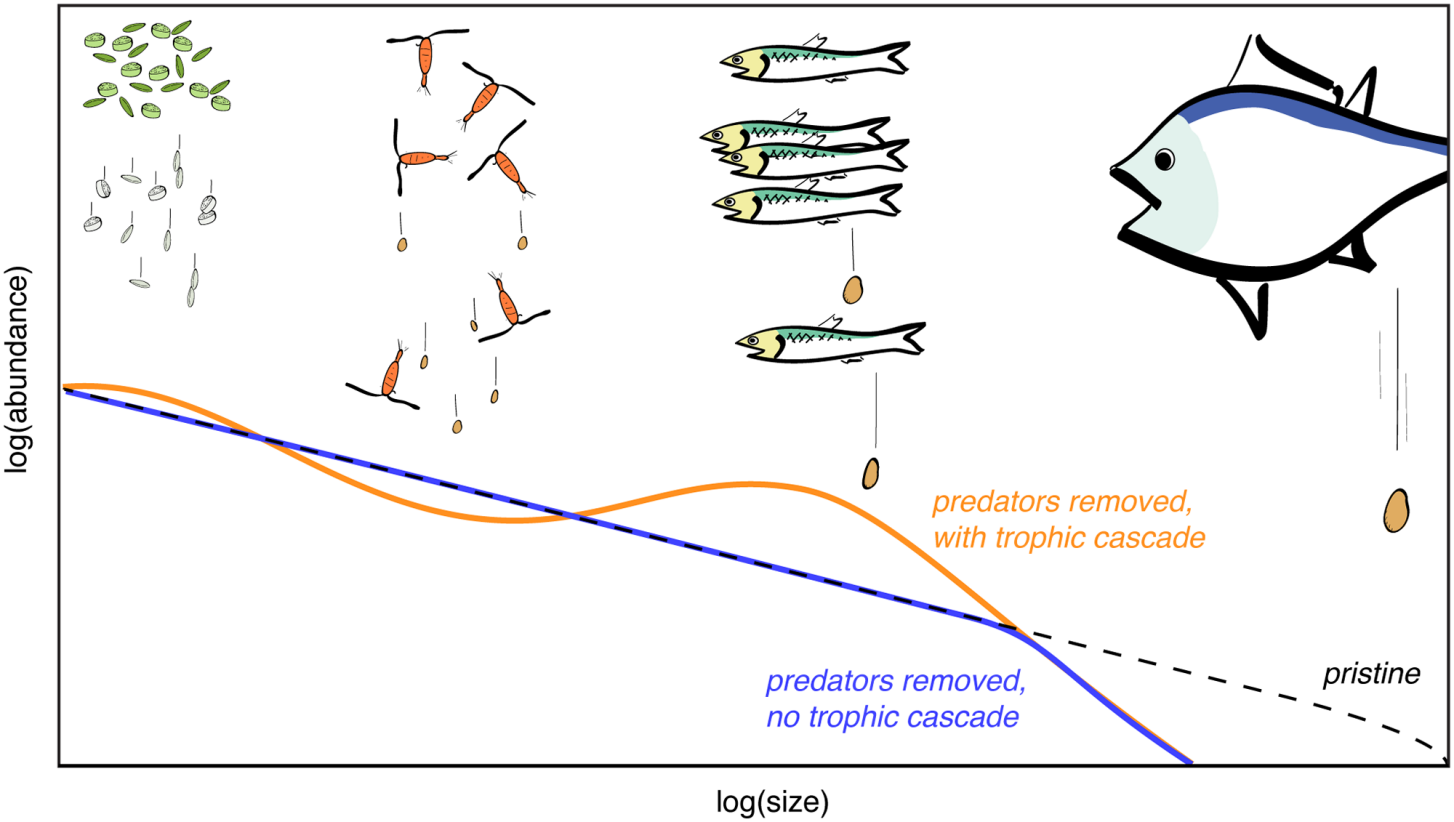
Schematic size spectra of marine communities and sources of sinking particles. The lines show idealized responses of community size spectra to changes in fish abundance. Because the abundance of organisms generally decreases with size, zooplankton will produce large amounts of more slowly-sinking faecal pellets, while large fish will produce small amounts of rapidly-sinking fecal pellets. The hypothetical predator-removal spectra are inspired by the simulated impacts of fishing on size spectrum models [18].

Fig 1 provides a graphical summary of possible interactions between fish abundance, the ecosystem size spectrum and sinking particle fluxes. Large fish may produce the fastest-sinking fecal pellets, but in relatively small quantities given their slower metabolic rates. As such, they can potentially exert a greater impact on particle cycling through top-down control of smaller organisms. For example, if piscivorous fish are removed from the system illustrated in Fig 1, and a trophic cascade occurs, the abundance of forage fish could be expected to increase, decreasing the abundance of zooplankton. If forage fish are removed from a system and a trophic cascade occurs, zooplankton would be expected to increase in abundance. In all cases, the effects on abundance are expected to be dampened at trophic levels further away from the initial perturbation [18].

Body size can also be considered as a first order indication of the trophic roles of different fish species [33]. For example, in eastern boundary upwelling systems, anchovy, sardine and hake are commonly found. The relative sizes of these fish, with anchovy and sardines being smaller than adult hake, generally corresponds to the relative sizes of their prey of plantkon vs. small fish [34, 35]. However there are additional differences between the species, such as the facts that hake is demersal (bottom-dwelling) whereas anchovies and sardines are pelagic, that sardines can filter phytoplankton from the water while anchovies feed by biting zooplankton, and that anchovy tend to outcompete sardines when water colummn oxygen concentrations are lower [36]. Thus, sensitivities to environmental changes will differ between species, as well as their potential to produce cascades or other biogeochemical effects.

Despite the numerous potential mechanisms by which fish might influence biogeochemistry, the provision of firm observational constraints is hampered by the spatio-temporal variability of ocean circulation and lower trophic level processes, the highly motile nature of fish, and confounding environmental changes [10]. Direct observations of individual fish can be scaled up to infer large-scale impacts on biogeochemistry [31, 32], but this is fraught with uncertainty, leaving time-series observations of past changes in fish abundance and biogeochemistry as a critical test [9, 17]. In order to be able to confidently identify trends, records of both fish abundance and biogeochemical parameters are needed, over a sufficiently long timescale to filter out the interference of other processes.

Here, we explore the possibility that impacts of fish on ocean biogeochemistry can be discerned in previously published proxy records from recent marine sediments. Marine sediment records have most often been collected with the aim of reconstructing past climate, but fish bones, otoliths or scales have been extracted and counted from a small number of them to reconstruct population abundance over time. Fish scales, composed of hydroxyapatite, are continually lost by most fish and can be preserved for thousands of years in anoxic sediments [37]. Fish scales were first counted in sediments of the Santa Barbara Basin [38] and later correlated to standing stock biomass in the same region [39] as well as more recently in Saanich Inlet [40]. Fish bones and otoliths are deposited by a different mechanism than scales, most commonly being excreted by predators [40]. Bones and otoliths are far more resistant to degradation than fish scales and thus will be less affected by varying preservation regimes [41, 42].

In addition to fish abundance proxies, common sediment measurements that might reveal relevant processes generally fall into three categories: climate state, primary and export production, and oxygenation state. Proxies that reflect climate include Sea Surface Temperature (SST) proxies, and lithogenic particles supplied by wind or runoff. Productivity-related quantities include Total Organic Carbon (TOC), calcium carbonate (CaCO_3_), opal, and species assemblages (e.g. diatoms, foraminifera, and dinoflagellate cysts). These proxies would be expected to reflect changes in primary productivity, while also depending on the export fraction (i.e. that which sinks to depth), which is controlled by ecosystem structure and the remineralization profile. Oxygenation proxies include trace metals that accumulate in sediments depending on the sediment oxidation state [43], sedimentary laminations, and benthic faunal assemblages. Oxygenation proxies may be used to test for relationships between fish stocks and oxygen concentrations within the water column, and may also help to detect changes in the preservation of organic matter. Bulk sedimentary *δ*^15^N reflects the cycling of nitrate, including sources and sinks of nitrogen as well as its utilization [44].

Despite its great potential, using and interpreting the sediment record presents considerable challenges (eg. [41]). Proxy signals are convolutions of climatic, oceanographic, sedimentary, and ecological influences, among which it can be difficult to differentiate. In addition, sedimentary records include a raft of uncertainties: chronologies of sediments are imperfectly known [45], sedimentary processes at the seafloor can leave imprints that have nothing to do with fish or biogeochemistry [46], and diagenetic alteration from the activity of heterotrophic organisms alters the composition of recently-deposited sediments [47]. What’s more, the amplitude of natural changes in overall fish abundance may have been smaller than those now underway due to fishing and climate change, and may therefore produce smaller biogeochemical impacts. It is entirely conceivable that any signals resulting from natural fluctuations in upper trophic level influence on biogeochemistry are simply too small to be preserved in sediments, or may be dwarfed by climatically-forced biogeochemical changes.

## Methods

**Fig 2:**
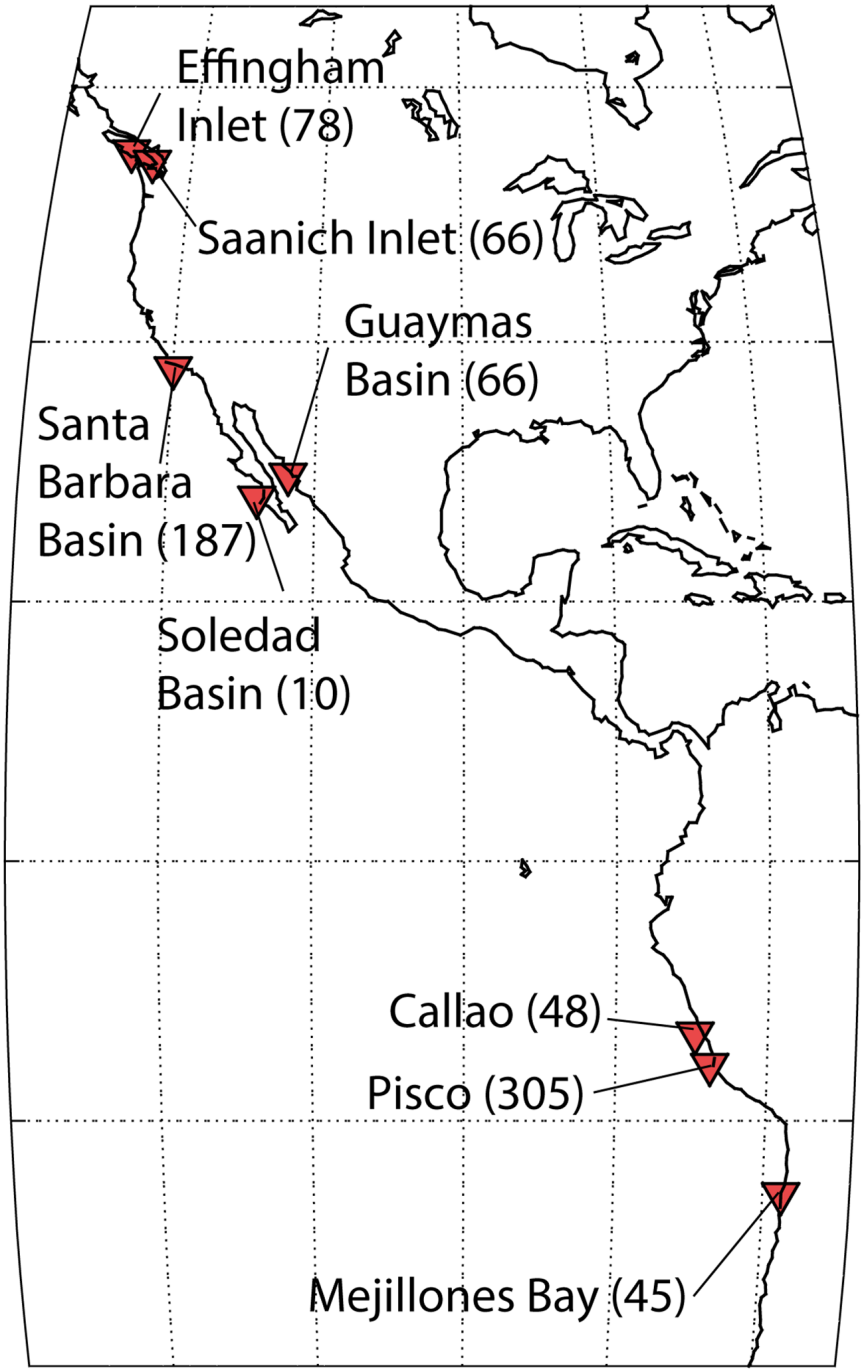
The eight sites examined in this study. The total number of record pairs at each are listed in parentheses. A ‘record’ is defined as a single parameter measured in the sediment, and a ‘record pair’ is constituted by two co-existing records that can be compared.

A search was performed for published fish debris in recent marine sediments, using four online databases: NODC (nodc.noaa.gov), Pangaea (pangaea.de), Web of Science (webofscience.com), and Google Scholar (scholar.google.com). The search revealed nine locations at which counts of sedimentary fish bones, otoliths and/or fish scales were available, located along the west coast of North and South America (Fig 2) and in the Yellow Sea [48]. We refer to each of these locations as a ‘site’. The same databases were then searched for any biogeochemically relevant data that had been collected at these sites, including all cores within 10 km distance that overlapped in time with the fish abundance records. This resulted in positive data identification at all sites except the Yellow Sea. Data were obtained from online databases if available, otherwise by manually digitizing the values in published figures or by contacting study authors directly. We also retained climate-relevant proxy data at the eight sites where biogeochemical and/or physical climate proxy data were available, although we did not conduct an exhaustive search for these.

Most of the sites had been sampled by multiple sediment cores, and the composition of most sediment cores had been analyzed in terms of multiple observable parameters at many depths below the sediment-water interface. We refer to each depth-parameter series as a ‘record’. For many cores, chronological information had previously been used to estimate the ages of the samples, providing an ‘age model’. In most cases, records were reported with depth as the primary axis, but in some cases the primary axis was the estimated age. All records retrieved for the study are listed in Table 1 and data files are provided as supplementary material.

**Table 1:** Data sources for the eight sites examined. Italicized proxy names indicate those for which only flux data was available.

Site	Core	Ref.	Record types	Period (CE)	Mean sample interval (years)
Effingham Inlet, Canada	TUL99B03	[49]	Fish scale counts	-2532-562	47
Effingham Inlet, Canada	TUL99B03	[50]	Age model	-2745-1805	568
Effingham Inlet, Canada	MD02-2494	[51]	TOC, Opal, CaCO3, total N, N-15, redox sensitive elements	-15526-1214	22
Effingham Inlet, Canada	MD02-2494	[52]	Age model	-15526-1214	22
Saanich Inlet, Canada	ODP 1034	[53]	Fish bone counts	-10964-1121	50
Saanich Inlet, Canada	ODP 1034	[54]	TOC	-9685-1418	113
Saanich Inlet, Canada	ODP 1033	[55]	TOC, Opal, CaCO3, total N, N-15, redox sensitive elements, C-13	-9684-1418	113
Santa Barbara Basin, USA	214	[56]	Fish scale counts	145-1995	10
Santa Barbara Basin, USA	SPR0901-02KC	[57]	Fish scale counts	1009-1492	6
Santa Barbara Basin, USA	SABA87-1	[58]	TOC, alkenone SST	1443 -1941	2
Santa Barbara Basin, USA	ODP 893	[59]	TOC	-46681-1919	173
Santa Barbara Basin, USA	ODP1017	[60]	N-15, TOC	-72801-1950	150
Santa Barbara Basin, USA	MD2503	[61]	Foram counts	-31583-1786	107
Santa Barbara Basin, USA	MD2504	[61]	Foram counts	-22429-1769	125
Santa Barbara Basin, USA	BC-1	[42]	*Otoliths*	40-2000	10
Soledad Basin, Mexico	244	[39]	*Fish scale accumulation*	1783-1976	5
Soledad Basin, Mexico	244	[62]	Age Model	1725-1976	5
Soledad Basin, Mexico	TUL (unnamed)	[63]	*TOC*	1456-1977	18
Guaymas Basin, Mexico	7807-1305	[64]	*Fish scale accumulation*	1735-1975	10
Guaymas Basin, Mexico	BC50	[65]	TOC, Opal, total N, redox sensitive elements	1814-1987	2.7
Callao, Peru	106KL	[66]	Alkenone SST	-17654-1960	178
Callao, Peru	B0405-13	[67]	Fish scale counts, fish bone counts, TOC, CaCO3, quartz, N-15	1309-1999	7
Callao, Peru	C0329	[68]	Fish scale counts, P_fish, TOC, CaCO3, Opal, total N	No Age Model	-
Callao, Peru	ODP1228	[69]	N-15, TN	-12675 to 1088	98
Callao, Peru	W7706-40	[70]	N-15, TN	-2569-1607	24
Callao, Peru	SO78-173-4	[71]	TOC, SST	No Age Model	-
Pisco, Peru	B0405-06	[72]	Fish scale counts, fish bone counts, TOC, CaCO3, quartz, N-15	1291-1998	5
Pisco, Peru	B0405-06	[73]	Alkenone SST	1737-2003	3
Pisco, Peru	B0405-06	[41]	Fish scale counts	1291-1998	5
Pisco, Peru	B0506-14	[67]	Fish scale counts	1510-2005	1
Pisco, Peru	B0506-14	[45]	Opal, N-15, TOC, redox sensitive elements	1510 -2005	2
Pisco, Peru	B05-13	[45]	Fish scale counts, TOC	1858-2004	1
Mejillones Bay, Chile	F981A	[74]	*Fish scale accumulation, TOC*, N-15	1746-2001	3
Mejillones Bay, Chile	BC3D	[75]	TOC	1787-2002	3
Mejillones Bay, Chile	33C	[76]	Fish scale counts, TOC, CaCO3, Opal	No Age Model	-
Mejillones Bay, Chile	BC-1	[77]	*Fish scale accumulation*, TOC, Quartz, SST	1331-2013	6

To provide an objective assessment of the statistical relationships between different sedimentary measurements, we performed regressions of all possible record pairings. First, comparisons between proxies were made within the same core when available, as reported vs. depth in sediment, interpolating linearly between depths when necessary. For these ‘incore’ comparisons there are no age model uncertainties. Comparisons were also made between different cores at the same site, using published age models, with the assumption that a given site represents a sufficiently small oceanic region that changes in the ecosystem, biogeochemistry and physical ocean state were synchronous on the multi-annual sampling timescale. Proxies from cores without age models were only ever compared to other records from the same core (vs. depth in sediment). Correlations calculated between cores should be viewed with caution as all are from areas of high-sedimentation rates and many sites are prone to frequent sediment slumps and discontinuities [78]. We assume that the inevitable age model inaccuracies—which may be large—will tend to lead to the detection of fewer correlations than exist in reality, since we cannot readily conceive of instances in which chronological error would systematically produce additional inter-core correlations.

In addition, we point out that there is often a very broad range of timescales and sampling resolutions among records at the same site. This range of timescales may have a significant influence on the processes they can capture, as well as the relative strengths among multiple co-existing processes that vary with different temporal frequencies. For example, if the effect of fish populations on sinking particle fluxes varies strongly on a 5-y timescale, it may not be strongly-resolved in a record with a 50-y sampling interval. Alternatively, if large community-driven changes alter sinking particle fluxes on a regional, multi-decadal timescale, these may be overwhelmed by interannual fluctuations of local hydrography in a record with annual resolution.

Linear interpolation between records was carried out in both directions, so that each record-pair was analyzed twice. We chose *n* > 8 as the minimum number of overlapping data points among two records in order to consider them as a record-pair. Relationships between proxy timeseries were quantified using linear regression, and correlations with *p* values less than 0.05 were identified as statistically significant [79]. Each timeseries was also linearly detrended and correlations recalculated; if the *p* value remained below the threshold of 0.05 the correlation was identified as a significant detrended regression. The detrending test was performed given the occurence of long-term signals (such as gradual climate shifts or isostatic changes altering the shape of coastal embayments) that could affect both fish abundance and biogeochemical proxies without any causal relationship between them. Whenever an original study identified individual species in fish scale counts, correlations were calculated with each species as well as the sum of all scales.

Comparisons within cores were always made between pairs of concentrations or pairs of accumulation rates. For the analyses of age-based pairs, we relaxed this requirement. When both fluxes and concentrations were available for a single parameter, we only included the concentrations. This choice was made to avoid false positive correlations due to changes in the estimated sedimentation rate at chronological tie points, though we note that dilution by other sediment constituents could also produce positive correlations between proxies (see also Discussion). If the concentrations were not available but could be calculated from the fluxes using other sedimentary parameters, we did so. The records for which we used fluxes are listed in italics in Table 1.

Both TOC and fish scales decay due to microbial activity, and changing preservation regimes over time might have altered the preserved abundances at any of the sites. Previous work has evaluated fish scale preservation at two of the sites, Saanich Inlet [53] and the Peruvian margin [41]. Changing preservation appears to be a particularly important issue in the Peruvian cores, where a pronounced biogeochemical shift has been identified in the mid-19th century, associated with a shift from poor preservation to excellent preservation (see S1 Appendix for a detailed explanation of this analysis). We therefore only consider Peruvian sediments from the well-preserved period in the analyses discussed in the main text. Although we do not make similar subdivisions of other records, it should be born in mind that this is due to a lack of information, and that variable preservation could be a relevant factor at any of the sites.

## Results

Table 2 provides an overview of the regression analysis results, for both within-core and age-based record pairs. In all cases except Fish-Physics pairs (for which few within-record pairs are available), the proportion of significant correlations is higher for within-core pairs, as would be expected from the introduction of age-model error in age-based pairs. The two groups do not include the same records, given that many more pairs are included in the age-based group, yet the following general features are robust between them, including both raw and detrended records: the strongest correlations are found among biogeochemistry-biogeochemistry pairs, followed by physics-biogeochemistry pairs, followed by fish-fish pairs, followed by fish-biogeochemistry pairs, followed by fish-physics pairs. At most sites we included only one physical proxy record, so the physics-physics pairs were not well tested. The total number of record pairs varies markedly between sites (Fig 2), so that this overview is dominated by the sites with larger numbers of pairs, most notably Pisco and Santa Barbara Basin. Detailed correlation tables for each site are provided in the S1 Table and S1 File.

**Table 2:** Overview of all record pairs analyzed at the eight sites. The first column gives the total number of record pairs of each type. The second and third columns give the pairs for which significant correlations were identified, as total number and fractional percentage, respectively. The fourth and fifth columns correspond to the second and third columns, but for detrended records. The upper portion of the table only includes record pairs that occurred within the same sediment core, while the lower portion includes all available record pairs at a site that included an age model.

	Pairs	Sig.	Sig. %	Detrend sig.	Detrend sig. %
Within core					
Fish-Fish	92	29	32	30	33
Fish-Bgc	68	13	19	7	10
Bgc-Bgc	156	119	76	90	58
Fish-Phys	7	0	0	0	0
Phys-Bgc	33	24	73	17	52
Phys-Phys	2	0	0	0	0
Age-based					
FishFish	183	53	29	49	27
FishBgc	301	41	14	29	10
BgcBgc	208	137	66	94	45
FishPhys	56	4	7	4	7
PhysBgc	60	28	47	18	30
PhysPhys	2	0	0	0	0

Table 3 focuses on the results for fish-biogeochemistry and fish-physics pairs. The correlations are given in terms of number of record pairs, each of which represents a comparison of fish scale abundance with another proxy record at the same site. Note that multiple taxon-specific fish records were available at many sites, so that the total number of pairs is much greater than the total number of non-fish proxy records.

**Table 3:** Correlations between fish abundance and other proxy records for age-based pairs. The number of significant (Sig., *p* < 0.05) correlations are listed for each available proxy record vs. all fish abundance records at the same site. Of these, the number of positive (i.e. *r* > 0) regression coefficients are indicated in the ‘Sig.+ve’ column, and the percentage of significant correlations among the total pairs tested is given in the ‘Sig.%’ column. The ‘dt’ columns show the corresponding values for the linearly-detrended records.

	Pairs	Sig.	Sig +ve	Sig.%	Sig.dt	Sig.dt +ve	Sig.dt%
TOC	85	15	8	18	12	4	14
TN	29	3	1	10	1	0	3
CN	10	2	2	20	1	1	10
CaCO_3_	22	2	2	9	3	2	13
Opal	19	7	1	37	3	1	16
d15N	53	5	5	9	5	5	9
ForamAssemb	12	1	0	8	0	0	0
Productivity	9	1	1	11	0	0	0
Bottom Water O_2_	62	4	2	6	3	2	5
Quartz	22	2	2	9	4	2	18
SST	27	1	0	4	0	0	0
Al	7	1	1	14	0	0	0

Among the fish-bgc and fish-physics pairs, those with significance occurring in more than 10% of pairs (including after detrending) are TOC, the C:N ratio and opal. Among these, correlations with C:N are positive, while TOC and opal include both positive and negative correlations. Conversely, SST, foram assemblages and productivity indicators all show very low occurrence rates of significant pairs. The absence of correlations with SST is surprising, given the common assumption that SST reflects upwelling and productivity (e.g. [80]), which might therefore provide a bottom-up forcing on fish abundance. The rarity of correlations with the available productivity proxies also fails to support an important role for local primary production in driving fluctuations of fish abundance. Nonetheless, given the small number of records available for each proxy, and the weakness of most correlations where they do exist, the general impression provided by this overview must be viewed with caution.

Table 4 provides details of all correlations identified as significant among the fish-bgc and fish-physics pairs. The table shows that a number of the correlations are not consistently significant between the original and detrended timeseries, which we take as an indication that they are less robust. In particular, all but one of the record pairs in Saanich fails to produce significant detrended correlations. On the other hand, some of the record pairs show very significant correlations (*p* < 0.01) with both. The Mejillones Bay records have especially strong positive correlations between fish scales, TOC and *δ*^15^N.

**Table 4:** All significant fish correlations. For each record pair for which a significant correlation was found, the proxy names, corresponding cores (single names indicating within-core pairs), and correlation statistics *r* and *p* are given. The *dt* columns are for detrended records. The total number of points in each comparison timeseries is given by *n*. Boldface indicates record pairs for which *n* > 20 and *p* < 0.01 for both original and detrended pairs. Note that “total scales” is the sum of available records and may not include unpublished or uncounted species. Multiple Anchovy-TOC pairs exist at Pisco due to their coverage of different time periods.

Fish	Proxy	Cores	r	p	dt r	dt p	n
**Callao**							
Hake scales	TN	B0405-13	0.422	0.010	-	-	35
Hake scales	TOC	B0405-13	0.460	0.004	-	-	35
**Effingham**							
Herring scales	CaCO_3_	TUL99B03—MD02-2494	0.291	0.012	0.243	0.026	66
**Guaymas**							
Anchovy scales	TN	7807-1305—BC50	-0.599	0.004	-0.576	0.005	17
4 Anchovy scales	C:N	7807-1305—BC50	0.621	0.003	0.685	0.002	17
**Mejillones**							
**Anchovy scales**	**TOC**	F981A	0.565	**0.000**	0.437	**0.000**	**83**
**Sardine scales**	**TOC**	F981A	0.365	**0.001**	0.454	**0.000**	**83**
**Anchovy scales**	***δ*^15^N**	F981A	0.518	**0.000**	0.355	**0.002**	**82**
Sardine scales	*δ*^15^N	F981A	0.315	0.012	0.409	0.000	82
**Anchovy scales**	**TOC**	F981A—BC-3D	0.456	**0.000**	0.375	**0.001**	**70**
Anchovy scales	SST	BC-1	-0.226	0.035	-	-	104
Sardine scales	Quartz	F981A—BC-1	0.447	0.019	0.460	0.013	83
**Pisco**							
**Sardine scales**	**CaCO_3_**	B05-13—B0405-06	0.357	**0.000**	0.363	**0.000**	**131**
Sardine scales	Quartz	B0506-14—B0405-06	0.175	0.047	0.200	.023	129
Sardine scales	TOC	B0405-06—B0506-14	0.376	0.009	-	-	47
Jack Mackerel scales	TOC	B0405-06—B0506-14	-0.300	0.040	-	-	47
Jack Mackerel scales	CaCO_3_	B0405-06	-	-	-0.339	0.038	51
Anchovy scales	TOC	B0506-14	0.261	0.003	-	-	132
**Hake scales**	**TOC**	B0506-14	0.318	**0.000**	0.454	**0.000**	**132**
Total scales	TOC	B0506-14	0.291	0.001	-	-	132
Hake scales	Opal	B0405-06—B0506-14	-	-	-0.340	0.012	47
Jack Mackerel scales	Opal	B0405-06—B0506-14	0.345	0.017	0.295	0.044	47
**Hake scales**	**Opal**	**B0506-14**	-0.261	**0.003**	-0.374	**0.000**	**131**
Anchovy scales	Opal	B0506-14	-0.271	0.002	-	-	131
Total scales	Opal	B0506-14	-0.299	0.001	-	-	131
Anchovy scales	Re/Mo	B0506-14	-	-	-0.230	0.013	130
Hake scales	Re/Mo	**B0506-14**	0.263	**0.002**	0.377	**0.000**	130
Anchovy scales	TOC	B0506-14—B05-13	-0.575	0.040	-	-	13
Hake scales	Re/Mo	B05-13—B0506-14	0.219	0.016	0.303	0.001	121
Total scales	Opal	B05-13—B0506-14	-0.262	0.003	-	-	123
Anchovy Scales	Opal	B05-13—B0506-14	-0.244	0.007	-	-	123
Anchovy scales	TOC	B0506-14—B0405-06	-	-	-0.307	0.001	128
Total scales	TOC	B0506-14—B0405-06	-	-	-0.297	0.001	128
Anchovy scales	Quartz	B0506-14—B0405-06	-	-	-0.440	0.000	129
Total scales	Quartz	B0506-14—B0405-06	-	-	-0.423	0.000	129
Anchovy scales	TOC	B05-13—B0506-14	-	-	-0.314	0.000	124
Total scales	TOC	B05-13—B0506-14	-	-	-0.284	0.001	124
**Saanich**							
Bones	TOC	ODP1034	-0.575	0.001	-	-	206
Bones	TOC	ODP1034—ODP1033	-0.356	0.000	-	-	223
Bones	TN	ODP1034—ODP1033	-0.480	0.000	-	-	223
Bones	Opal	ODP1034—ODP1033	-0.394	0.000	-	-	223
Bones	C-13	ODP1034—ODP1033	-0.297	0.001	0.266	0.020	223
Bones	Al	ODP1034—ODP1033	0.424	0.000	-	-	223
Bones	Mo	ODP1034—ODP1033	-0.465	0.000	-	-	223
Bones	Mo/Al	ODP1034—ODP1033	-0.495	0.000	-	-	223
Bones	C:N	ODP1034—ODP1033	0.476	0.000	-	-	223
**Santa Barbara**							
Sardine scales	TOC	214—SABA87-1	-	-	-0.242	0.020	48
Total scales	TOC	214—SABA87-1	-0.274	0.019	-0.250	0.025	50
Hake scales	*δ*^15^N	214—SMB	0.773	0.000	0.543	0.018	14
Total scales	*δ*^15^N	214—SMB	0.810	0.000	0.670	0.002	14
Total scales	Benthic forams	214—MD2504	0.481	0.011	-	-	163
Otoliths	TOC	SABA87-1—BC-1	-	-	-0.294	0.014	229
Anchovy scales	TOC	214—SABA87-1	-0.527	0.000	-	-	50
Anchovy scales	*δ*^15^N	214—SMB1	0.772	0.001	-	-	14
**Soledad**							
Hake scales	TOC	244—TUL	-0.518	0.001	-0.475	0.002	39

In general, TOC stands out as having a large proportion of significant correlations with fish abundance, and is the only non-fish proxy that was available at all sites. We therefore discuss the correlations with TOC in greater detail.

### Organic carbon

Of the eight sites examined, statistically significant negative correlations between fish abundance proxies and TOC were found in cores at three sites (Santa Barbara, Soledad, Saanich Inlet), positive correlations at two sites (Callao, Mejillones), both positive and negative correlations at one site (Pisco), and no significant relationships at two sites (Guaymas, Effingham).

The first site with negative correlations between TOC and fish is Santa Barbara Basin, where an aggregate of all fish scales produces a negative correlation to TOC (*r* = −0.274, *p* = .019) across numerous boom-bust cycles in anchovy scales. Interestingly, there was no correlation between the TOC record and the U^*k*^’_37_ derived SST at the same site [58].

Further south at Soledad Basin, accumulation rates of hake scales have a strong negative correlation with TOC flux (*r* = −0.518, *p* = .001), which remains robust after detrending (*r* = −0.475, *p* = .002), whereas there is not a significant correlation with other species. Although it is conceivable that changes in scale accumulation rates could dilute TOC concentrations, the fact that no correlation exists with the total scale accumulation rate suggests that this is unlikely, and the ecological or habitat-based characteristics of hake may be significant at this site.

Saanich Inlet, on the southern tip of Vancouver Island, also has a negative correlation between fish abundance (fish bone counts) and TOC values, in a record that spans the entire Holocene. A gradual increase in TOC and decrease in fish bones from approximately 7500 to 3000 BCE produces an overall negative correlation (*r* = −0.58, *p* = 0.001) but which is insignificant when detrended. Isostatic rebound following the last deglaciation gradually isolated the fjord from the open ocean, increasing productivity and lowering oxygen conditions [55]. This major bathymetric change likely dominates the trends in all records from this site, explaining the numerous strong correlations that disappear following detrending.

The final site with negative correlations is Pisco, in the Peruvian Upwelling Zone; however, there is a mix of both negative and positive correlations at this site. Inconsistencies in the correlations between cores may be due to differences between species, as well as unrecognized sedimentary hiatuses that plague the Peruvian margin, as discussed by [78]. It is also notable that both cores show a marked decrease in anchovy scales and increase of TOC within the upper sediment. This decrease of anchovy scales has been attributed to low anchovy biomass during a sardine-dominated period [81] and subsequent biomass removal by industrial fishing, and roughly follows reported anchovy landings [41, 72, 77].

In contrast to the equivocal sign of correlations in Pisco, the correlations at Mejillones Bay in Northern Chile are strongly positive for both anchovy and sardine scales, with *r*-values ranging from 0.37 to 0.57 and *p*-values all less than 0.001. Although a few of the record pairs did not show significant correlations, particularly the anchovy scale record from BC-1, overall these stand out as the most consistent and powerful fish-TOC correlations among the sites analyzed. The second site with a positive correlation is Callao, a second Peruvian site where the 19th-century biogeochemical transition was also previously identified. One significant positive correlation occurred here, with hake scales, but it was relatively weak and did not remain significant following linear detrending.

The downcore record from Guaymas Basin produced no statistically significant correlation between fish abundance and TOC. Likewise, Effingham Inlet, a small (<1 km wide) isolated fjord on the west coast of Vancouver Island, showed overall low scale counts with no obvious periodicity or relationship with TOC.

## Discussion

Our broad statistical overview of published sediment records shows that most of the possible pairings of fish abundance and biogeochemical proxies are not significantly correlated. This is not surprising, given the complexity of the marine ecosystem and the potential for sedimentological processes to obscure relationships. Nonetheless, we find that significant correlations occur with a sufficient frequency among some record pair types that they are very likely to represent causal relationships. It would therefore appear that marine sediment records do have the potential to inform poorly-understood relationships between fish and biogeochemical cycling. These interactions could include bottom-up limitation of fish by primary production, interactions with dissolved oxygen, or the trophic cascades discussed in the introduction.

Correlations between fish abundance records and TOC are observed, at statistically-significant rates (including following detrending), at four of the eight sites at which records were available. The correlations are unlikely to have arisen from the same mechanisms at all sites, given diversity among the environments and the ecosystems, as well as differences in sedimentation. The range of records investigated also differs greatly in the temporal resolution (Table 1), which will result in different sensitivities to mechanisms, given different timescales of interactions between ecosystems and biogeochemistry. Additionally, we would caution against strong conclusions being drawn from these correlations, given that the number of records is small, and even where significant, the fraction of variability explained tends to be small (*r*^2^ < 0.5). Nonetheless, the identification of a correlation with *p* < 0.05 would be expected to occur less than once in 20 cases by random chance, and although correlations do not necessarily imply direct causal relationships, it is interesting to further consider the possible underlying mechanisms.

**Fig 3:**
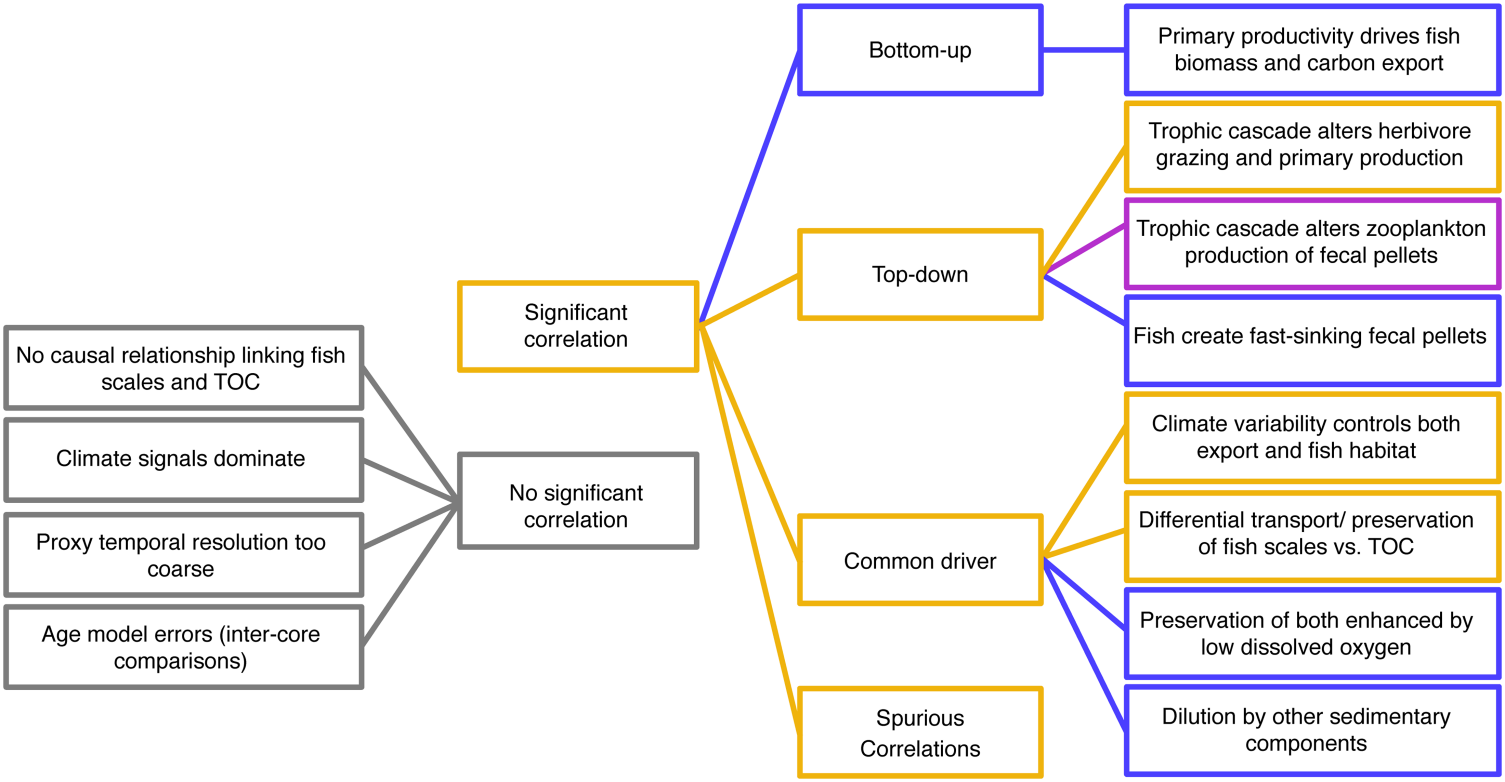
Potential mechanistic explanations of observed correlations between TOC and fish debris. Grey indicates no correlation is expected, blue a positive correlation, purple a negative correlation, and orange indicates either positive or negative correlation.

Given that TOC is the most widely-available proxy, we focus the discussion on the possible mechanisms that might have generated correlations between proxies of fish abundance and TOC. This discussion is intended to provide a framework for considering possible implications of the observed correlations and to help direct future study. We divide the possible mechanisms into four categories (Fig 3) as described below.

### Bottom-up control: Organic carbon affects fish

A positive correlation would be expected in an ecosystem where temporal variability of fish biomass is driven by variability in the local primary production. During times of higher primary production, the increased food availability would percolate up the food web, resulting in greater total fish abundance. For a positive correlation to be found, greater primary production must also result in enhanced carbon burial in the sediments, requiring that particulate export production (i.e. the sinking of organic matter out of the sunlit surface layer) also increased.

Mejillones Bay showed very robust positive correlations between TOC and fish scales (both sardine and anchovy) that would support a local bottom-up control, consistent with the interpretation of [77] based on a longer record of scale deposition rates from the site. The negative correlation of anchovy scales with SST, and positive correlation of sardine scales with quartz, are also consistent with a wind-driven upwelling control on productivity at this particular site. These results are entirely consistent with the interpretation of [77]. We also found positive correlations over the complete timeseries from the Peruvian sites (including sediments prior to and following the mid-19th century oxygenation change), as discussed in the S1 Appendix, consistent with the upwelling-driven bottom-up control proposed by [45, 72, 73] based on multiple proxies from Peru and elsewhere. However, given the evidence for a large change in preservation of both TOC and scales [41, 72], we cannot rule out the possibility that a major portion of this correlation is due to the preservation change. We also find positive correlations for some fish species in B0506-14 from Pisco within the well-preserved interval, but these co-occur with negative correlations. The weakness or absence of positive correlations at sites other than Mejillones Bay should not be taken as evidence that changes in primary production are unimportant for fish abundance, they simply do not emerge unambiguously from the analysis. Thus, although bottom-up control undoubtedly plays a role in fish abundance between locations [2] and appears to play a strong role at Mejillones [77], it does not appear to have dominated fish abundance variability on the temporal and spatial scales captured by the available sediment records at the other sites.

### Top-down control: Fish affect organic carbon

A second possibility is that fish exert a control on carbon export to the sediment, an example of top-down control. This control on carbon export could be a result of predation on lower trophic levels (trophic cascade), or due to the behaviour or the species itself. For example, predation by planktivorous fish would be expected to deplete the stocks of herbivorous zooplankton, potentially reducing their repackaging of phytodetritus as fecal pellets, which sink rapidly to sediment, as schematized in Fig 1. This would result in a smaller carbon flux to sediments when planktivorous fish are abundant, which would produce a negative correlation. However, one could conceive of other possibilities as well. Since zooplankton grazing limits the phytoplankton biomass, a larger zooplankton population could reduce local productivity in a region with excess nutrients, such that less organic carbon would end up in the sediment [5, 23]. Or the abundance of piscivorous fish could have an impact on the abundance or behaviour of smaller fish, the effects of which may not be straightforward. It thus seems possible that abundant fish stocks could either enhance or diminish carbon export to sediment: both would appear possible, depending on interactions between fish, zooplankton, phytoplankton and the spectrum of sinking particles within the local community.

It is possible that at least some of the significant relationships observed between fish and TOC reflect top-down control. The results suggest a species-dependence of results at both Santa Barbara basin and Pisco, which could be because species have different relationships with the local food web. Alternatively, it might reflect a role for common drivers.

### Common environmental drivers

Correlated changes of fish scale counts and TOC may also be driven by a common factor, without a direct causal link between the two. For example, all eight sites are directly or indirectly affected by coastal upwelling, which can simultaneously affect water column oxygenation [82] and organic matter production. A depletion of oxygen within the water column could alter the distribution of bottom-dwelling species and compress the habitat depth range of pelagic fish, making them more vulnerable to predation. The latter mechanism has been invoked to explain positive correlations between oxygen concentrations and pelagic fish stocks in the California Current over the last 60 years [83]: as more oxygen-poor, nutrient-rich waters were upwelled, primary production is expected to have increased. The result is co-occurence of increased phytodetritus, better preservation of organic matter in sediments affected by the bottom water oxygen decline, and depleted fish populations.

Changes in water column oxygenation affect species of fish differently and can trigger a shift from one regime to another. In the Humboldt current system, it has been suggested that multidecadal oxygen changes drove shifts between anchovy and sardine regimes [81]. Low-oxygen conditions are associated with anchovy dominance and vice versa for sardines [36]. This introduces a large degree of uncertainty in how a change in water column oxygen would affect carbon export as it is also a feasible mechanism to create negative correlations between TOC and fish scales depending on how each individual species affects export production.

It seems quite feasible that upwelling-oxygen dynamics were responsible for the strong and highly significant negative correlations between TOC and Hake scales in the Soledad Basin. If greater production occurs due to an influx of nutrients supplied by stronger upwelling, the associated shoaling of isopycnals would draw low-O_2_ waters up to shallower depths [82]. The expansion of low-oxygen waters on the upper slope could reduce the habitat of demersal hake and lead to an anti-correlation between hake abundance and TOC. A relationship with bottom water oxygen is also suggested by the weak positive correlation (*r* < 0.2) of hake scales at Pisco with Re/Mo, a bottom water oxygen proxy. This suggests that weak upwelling at Pisco reduces primary production, while simultaneously improving the local oxygenation of bottom waters, which could increase the abundance of hake. Yet, this may be a site-specific feature, given that none of the other five sites where some kind of oxygen proxy is available shows any statistically significant correlations with any kind of fish abundance records (S1 Table). Local interactions between fish stocks and dissolved oxygen distributions may depend on the the bathymetry and geometry and intensity of oxygen minimum zones relative to the core locations, and could involve more complex dynamics than the simple causality evoked here [84].

Another possible common driver involves processes involved in the generation of the sedimentary records. The sedimentation and burial of TOC and fish scales is not instantaneous, and sedimentary processes act upon these proxies differently. Local currents transport sediment along the seafloor and winnow the finer sediments away from the coarser; this hydraulic sorting may differentially transport the fine fraction of TOC relative to fish scales. A potentially important issue is any preservation difference in the sediment of organic carbon originating from phytodetritus or marine snow versus fecal pellets from zooplankton or fish, which may generate positive or negative correlations. Positive correlations could occur due to a common influence of redox conditions on both TOC and fish scale preservation; as discussed in the S1 Appendix, changes in preservation driven by variable oxygenation also appear to have played a major role in the Peruvian sediment records before and after approximately 1820. The possibility of changes in TOC preservation could be further tested by comparison with carbon flux proxies less prone to diagenetic alteration, such as Ni [85], or by more detailed characterization of the sedimentary organic matter [86].

Since we analyzed both flux and concentration records, it is possible that variable dilution of both TOC and fish debris by changes in other sedimentary components contributes to the positive correlations identified in some cases. It is also possible that negative correlations arise due to the dilution of TOC concentrations by fish scales, given that TOC fluctuations are typically on the order of 1%, while fish scale abundance varies by orders of magnitude. However, if dilution were the driving factor, we would expect the strongest negative correlation to always be with an aggregate of all scales counted rather than individual species, whereas the opposite is more commonly observed in the available cases here.

### Spurious correlations

The occurrence of spurious correlations (i.e. no causal link) must also be considered. Apparent correlations would be expected to occur among unrelated timeseries at a rate equal to the p-value used for significance testing; in our case, < 5%. In addition, correlations between causally-unrelated timeseries are more likely in the presence of long-term trends. For example, a decrease of TOC with depth below the core-top could be produced by the downcore decay of organic carbon by microbial degradation (diagenesis). Depending on sedimentation rates, this could occur over the recent period of rapid intensification of industrial fishing, which would produce a decrease in scales towards the top of the core. Thus, causally-unrelated effects of microbial degradation and fishing could produce a spurious negative correlation. This is more likely to occur between records with strong linear trends (e.g. the Saanich records), since more complex temporal patterns are less likely to change in a coordinated fashion, thereby prompting our inclusion of detrended correlations. On the other hand, if fish do exert a top-down control, the onset of industrial fishing could have actually caused the increases over time in sedimentary TOC, in which case the hypothetical correlation would not be spurious.

### No correlation observed

Finally, at half of the sites, significant correlations between TOC and fish scales were not observed. This lack of correlation may indicate that the ecosystem structure at these sites does not allow for a consistent relationship between fish abundance and carbon export. Alternatively, relationships may be masked by larger climate signals (such as the large shifts seen in the Saanich site), obscured by sedimentological artifacts, or lost in the translation of measurements between cores, as necessitated by the approach here. An absence of correlations cannot be taken as an absence of links between proxy variables and fish abundance.

### Outlook for future work

The sediment record represents an important archive that can be used to help examine long-term relationships between marine fish and the environment around them, bridging the gap between marine ecology and biogeochemistry. However, there are currently very few published studies that include both biogeochemical proxy measurements and fish abundance proxies on the same sediment cores. To take full advantage of the opportunity, more datasets of fish abundance should be developed, as they are currently sparse relative to other sedimentary proxies, and they should be accompanied by multiproxy data on the same sediments. Although the present work focused mostly on fish scales, which break down relatively quickly in most marine sediments, fish teeth and bones are potentially preserved for much longer time periods, providing the possibility to extend the record of fish abundances back thousands or millions of years [13, 87]. Ideally, fish abundance records would be directly accompanied by measurements of biogeochemical and physical proxies, in order to avoid the inter-core comparisons that were necessary here. Finally, all of the sites examined here underlie coastal upwelling zones in the eastern Pacific, because of their tendency to generate rapidly-accumulating oxygen-poor sediments that preserve excellent fish scale records; of particular interest would be other types of fish abundance records that can occur in dramatically different oceanographic regimes (e.g. otoliths, teeth), to examine ecosystem-biogeochemistry links in other settings.

## Conclusions

Our compilation of fish abundance records revealed significant correlations between fish debris and the available biogeochemical proxies at most sites, though they were less frequent than correlations among biogeochemical proxies alone, or between biogeochemical proxies and physical proxies. Most notably, significant correlations between fish scales and TOC, that persisted when linearly detrended, occurred at four of the eight sites. Our meta-analysis cannot definitively distinguish among the causal mechanisms behind the observed fish-TOC correlations, but suggests that they are dominated by site-specific factors.

At Mejillones Bay, strong positive correlations between fish scales and TOC are consistent with bottom-up forcing, whereby greater upwelling provides more food for the ecosystem, increasing the abundance of all fish, as concluded by the authors of the original work [77]. These significant positive correlations could also conceivably reflect a top-down trophic cascade, via predation on herbivorous plankton, leading to larger phytoplankton biomass. Meanwhile, two possibilities to explain the negative relationships found at Soledad, Santa Barbara and Pisco are: 1) that abundant fish populations reduce the organic carbon flux to sediments by contributing to more complete organic matter recycling within the water column [5], or 2) that upwelling-driven oxygen-nutrient dynamics simultaneously control the habitat ranges of fish and nutrient supply to primary producers [83].

Although we cannot draw firm conclusions based on the data in hand, we find promising signs that the development of more multi-proxy records of fish abundance and biogeochemistry could provide valuable insights on the links between marine fish populations and biogeochemical cycles, and their variations through Earth history.

## Supporting information

S1 Text Overview. An overview of the data contained within Supporting Information.
(PDF)

S1 Table Site specific correlation Tables. Tables outlining the number and nature of significant correlations at each site.
(PDF)

S1 Fig Site specific correlation Figures. Matrix correlation plots of all proxy comparisons made at each site.
(PDF)

S1 Appendix Preservation concerns. An outline of the preservation concerns at the Peruvian sites.
(PDF)

S1 File Analysis code. A zipped archive of the complete MATLAB analysis code used in this paper.
(ZIP)

S2 File Downcore plots and correlation Tables. A zipped archive of all down-core plots and correlation tables used in this paper.
(ZIP)

S3 File RawInputData. A zipped archive of the raw input data used at each site.
(ZIP)
