# Supplementary material for: Links between fish abundance and ocean biogeochemistry as recorded in marine sediments
Source: PLoS One. 2018 Aug 1;13(8):e0199420. doi: 10.1371/journal.pone.0199420 (PMC6070179; doi:10.1371/journal.pone.0199420)
Supplement: S1 Text — An overview of the data contained within Supporting Information. (PDF) [file pone.0199420.s001.pdf]

S1 SI Overview

The following supporting information includes correlation coefficient matrix figures for every comparison made in this manuscript as well as tables summarizing the number of significant correlations between each category or proxy and specific proxies when compared to proxies of fish abundance. In addition to the information presented here, we are also including a compressed archive that consists of down-core plots of all comparisons made along with the raw data assembled from the literature.
