# Supplementary material for: Links between fish abundance and ocean biogeochemistry as recorded in marine sediments
Source: PLoS One. 2018 Aug 1;13(8):e0199420. doi: 10.1371/journal.pone.0199420 (PMC6070179; doi:10.1371/journal.pone.0199420)
Supplement: S1 Table — Tables outlining the number and nature of significant correlations at each site. (PDF) [file pone.0199420.s002.pdf]

S2 Site Specific Correlation Tables

	Pairs	Significant	Sig. detrend
FishFish	6	1	1
FishBgc	16	2	0
BgcBgc	14	6	3
FishPhys	4	0	0
PhysBgc	8	7	3
PhysPhys	0	0	0

Table 1: Callao age-based record pairs.

	Pairs	Sig	Sig. +ve	Sig. %	Sig. detrend	Sig. detrend +ve	Sig. detrend %
TOC	4	1	1	25	0	0	0
TN	4	1	1	25	0	0	0
CN	0	0	0	-	0	0	-
Carb	4	0	0	0	0	0	0
Opal	0	0	0	-	0	0	-
d15N	4	0	0	0	0	0	0
d13C	0	0	0	-	0	0	-
ForamAssemb	0	0	0	-	0	0	-
Productivity	0	0	0	-	0	0	-
BWO2	0	0	0	-	0	0	-
Quartz	4	0	0	0	0	0	0
SST	0	0	0	-	0	0	-
Al	0	0	0	-	0	0	-

Table 2: Callao Fish-bgc and Fish-phys age-based record pairs.

	Pairs	Significant	Sig. detrend
FishFish	3	2	2
FishBgc	27	1	1
BgcBgc	36	26	24
FishPhys	3	0	0
PhysBgc	9	1	1
PhysPhys	0	0	0

Table 3: Effingham age-based record pairs.

	Pairs	Sig	Sig. +ve	Sig. %	Sig. detrend	Sig. detrend +ve	Sig. detrend %
TOC	3	0	0	0	0	0	0
TN	3	0	0	0	0	0	0
CN	3	0	0	0	0	0	0
Carb	3	1	1	33	1	1	33
Opal	3	0	0	0	0	0	0
d15N	3	0	0	0	0	0	0
d13C	0	0	0	-	0	0	-
ForamAssemb	0	0	0	-	0	0	-
Productivity	3	0	0	0	0	0	0
BWO2	6	0	0	0	0	0	0
Quartz	0	0	0	-	0	0	-
SST	3	0	0	0	0	0	0
Al	0	0	0	-	0	0	-

Table 4: Effingham Fish-bgc and Fish-phys age-based record pairs.

	Pairs	Significant	Sig. detrend
FishFish	15	3	3
FishBgc	30	2	2
BgcBgc	10	5	3
FishPhys	6	0	0
PhysBgc	5	4	5
PhysPhys	0	0	0

Table 5: Guaymas age-based record pairs.

	Pairs	Sig	Sig. +ve	Sig. %	Sig. detrend	Sig. detrend +ve	Sig. detrend %
TOC	6	0	0	0	0	0	0
TN	6	1	0	17	1	0	17
CN	6	1	1	17	1	1	17
Carb	0	0	0	-	0	0	-
Opal	0	0	0	-	0	0	-
d15N	0	0	0	-	0	0	-
d13C	0	0	0	-	0	0	-
ForamAssemb	0	0	0	-	0	0	-
Productivity	0	0	0	-	0	0	-
BWO2	12	0	0	0	0	0	0
Quartz	0	0	0	-	0	0	-
SST	0	0	0	-	0	0	-
Al	6	0	0	0	0	0	0

Table 6: Guaymas Fish-bgc and Fish-phys age-based record pairs.

	Pairs	Significant	Sig. detrend
FishFish	3	1	1
FishBgc	15	5	5
BgcBgc	10	7	2
FishPhys	6	2	1
PhysBgc	10	2	0
PhysPhys	1	0	0

Table 7: Mejillones age-based record pairs.

	Pairs	Sig	Sig. +ve	Sig. %	Sig. detrend	Sig. detrend +ve	Sig. detrend %
TOC	9	3	3	33	3	3	33
TN	0	0	0	-	0	0	-
CN	0	0	0	-	0	0	-
Carb	0	0	0	-	0	0	-
Opal	0	0	0	-	0	0	-
d15N	3	2	2	67	2	2	67
d13C	0	0	0	-	0	0	-
ForamAssemb	0	0	0	-	0	0	-
Productivity	0	0	0	-	0	0	-
BWO2	3	0	0	0	0	0	0
Quartz	3	1	1	33	1	1	33
SST	3	1	0	33	0	0	0
Al	0	0	0	-	0	0	-

Table 8: Mejillones Fish-bgc and Fish-phys age-based record pairs.

	Pairs	Significant	Sig. detrend
FishFish	105	34	32
FishBgc	133	15	13
BgcBgc	25	13	6
FishPhys	30	1	2
PhysBgc	16	5	3
PhysPhys	1	0	0

Table 9: Pisco age-based record pairs.

	Pairs	Sig	Sig. +ve	Sig. %	Sig. detrend	Sig. detrend +ve	Sig. detrend %
TOC	45	6	4	13	5	1	11
TN	15	0	0	0	0	0	0
CN	0	0	0	-	0	0	-
Carb	15	1	1	7	2	1	13
Opal	15	6	1	40	3	1	20
d15N	30	0	0	0	0	0	0
d13C	0	0	0	-	0	0	-
ForamAssemb	0	0	0	-	0	0	-
Productivity	0	0	0	-	0	0	-
BWO2	15	2	2	13	3	2	20
Quartz	15	1	1	7	3	1	20
SST	15	0	0	0	0	0	0
Al	0	0	0	-	0	0	-

Table 10: Pisco Fish-bgc and Fish-phys age-based record pairs.

	Pairs	Significant	Sig. detrend
FishFish	0	0	0
FishBgc	10	8	1
BgcBgc	45	35	15
FishPhys	1	1	0
PhysBgc	10	9	6
PhysPhys	0	0	0

Table 11: Saanich age-based record pairs.

	Pairs	Sig	Sig. +ve	Sig. %	Sig. detrend	Sig. detrend +ve	Sig. detrend %
TOC	2	2	0	100	0	0	0
TN	1	1	0	100	0	0	0
CN	1	1	1	100	0	0	0
Carb	1	0	0	0	0	0	0
Opal	1	1	0	100	0	0	0
d15N	1	0	0	0	0	0	0
d13C	1	1	0	100	1	1	100
ForamAssemb	0	0	0	-	0	0	-
Productivity	0	0	0	-	0	0	-
BWO2	2	2	0	100	0	0	0
Quartz	0	0	0	-	0	0	-
SST	0	0	0	-	0	0	-
Al	1	1	1	100	0	0	0

Table 12: Saanich Fish-bgc and Fish-phys age-based record pairs.

	Pairs	Significant	Sig. detrend
FishFish	45	11	8
FishBgc	66	7	6
BgcBgc	68	45	41
FishPhys	6	0	0
PhysBgc	2	0	0
PhysPhys	0	0	0

Table 13: Santa Barbara age-based record pairs.

	Pairs	Sig	Sig. +ve	Sig. %	Sig. detrend	Sig. detrend +ve	Sig. detrend %
TOC	12	2	0	17	3	0	25
TN	0	0	0	-	0	0	-
CN	0	0	0	-	0	0	-
Carb	0	0	0	-	0	0	-
Opal	0	0	0	-	0	0	-
d15N	12	3	3	25	3	3	25
d13C	0	0	0	-	0	0	-
ForamAssemb	12	1	0	8	0	0	0
Productivity	6	1	1	17	0	0	0
BWO2	24	0	0	0	0	0	0
Quartz	0	0	0	-	0	0	-
SST	6	0	0	0	0	0	0
Al	0	0	0	-	0	0	-

Table 14: Santa Barbara Fish-bgc and Fish-phys age-based record pairs.

	Pairs	Significant	Sig. detrend
FishFish	6	1	2
FishBgc	4	1	1
BgcBgc	0	0	0
FishPhys	0	0	0
PhysBgc	0	0	0
PhysPhys	0	0	0

Table 15: Soledad age-based record pairs.

	Pairs	Sig	Sig. +ve	Sig. %	Sig. detrend	Sig. detrend +ve	Sig. detrend %
TOC	4	1	0	25	1	0	25
TN	0	0	0	-	0	0	-
CN	0	0	0	-	0	0	-
Carb	0	0	0	-	0	0	-
Opal	0	0	0	-	0	0	-
d15N	0	0	0	-	0	0	-
d13C	0	0	0	-	0	0	-
ForamAssemb	0	0	0	-	0	0	-
Productivity	0	0	0	-	0	0	-
BWO2	0	0	0	-	0	0	-
Quartz	0	0	0	-	0	0	-
SST	0	0	0	-	0	0	-
Al	0	0	0	-	0	0	-

Table 16: Soledad Fish-bgc and Fish-phys age-based record pairs.
