# Supplementary material for: Links between fish abundance and ocean biogeochemistry as recorded in marine sediments
Source: PLoS One. 2018 Aug 1;13(8):e0199420. doi: 10.1371/journal.pone.0199420 (PMC6070179; doi:10.1371/journal.pone.0199420)

S3 Correlation Coefficients The following figures are visual representations of the linear correlations between proxies at each site. The size and colour of each point is proportional to the Pearson r value. Only significant ($p < 0.05$) correlations are plotted, and those that do not remain significant after linear detrending are shown as ghosted points.

Figure 1: **Callao**

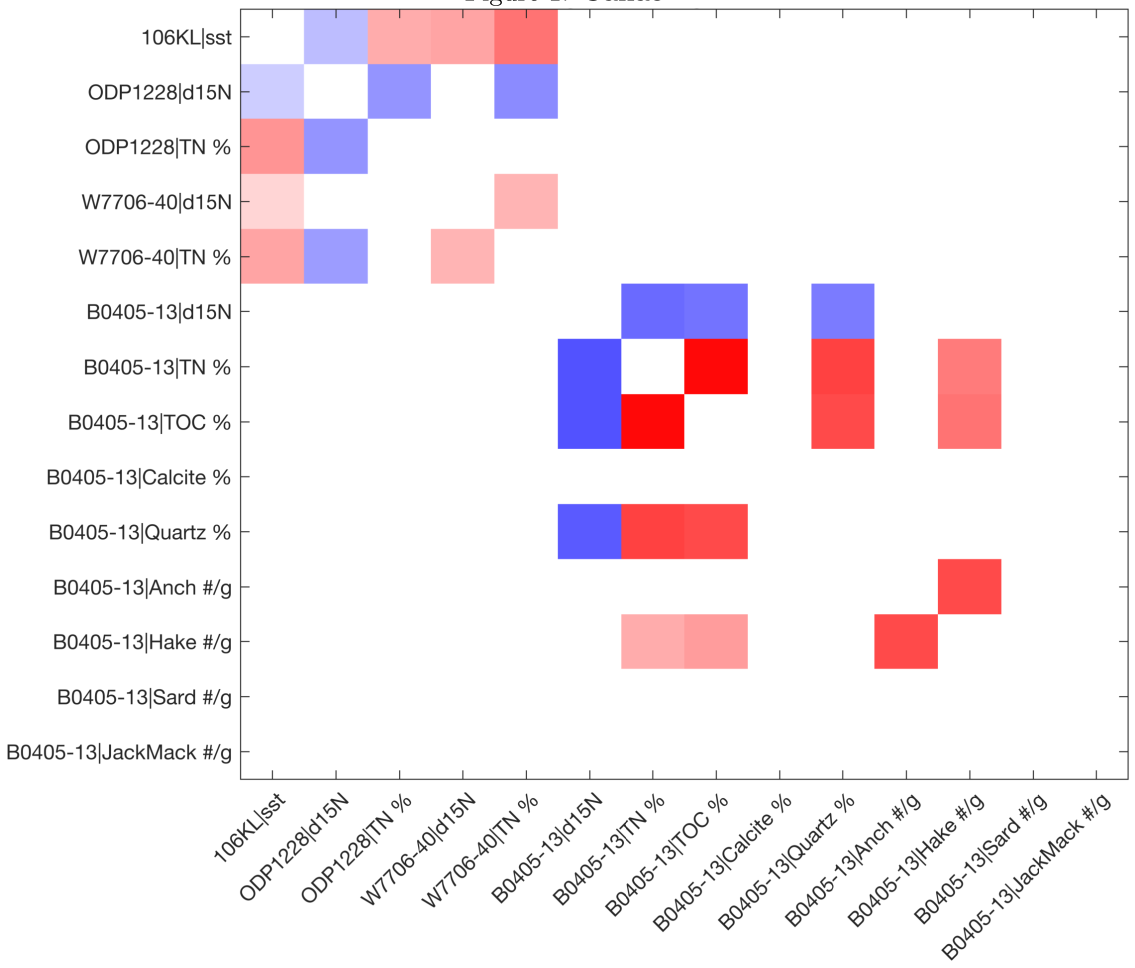

Figure 2: **Effingham Inlet**

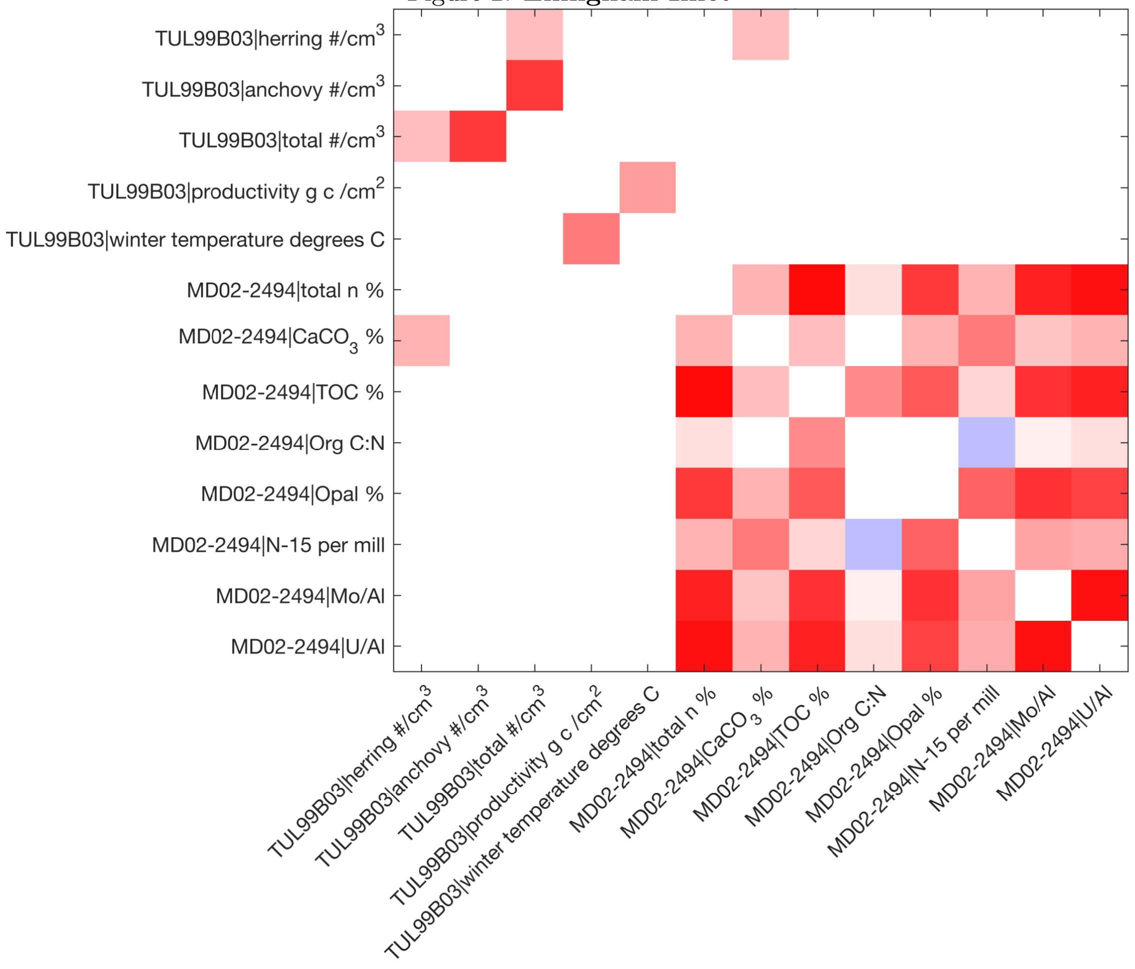

Figure 3: **Guaymas Basin**

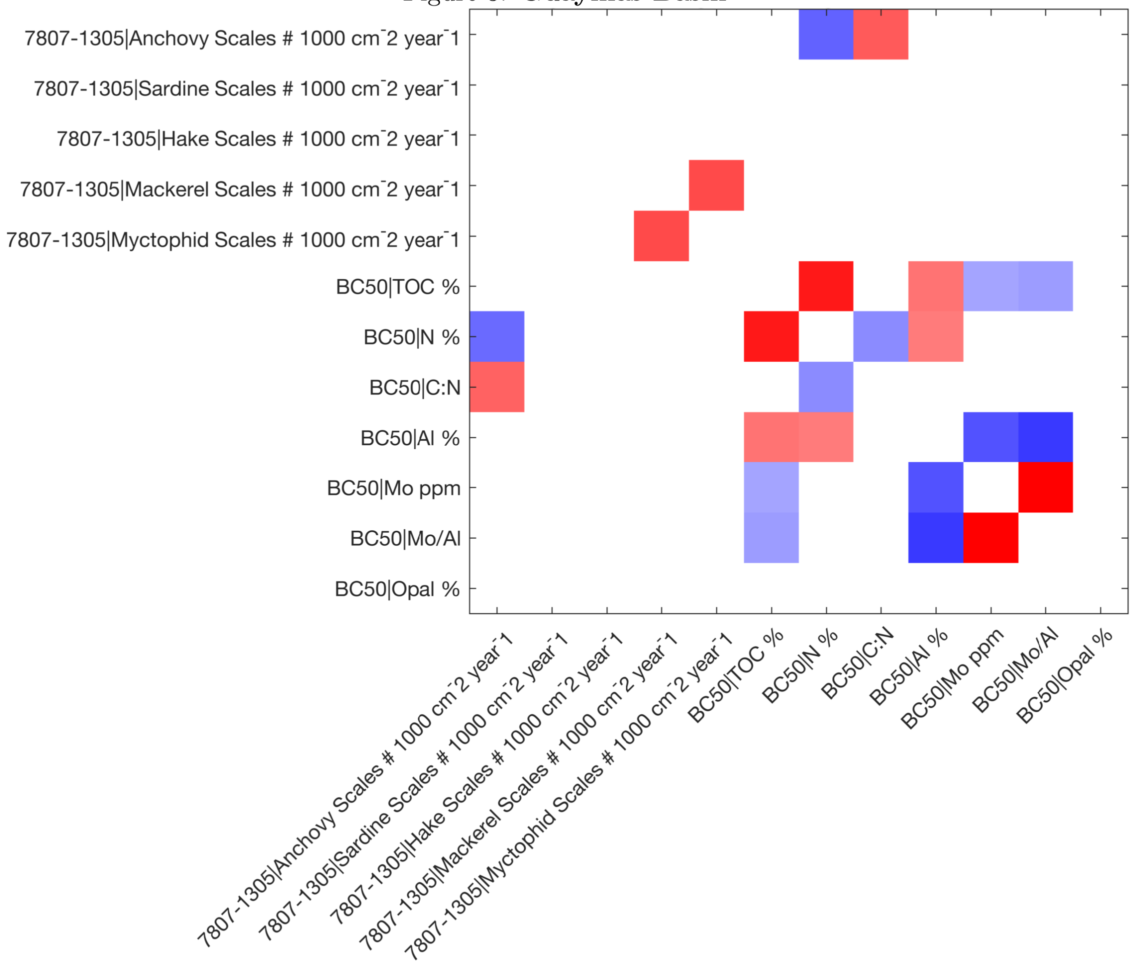

Figure 4: **Mejillones Bay**

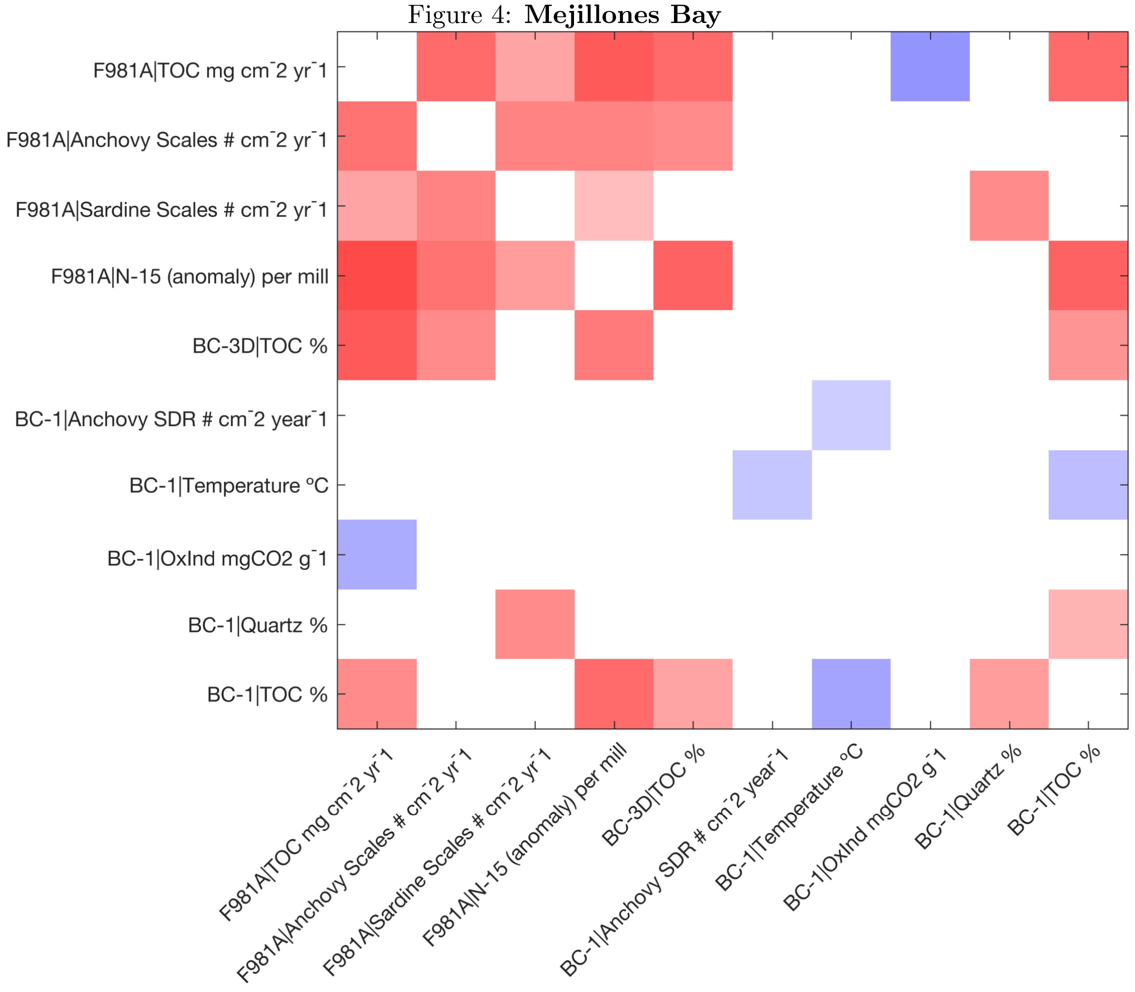

Figure 5: **Pisco**

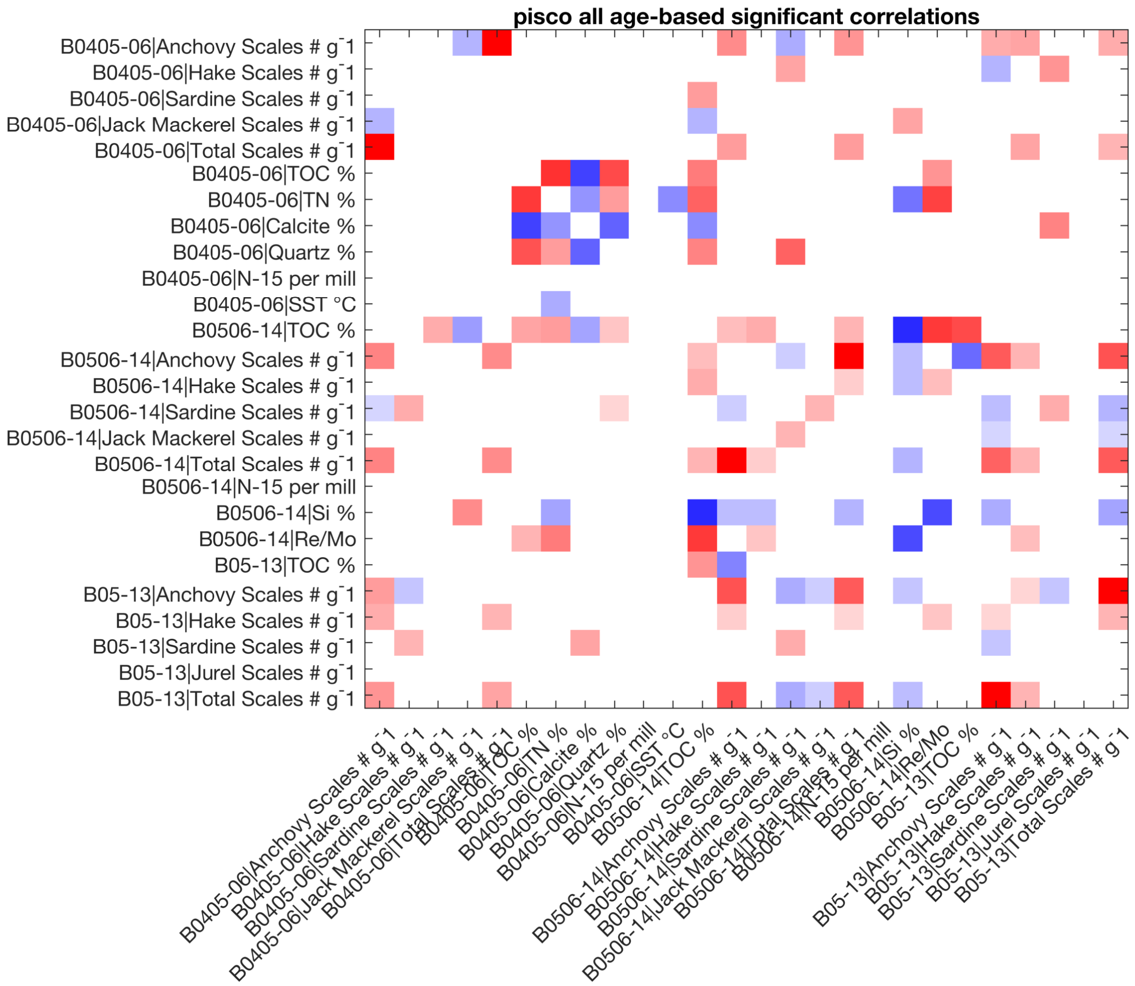

Figure 6: **Saanich Inlet**

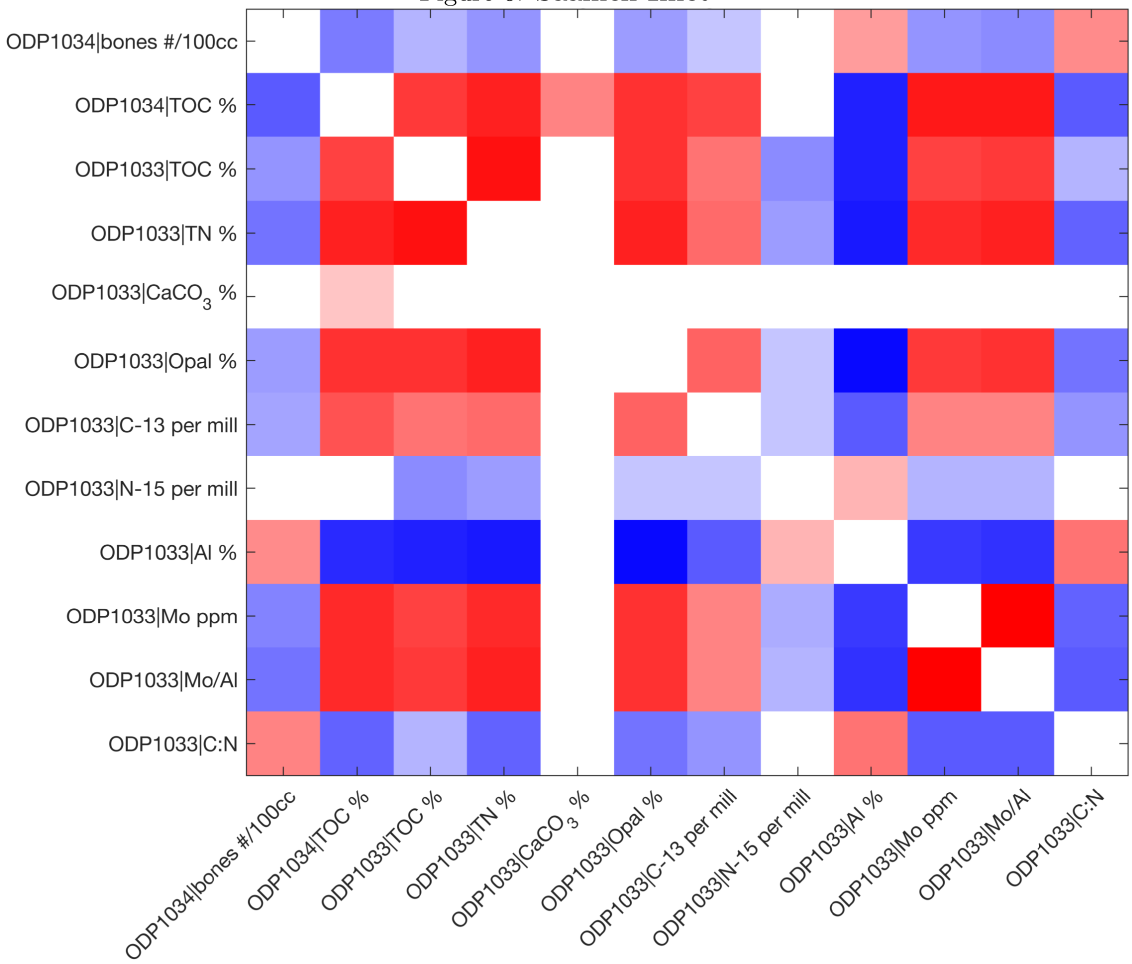

Figure 7: **Santa Barbara Basin**

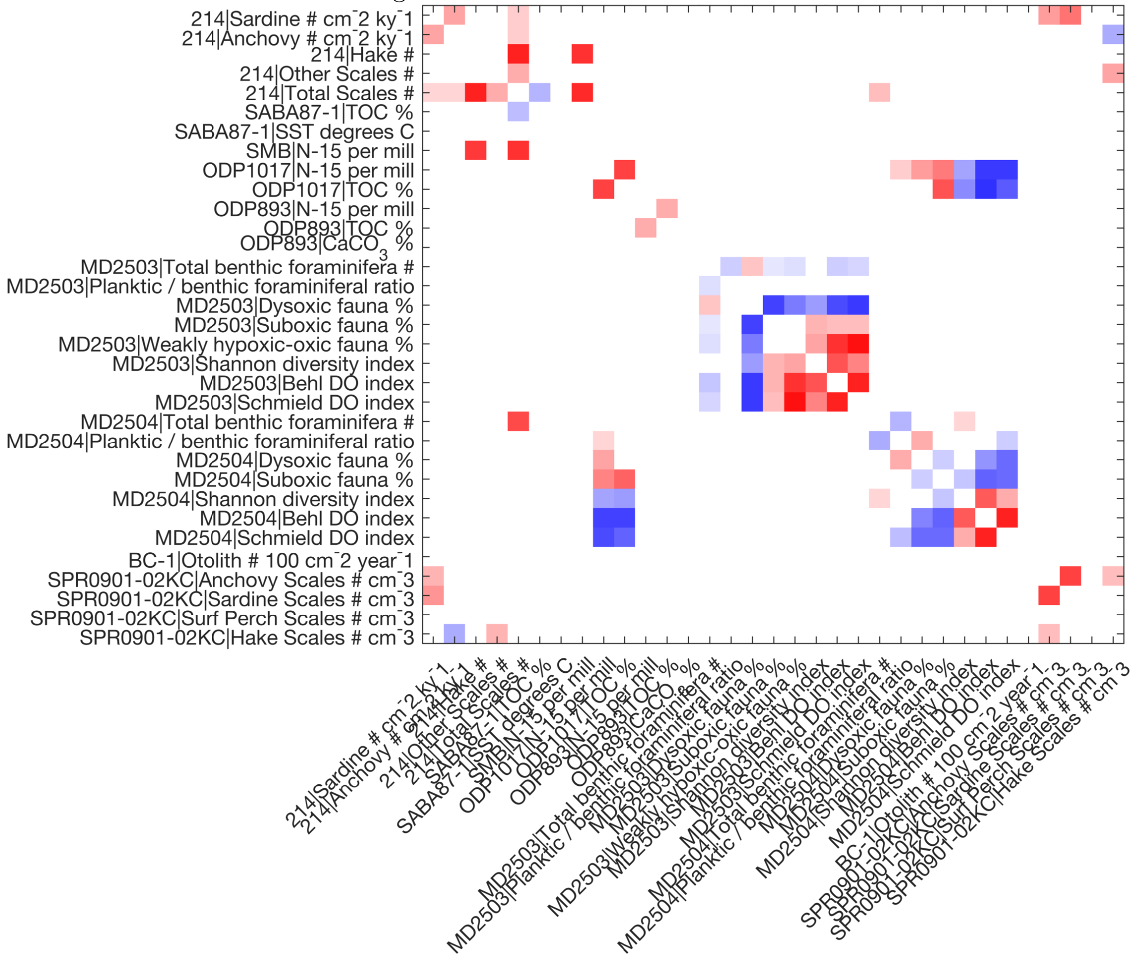

Figure 8: **Soledad Basin**

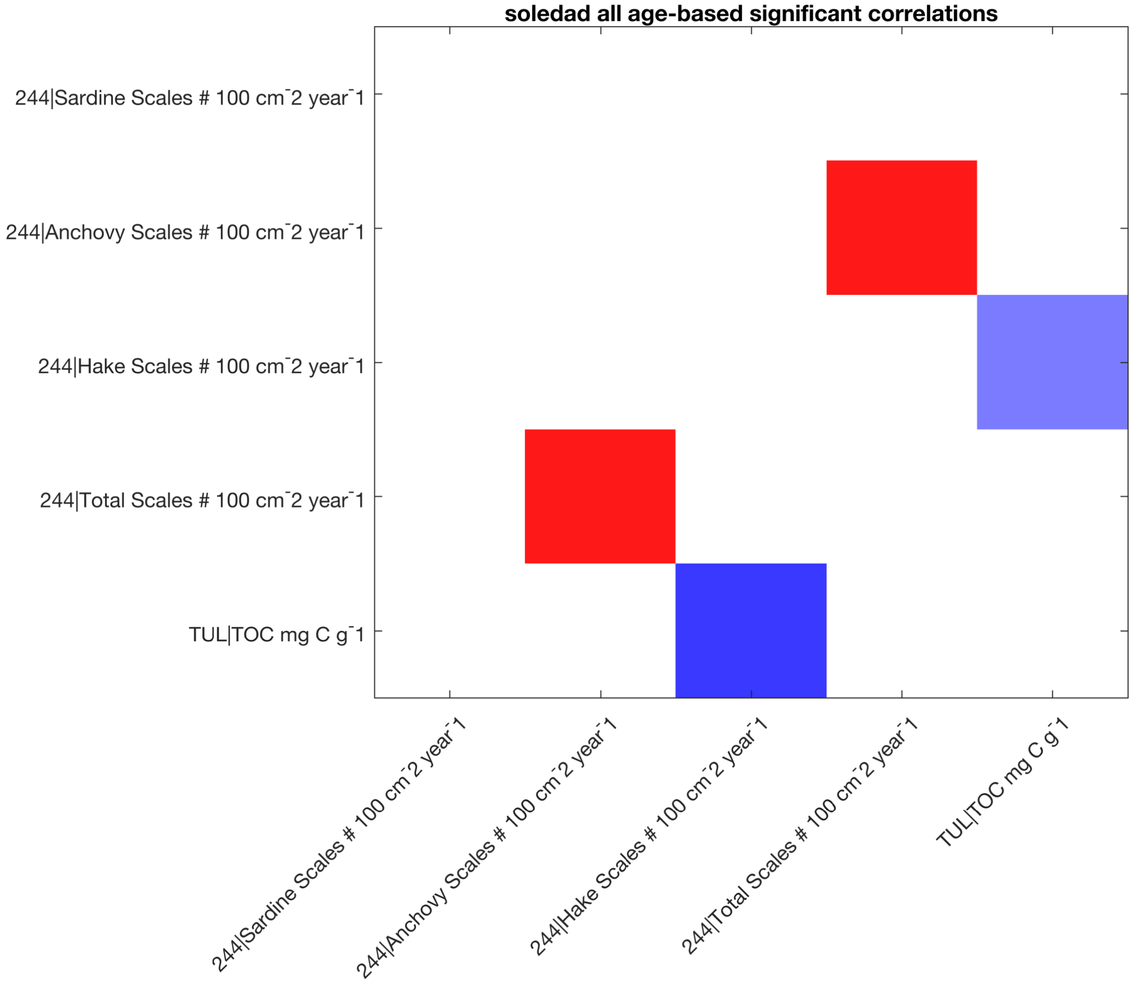

Supplement: S1 Fig — Matrix correlation plots of all proxy comparisons made at each site. (PDF) [file pone.0199420.s003.pdf]
