# Supplementary material for: Links between fish abundance and ocean biogeochemistry as recorded in marine sediments
Source: PLoS One. 2018 Aug 1;13(8):e0199420. doi: 10.1371/journal.pone.0199420 (PMC6070179; doi:10.1371/journal.pone.0199420)
Supplement: S1 Appendix — An outline of the preservation concerns at the Peruvian sites. (PDF) [file pone.0199420.s004.pdf]

S4 Appendix. Preservation Concerns

Preservation of TOC and fish scales can vary with the redox conditions of the water column and sediment. If there is a significant change in oxygenation over the time period examined, the preservation potential of both proxies could change dramatically, a critical consideration for evaluating correlations across such a transition. All four Peruvian cores examined display an abrupt shift at approximately 1820 towards more anchovy scales and higher TOC values [2]. This transition is seen most prominently in proxies of redox conditions (^{15}N and redox sensitive elements); an example record is shown in Figure 9, with the shaded region indicating higher oxygen conditions indicated by lower ^{15}N values.

The 1820 event has been interpreted as a shift in atmospheric circulation, driving increasing upwelling and bringing increased nutrient rich, oxygen depleted water to the surface. This postulated shift is consistent with reconstructed salinity, Sea Surface Temperature (SST), and precipitation proxies on both sides of the equatorial Pacific [2, 3]. Such a change could have had a significant impact on fish stocks and the greater abundance of anchovies may have impacted other proxies in the cores.

Lower scale counts and TOC concentrations before the 1820 transition are clear in Figure 9. [1] found that scales before this transition were significantly more degraded than those afterwards, which were very well preserved. The record of ^{15}N suggests considerable variability in oxygenation over this period, which approximately corresponds to peaks in scale counts and TOC, suggesting this correlation could be due to preservation of both proxies varying together. Caution should be taken interpreting this relationship, in particular due to possibilities of spurious correlations discussed in the main text; however, given the observations of better preservation by [1] and stability of the ^{15}N, only post-1820 data is used for all Peruvian cores in the main text.

Figure 9: Downcore records from Core B0405-06, Pisco, Peru. The shaded area indicates the period of higher oxygenation, supported by ^{15}N data.

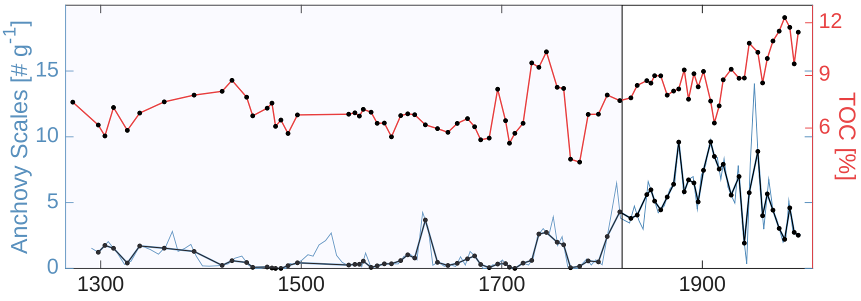

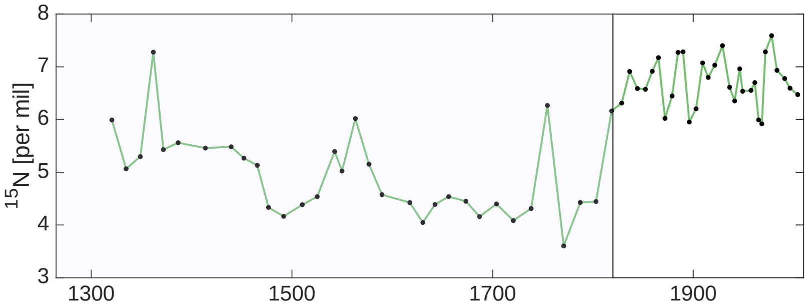

References

[1] Salvatteci R, Field D, Baumgartner T. Evaluating fish scale preservation in sediment records from the oxygen minimum zone off Peru. Paleobiology. 2012;38(1):52–78. doi:10.5061/dryad.7vg42.

[2] Gutiérrez D, Sifeddine A, Field DB, Ortlieb L, Vargas G, Chávez F, et al. Rapid reorganization in ocean biogeochemistry off Peru towards the end of the Little Ice Age. Biogeosciences Discussions. 2009;6(5):835–848. doi:10.5194/bgd-5-3919-2008.

[3] Sachs JP, Sachse D, Smittenberg RH, Zhang Z, Battisti DS, Golubic S. Southward movement of the Pacific intertropical convergence zone AD?1400–1850. Nature Geoscience. 2009;2(7):519–525. doi:10.1038/ngeo554.
