# Supplementary figures and images for: Links between fish abundance and ocean biogeochemistry as recorded in marine sediments
Source: PLoS One. 2018 Aug 1;13(8):e0199420. doi: 10.1371/journal.pone.0199420 (PMC6070179; doi:10.1371/journal.pone.0199420)

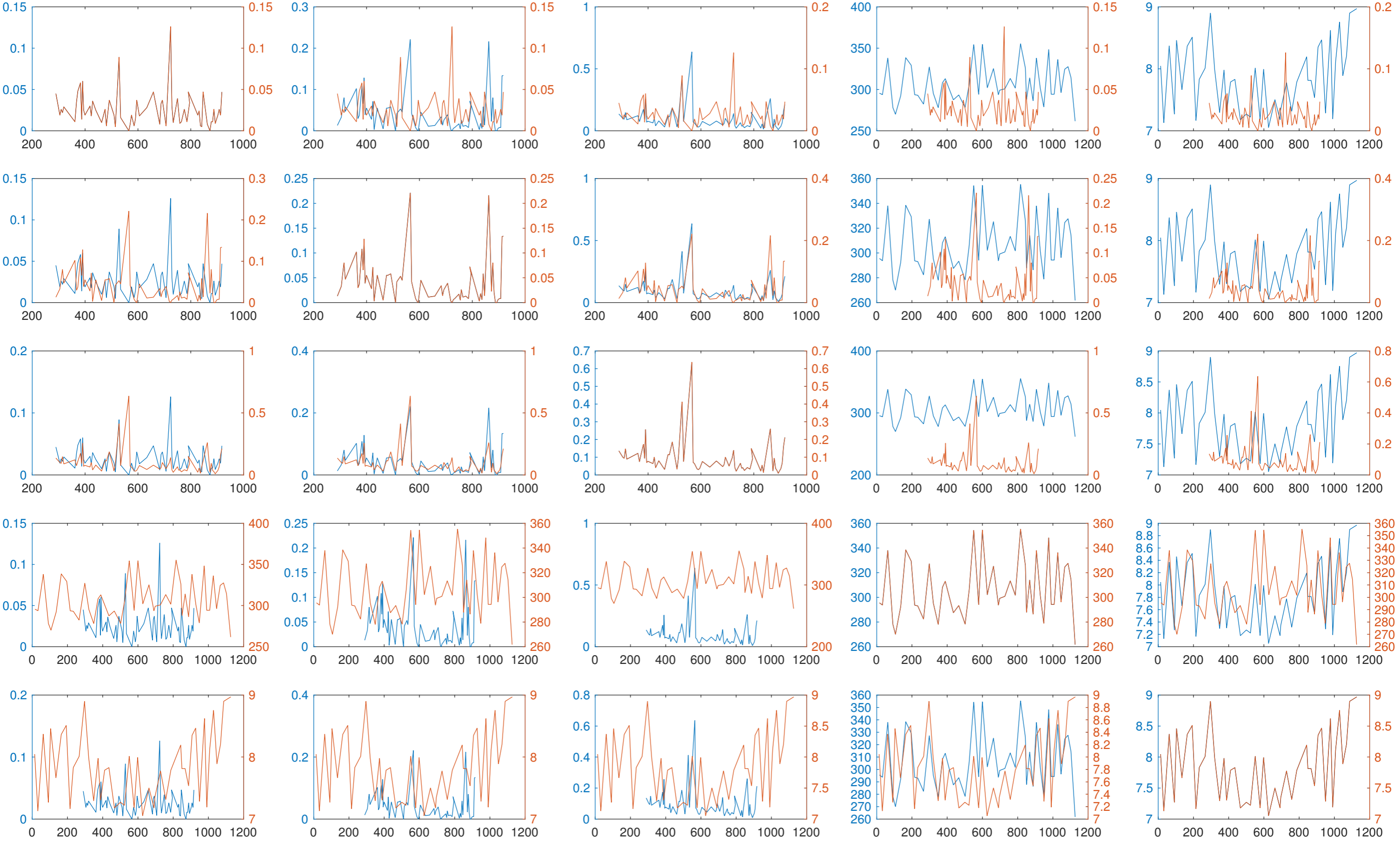

Supplement: S2 File — A zipped archive of all down-core plots and correlation tables used in this paper. (ZIP) [file pone.0199420.s006.zip › Downcore Plots and Correlation Tables/effingham/figures/TUL99B03.depth.curves.pdf]

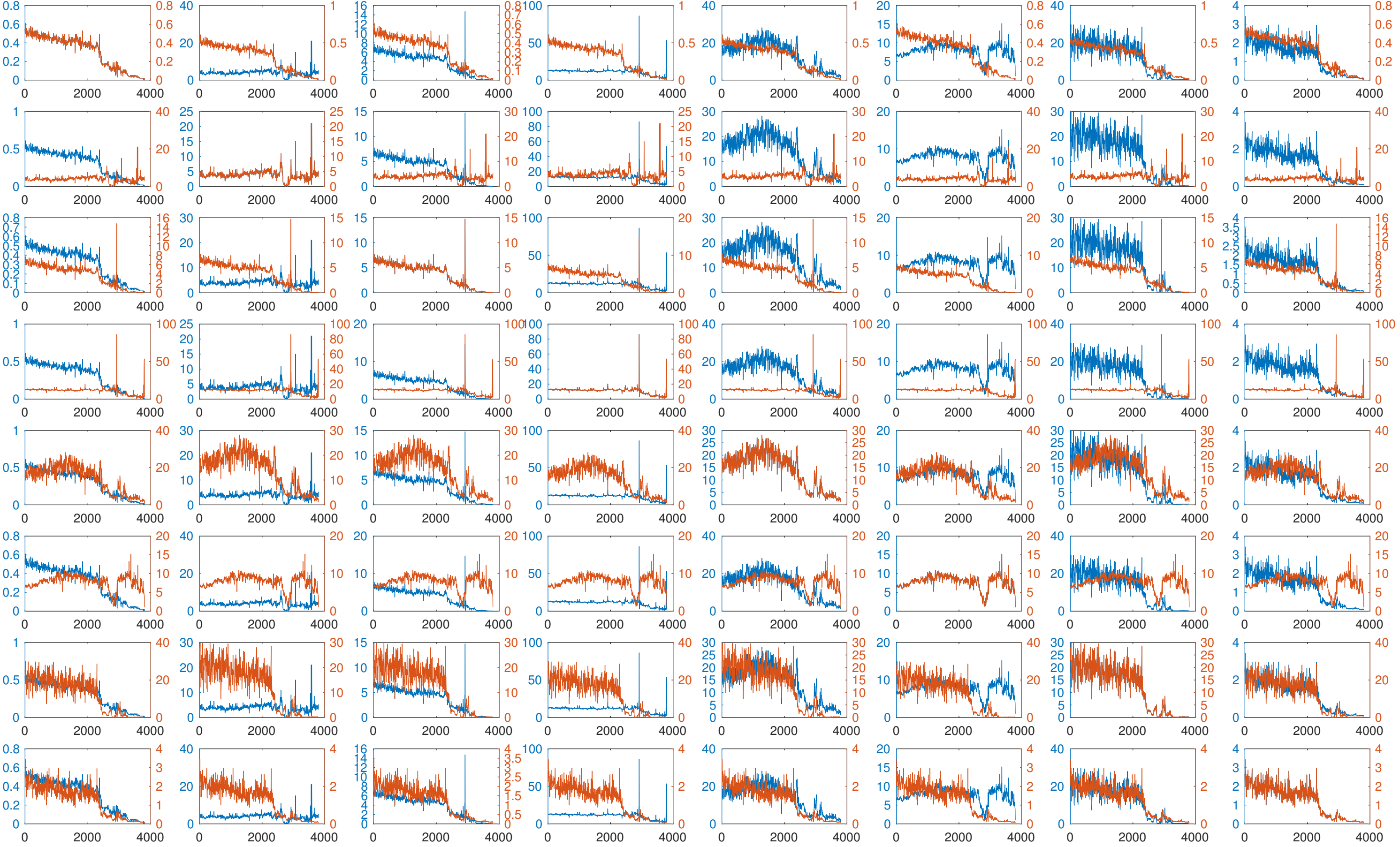

Supplement: S2 File — A zipped archive of all down-core plots and correlation tables used in this paper. (ZIP) [file pone.0199420.s006.zip › Downcore Plots and Correlation Tables/effingham/figures/MD02-2494.depth.curves.pdf]

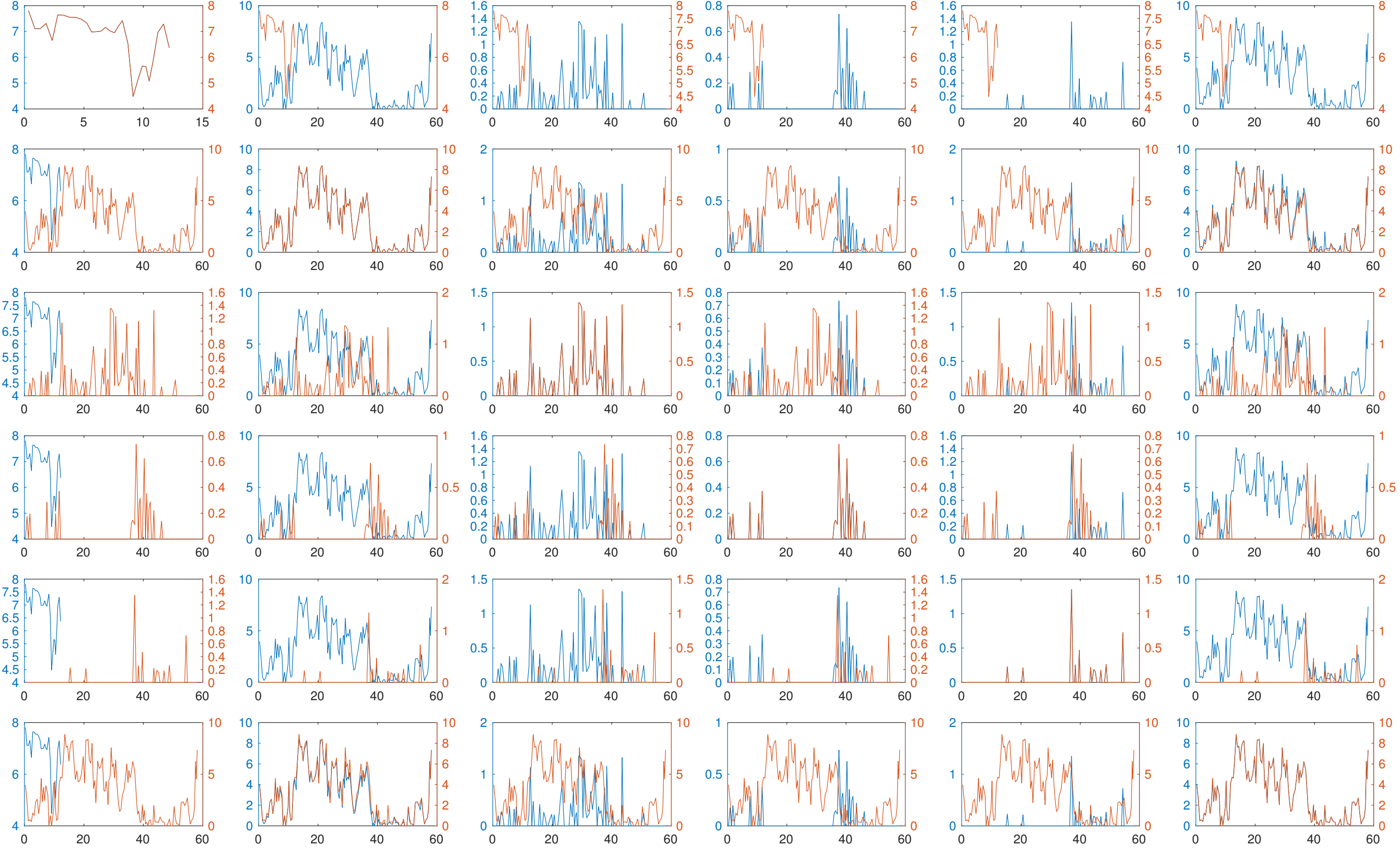

Supplement: S2 File — A zipped archive of all down-core plots and correlation tables used in this paper. (ZIP) [file pone.0199420.s006.zip › Downcore Plots and Correlation Tables/pisco/figures/B05-13.depth.curves.pdf]

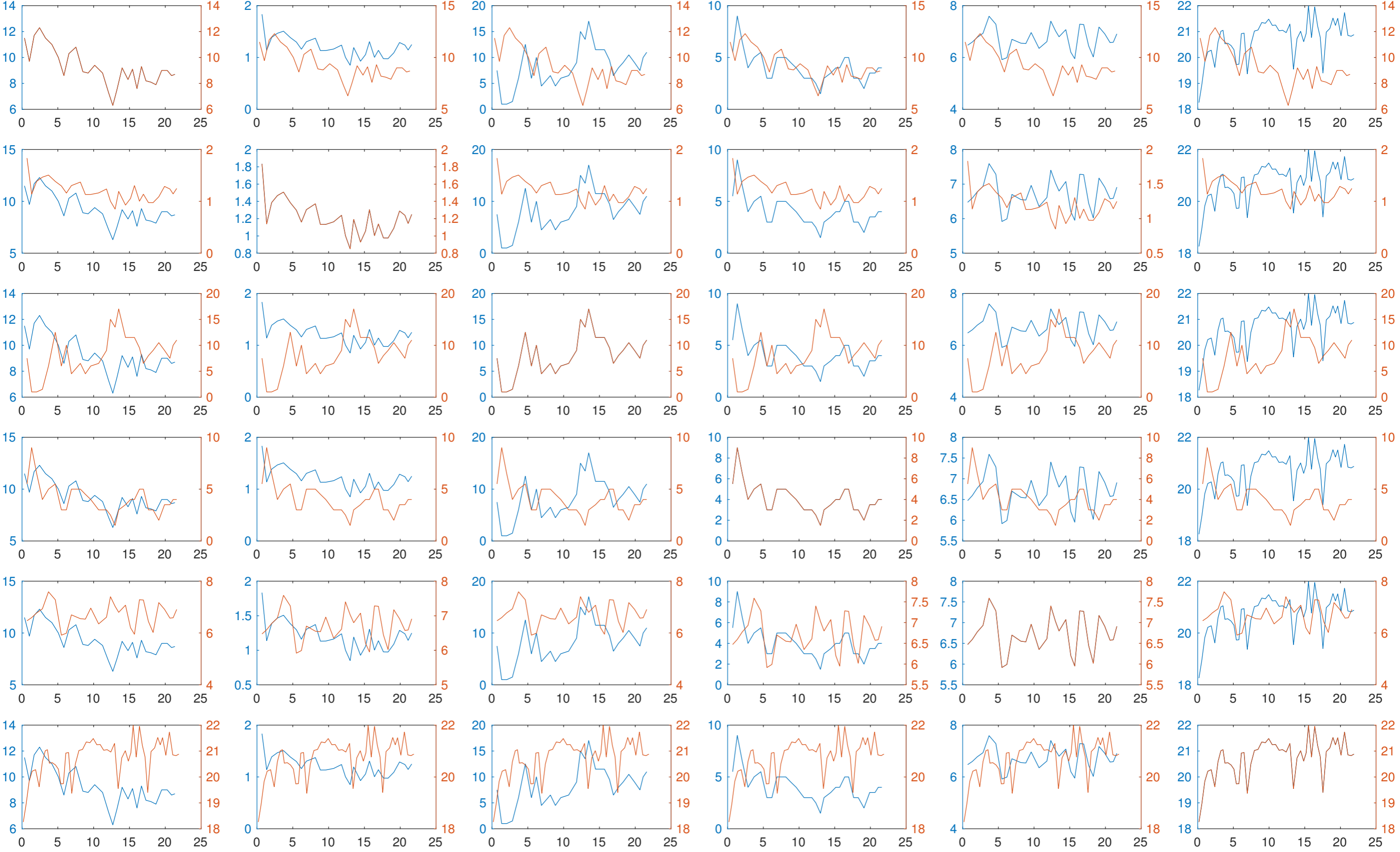

Supplement: S2 File — A zipped archive of all down-core plots and correlation tables used in this paper. (ZIP) [file pone.0199420.s006.zip › Downcore Plots and Correlation Tables/pisco/figures/B0405-06.depth.curves.pdf]

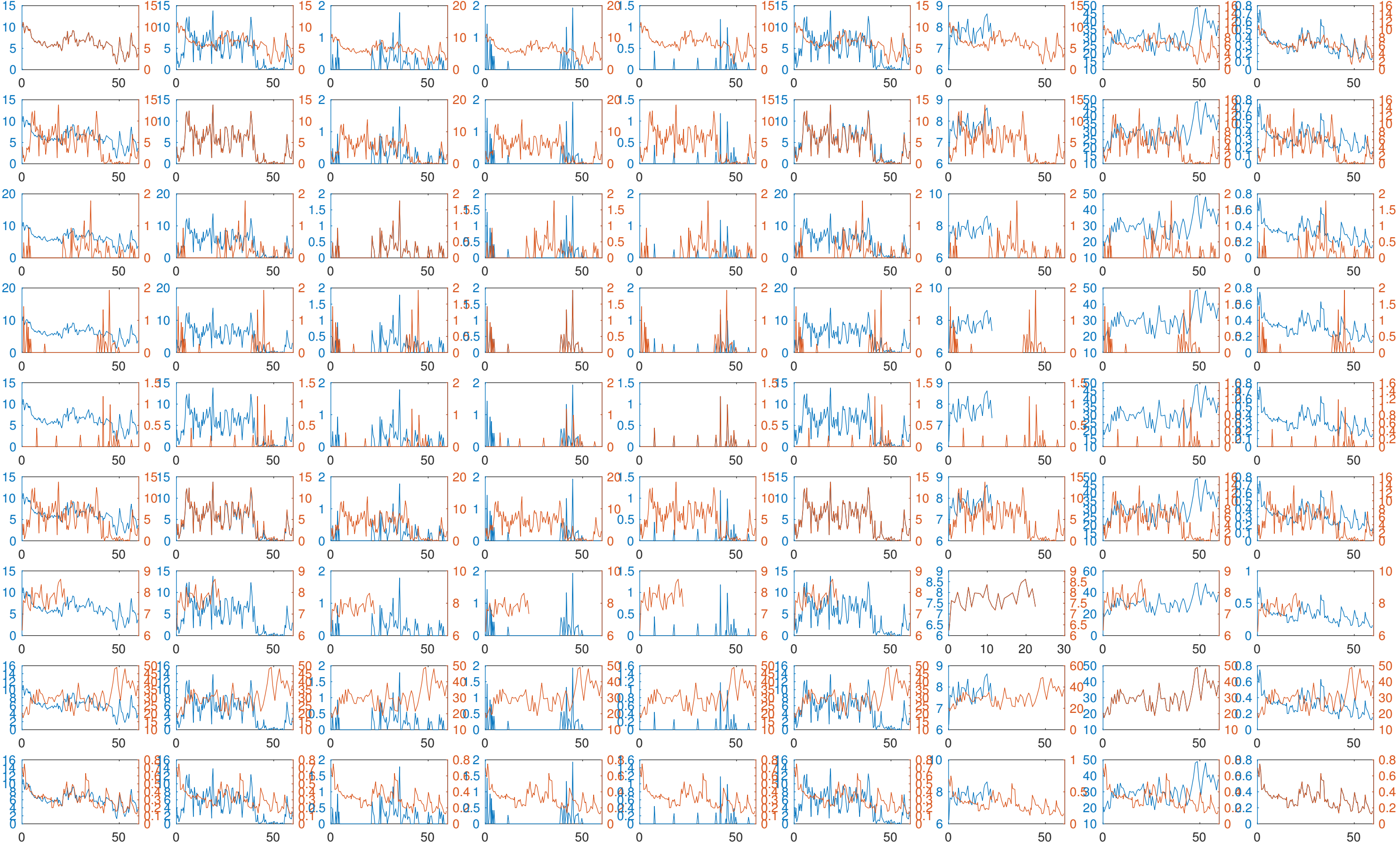

Supplement: S2 File — A zipped archive of all down-core plots and correlation tables used in this paper. (ZIP) [file pone.0199420.s006.zip › Downcore Plots and Correlation Tables/pisco/figures/B0506-14.depth.curves.pdf]

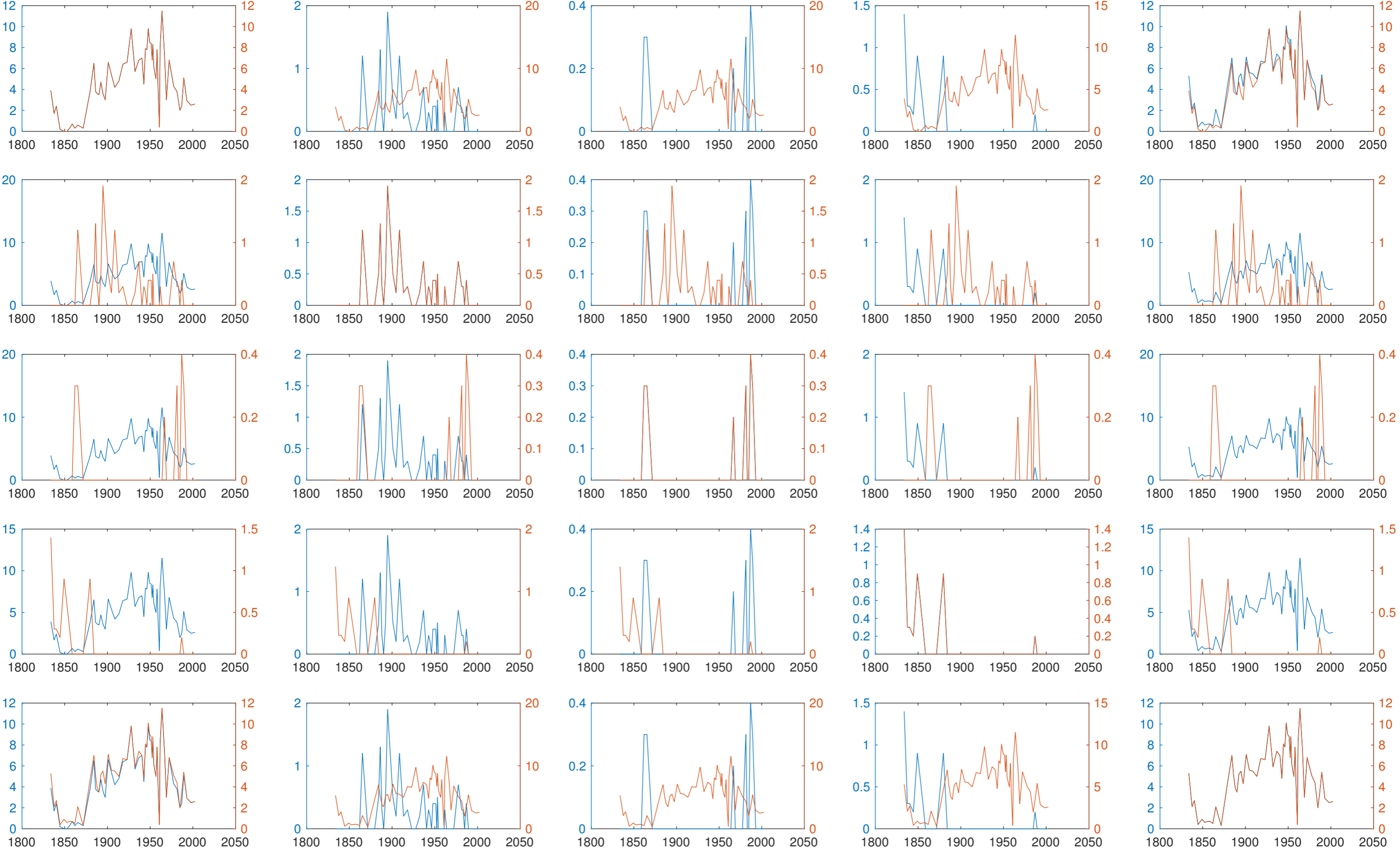

Supplement: S2 File — A zipped archive of all down-core plots and correlation tables used in this paper. (ZIP) [file pone.0199420.s006.zip › Downcore Plots and Correlation Tables/pisco/figures/B0405-06.year.curves.pdf]

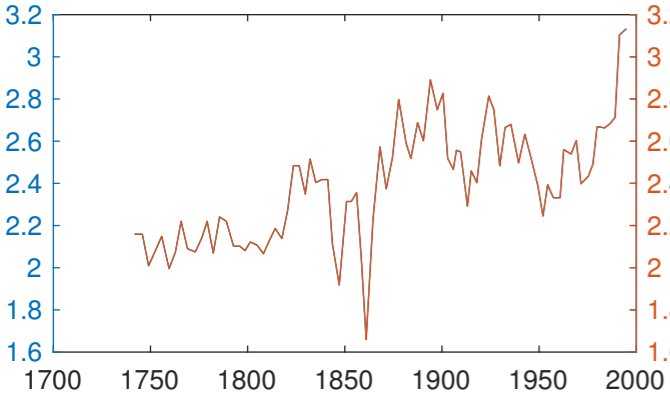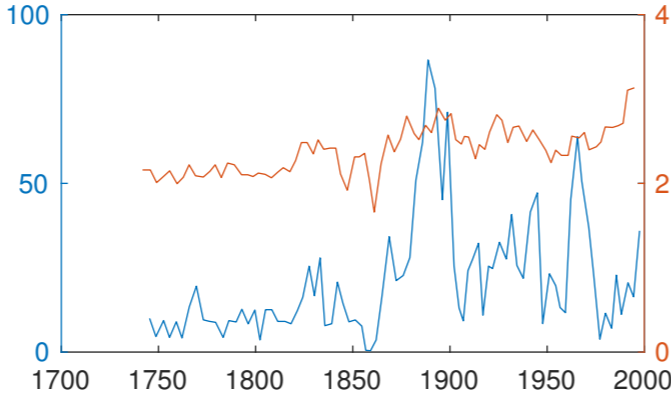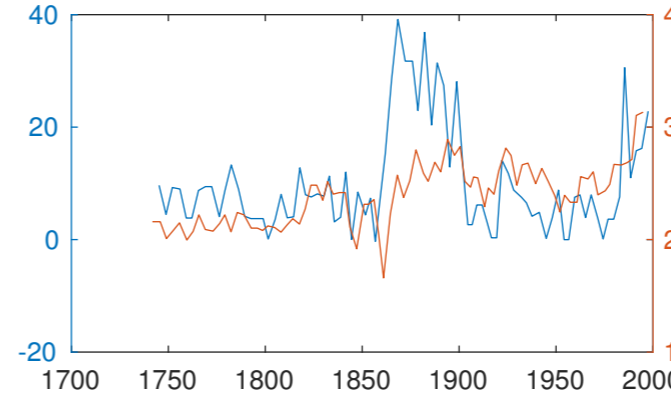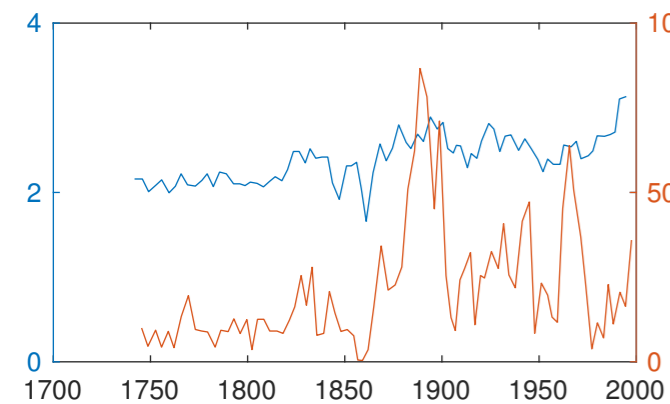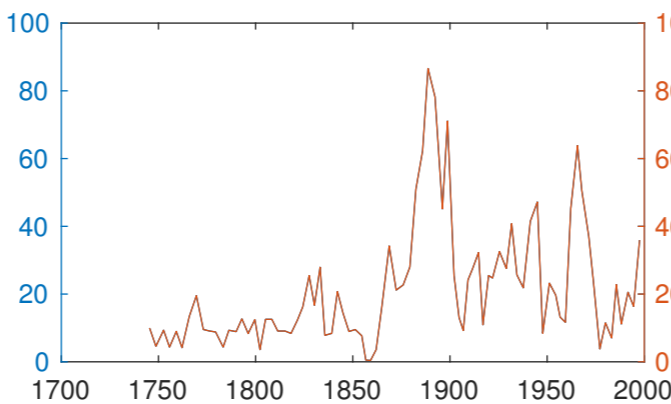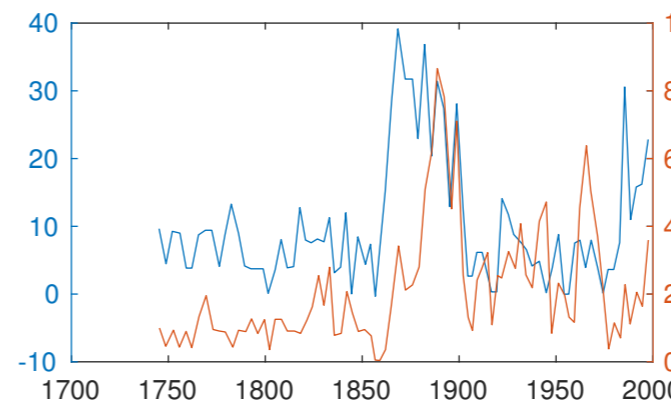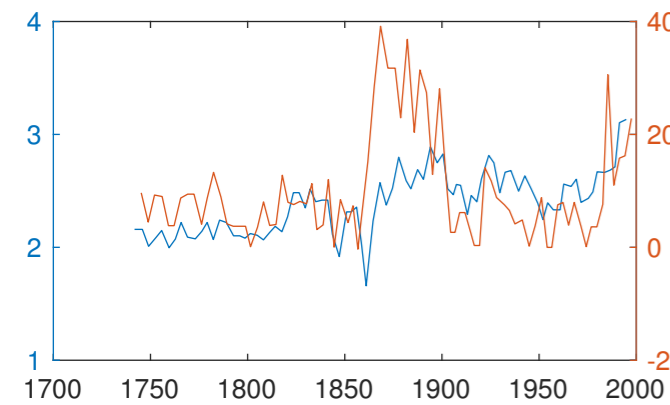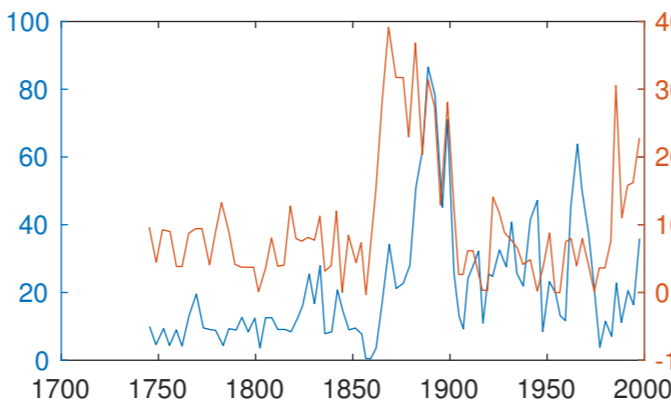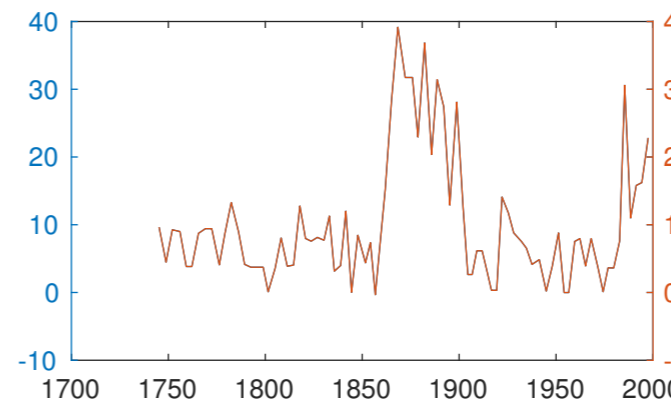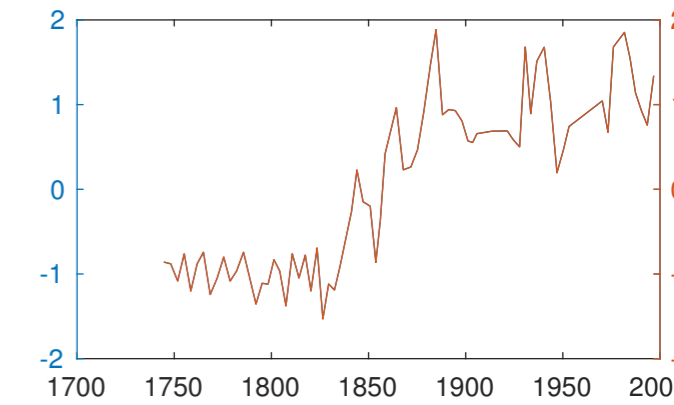

Supplement: S2 File — A zipped archive of all down-core plots and correlation tables used in this paper. (ZIP) [file pone.0199420.s006.zip › Downcore Plots and Correlation Tables/mejillones/figures/F981A.year.curves.pdf]

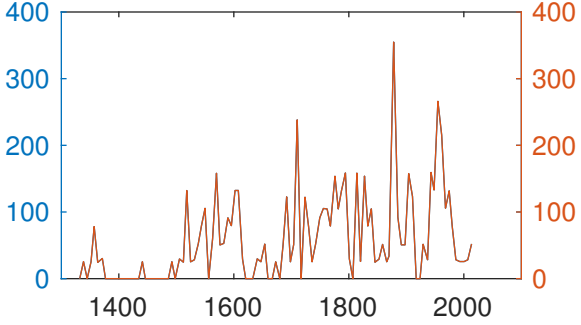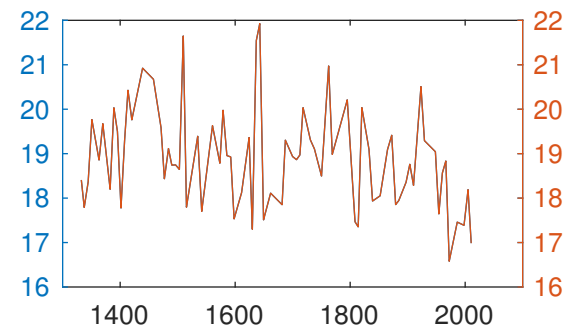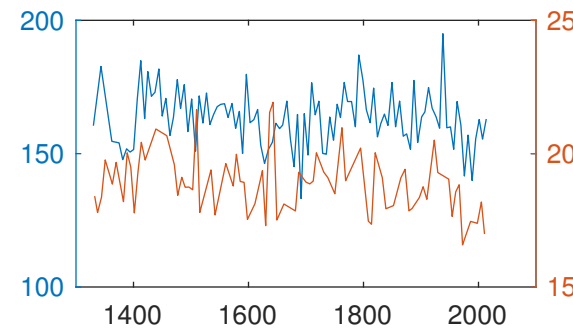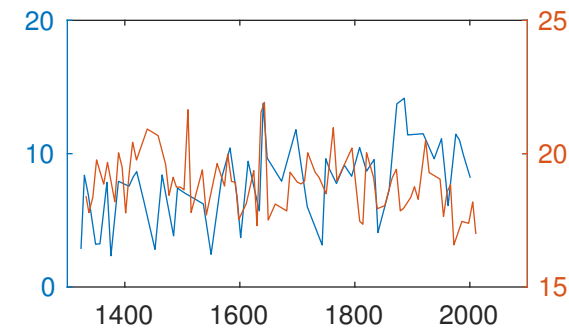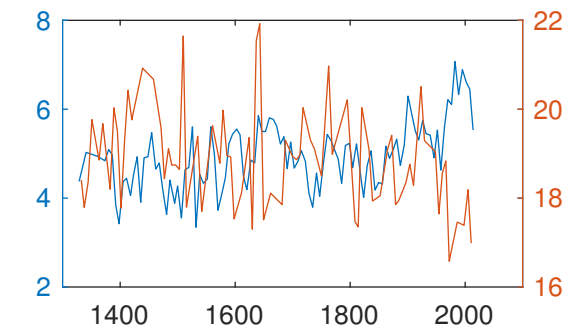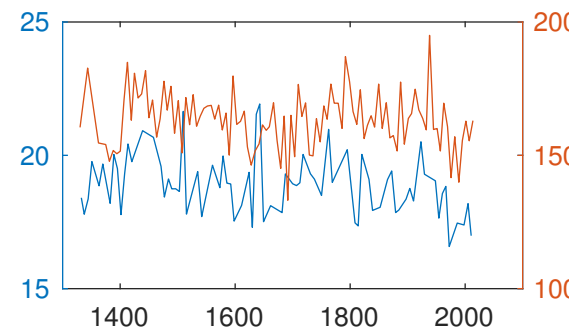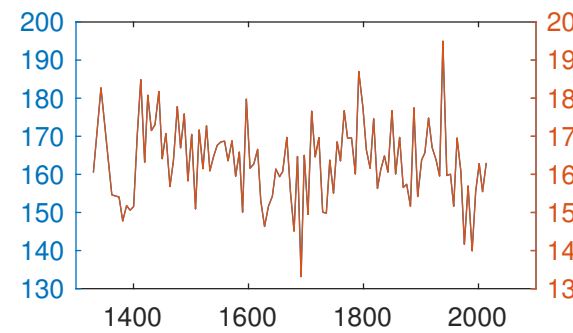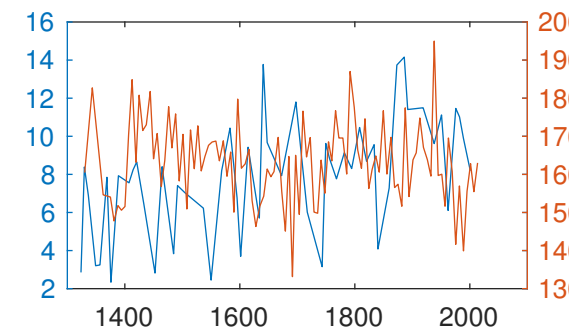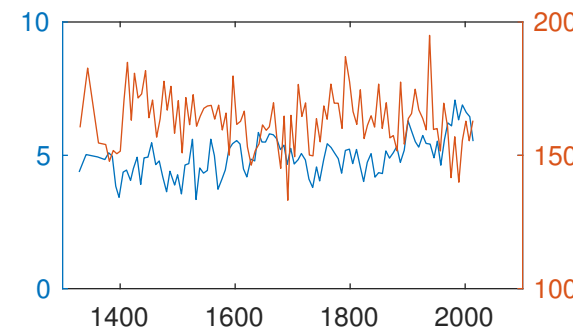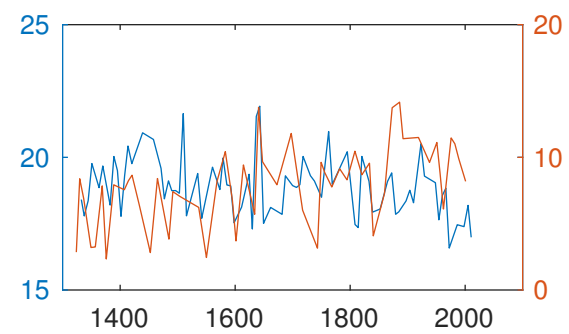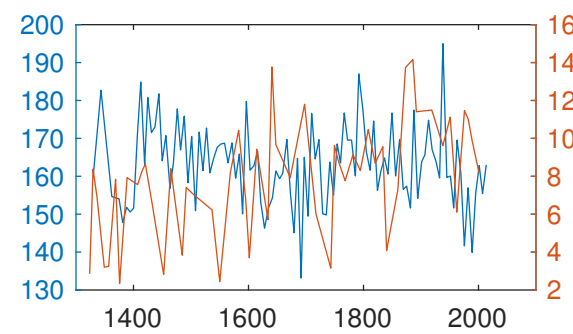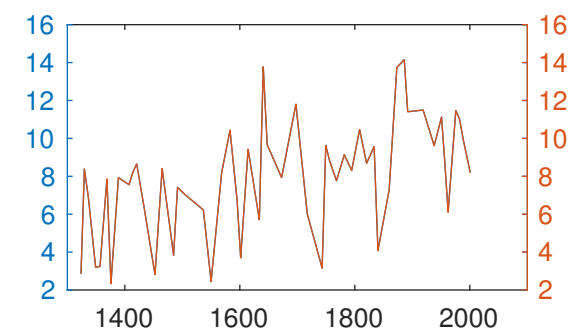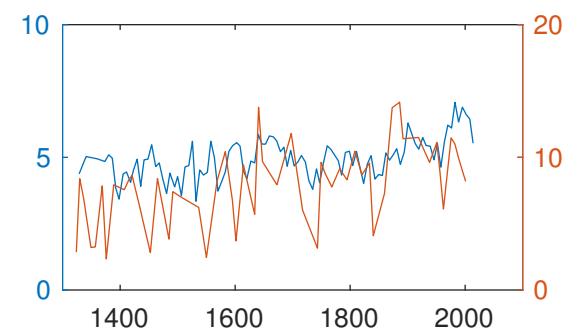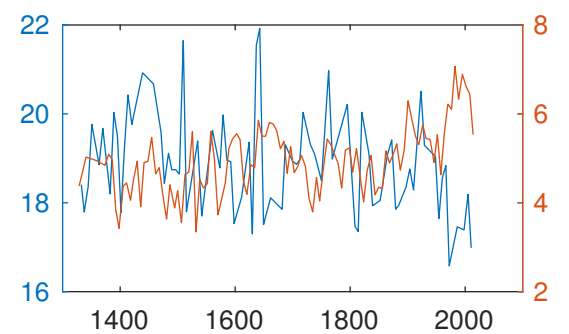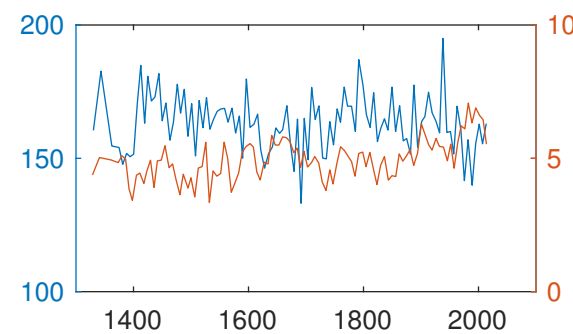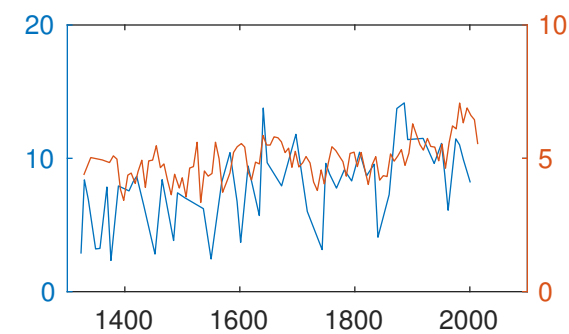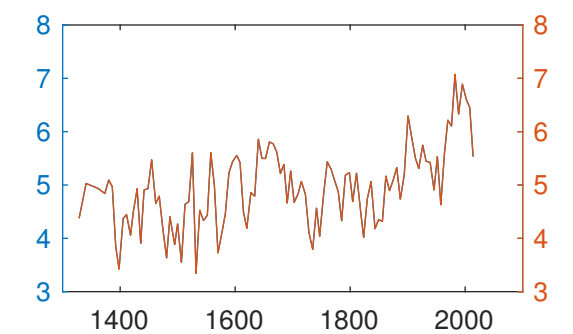

Supplement: S2 File — A zipped archive of all down-core plots and correlation tables used in this paper. (ZIP) [file pone.0199420.s006.zip › Downcore Plots and Correlation Tables/mejillones/figures/BC-1.year.curves.pdf]

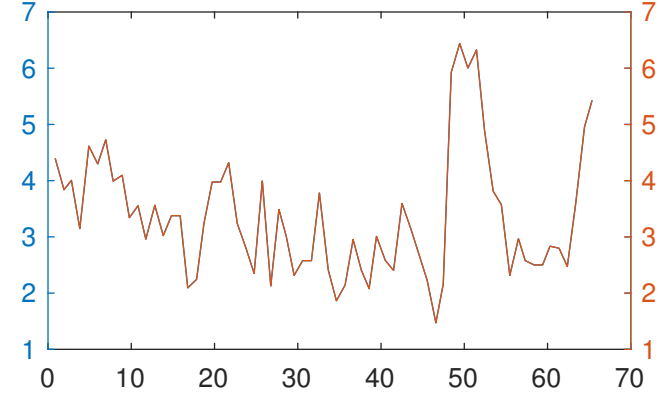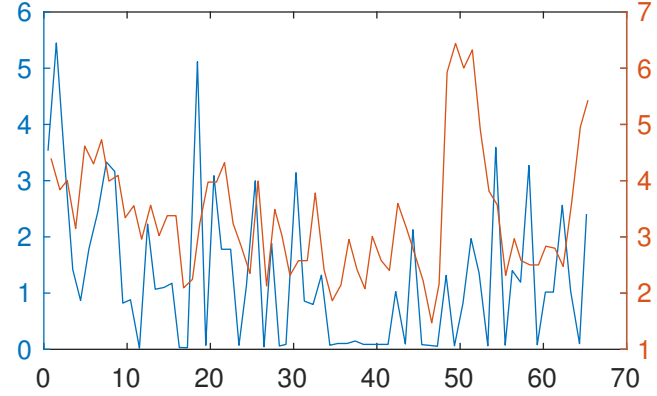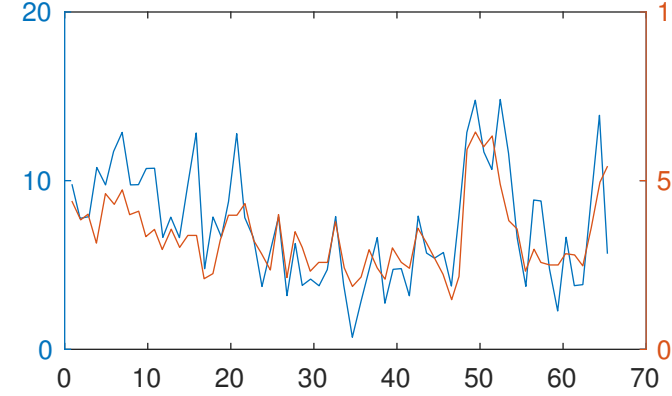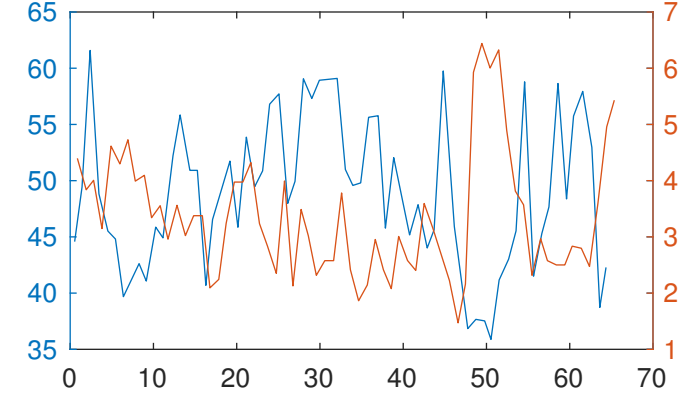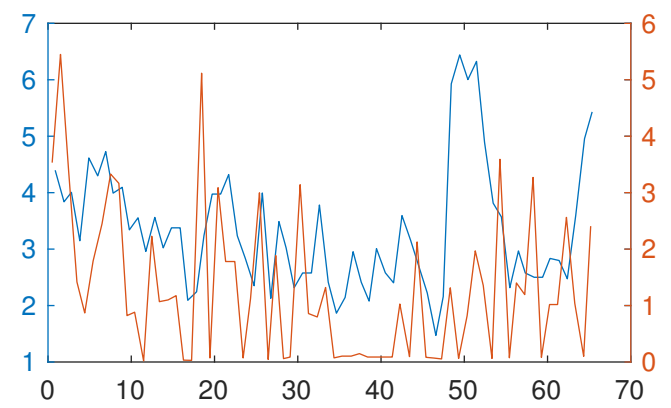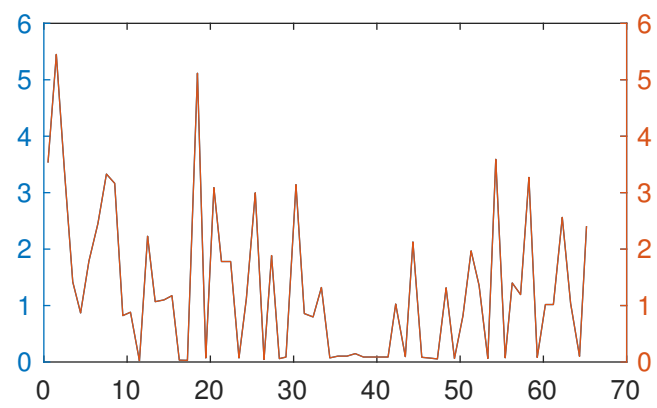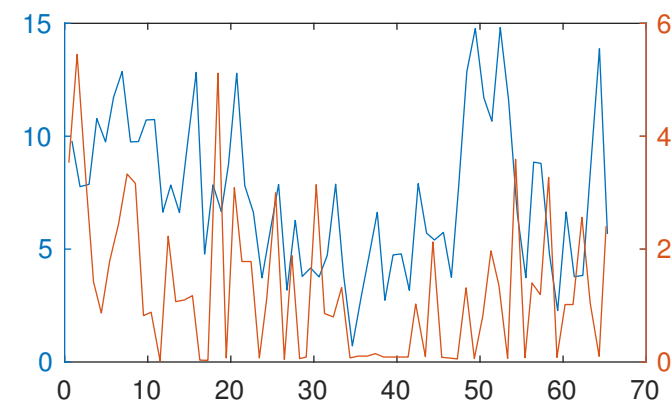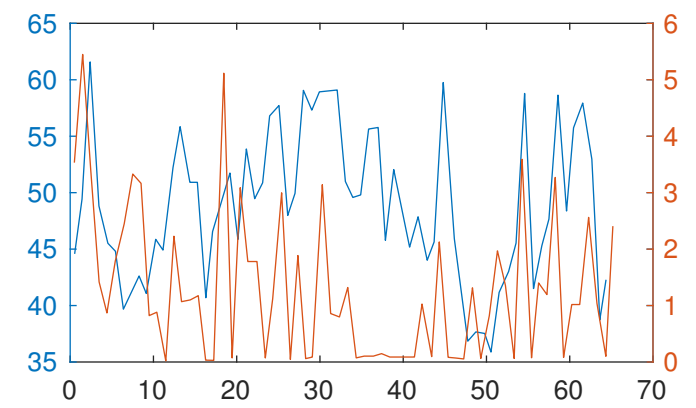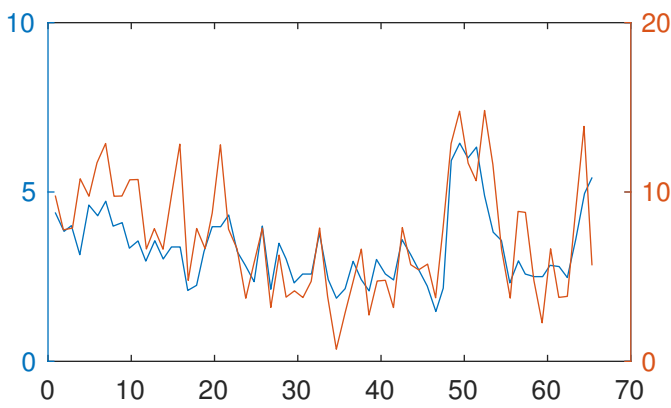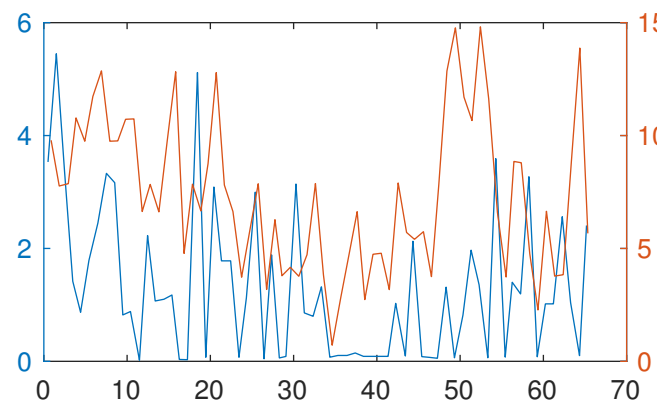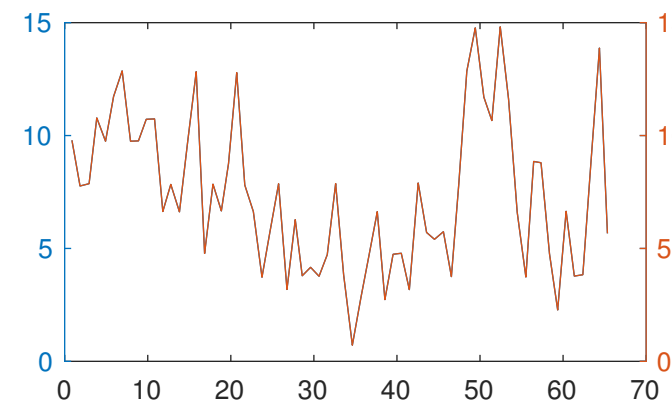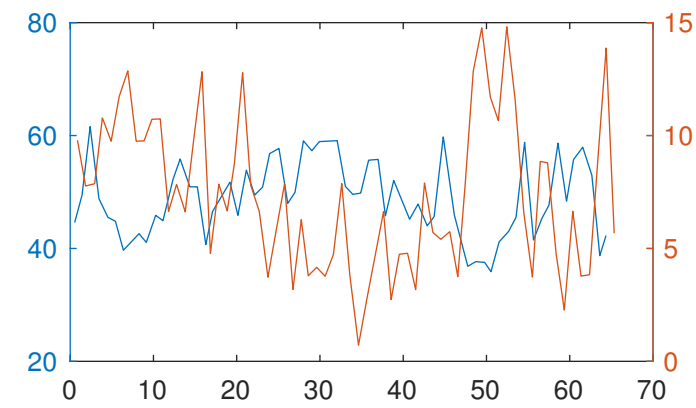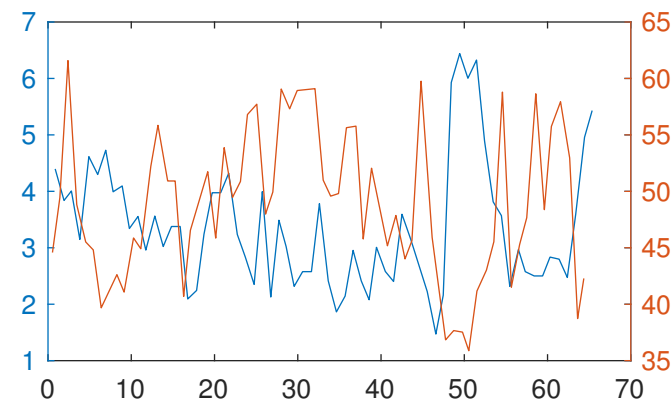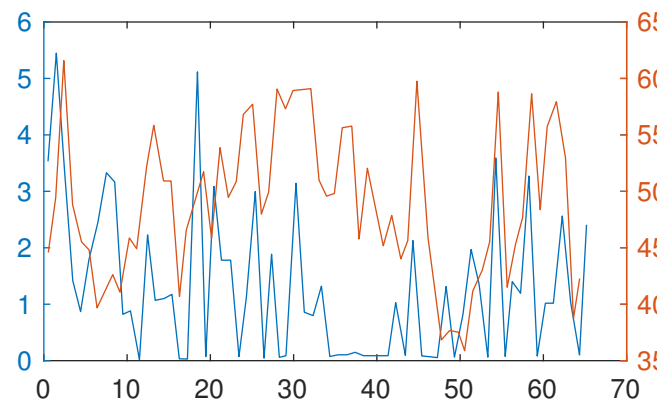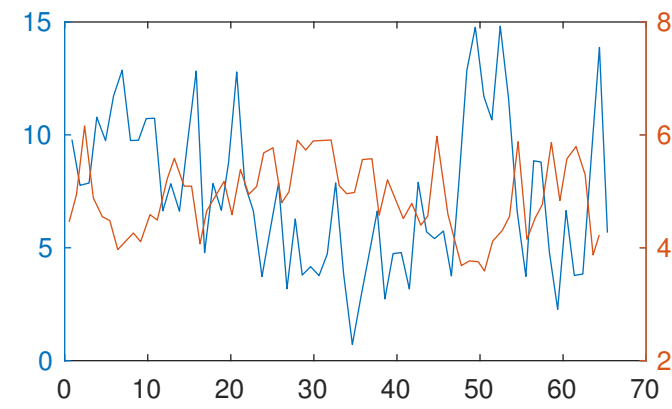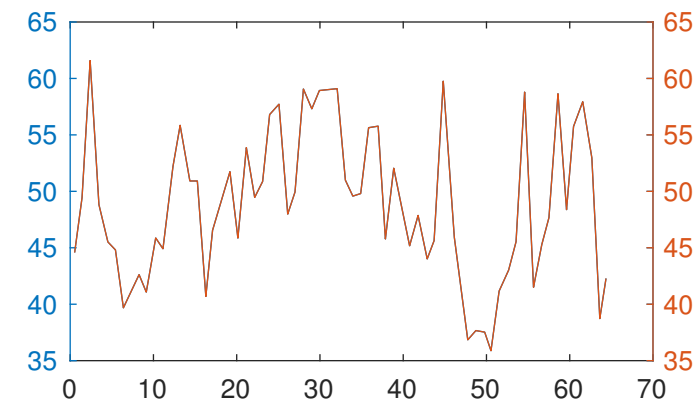

Supplement: S2 File — A zipped archive of all down-core plots and correlation tables used in this paper. (ZIP) [file pone.0199420.s006.zip › Downcore Plots and Correlation Tables/mejillones/figures/33C.depth.curves.pdf]

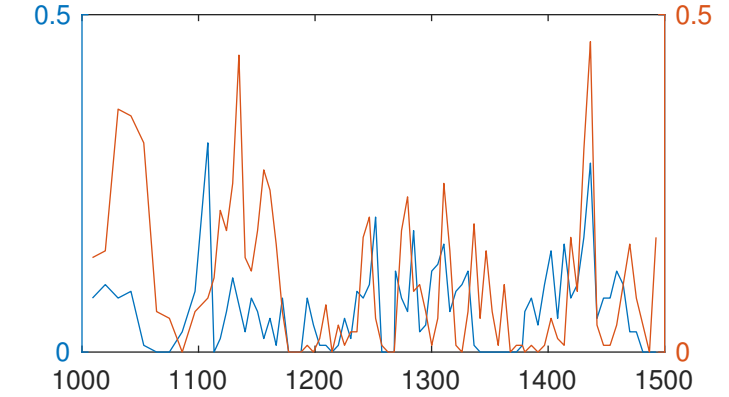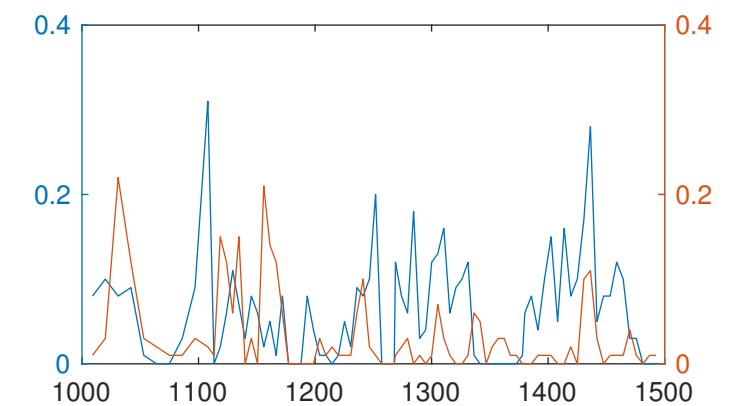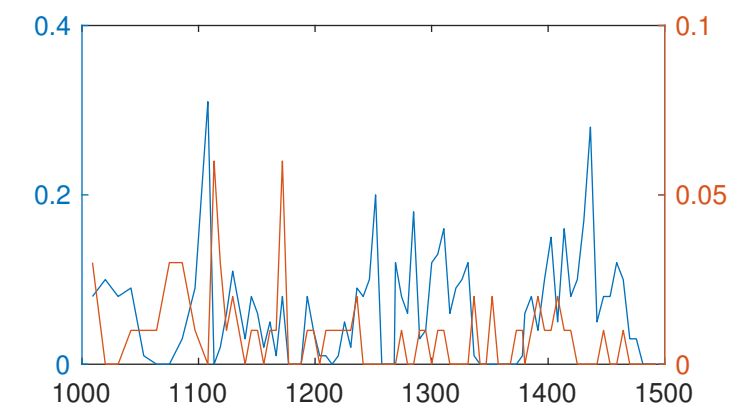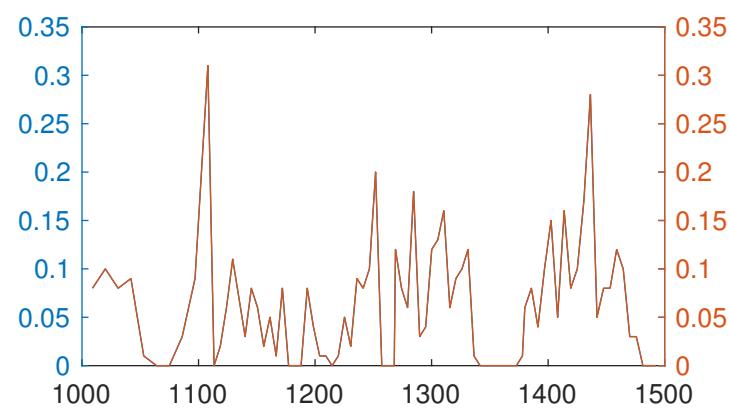

Supplement: S2 File — A zipped archive of all down-core plots and correlation tables used in this paper. (ZIP) [file pone.0199420.s006.zip › Downcore Plots and Correlation Tables/santabarbara/figures/SPR0901-02KC.year.curves.pdf]

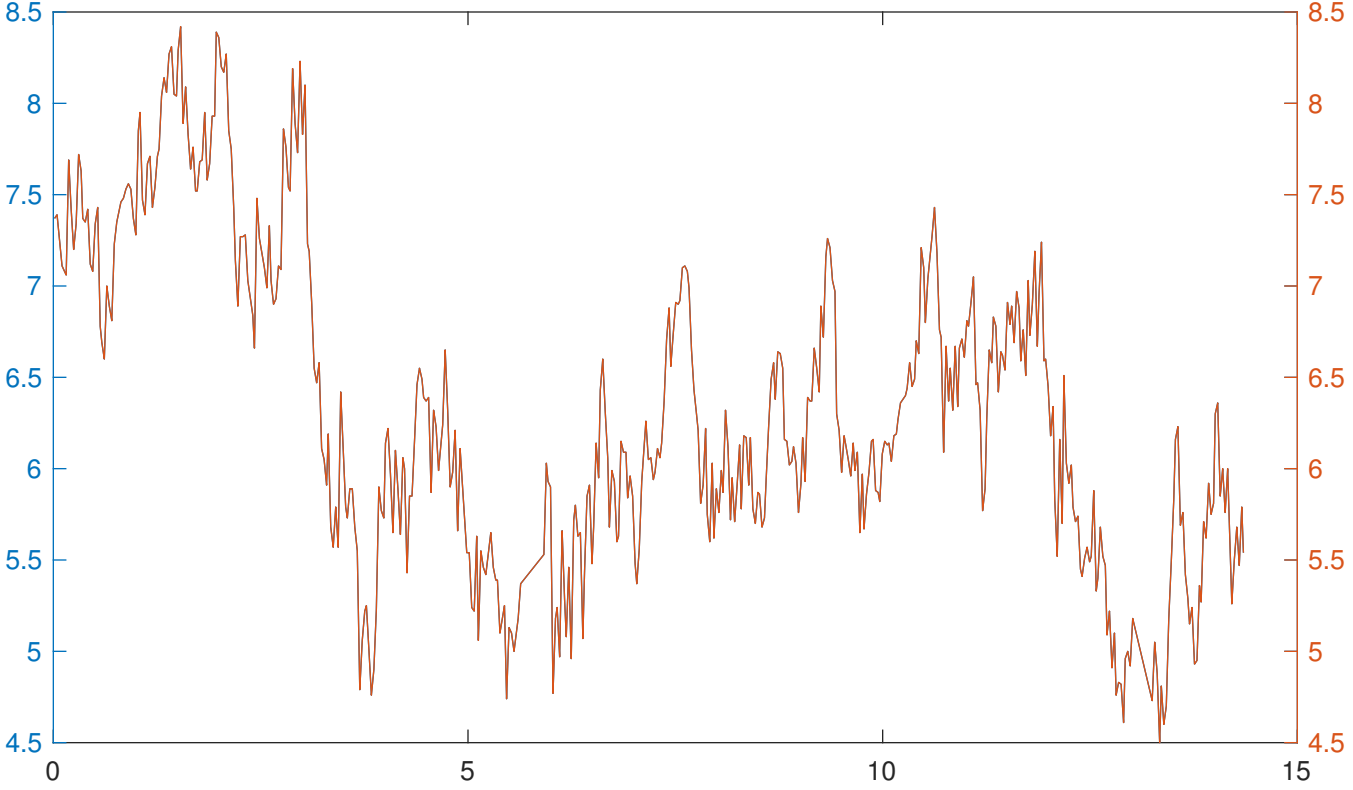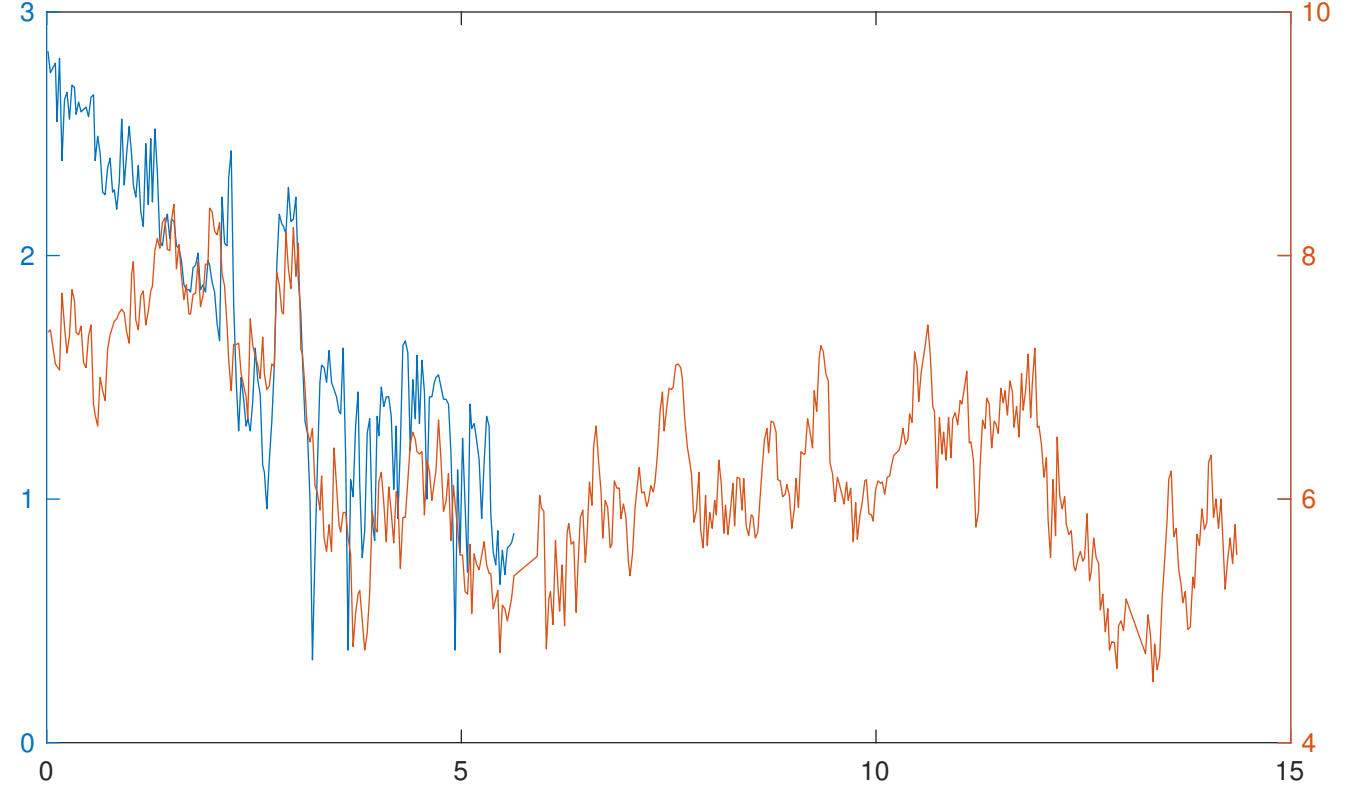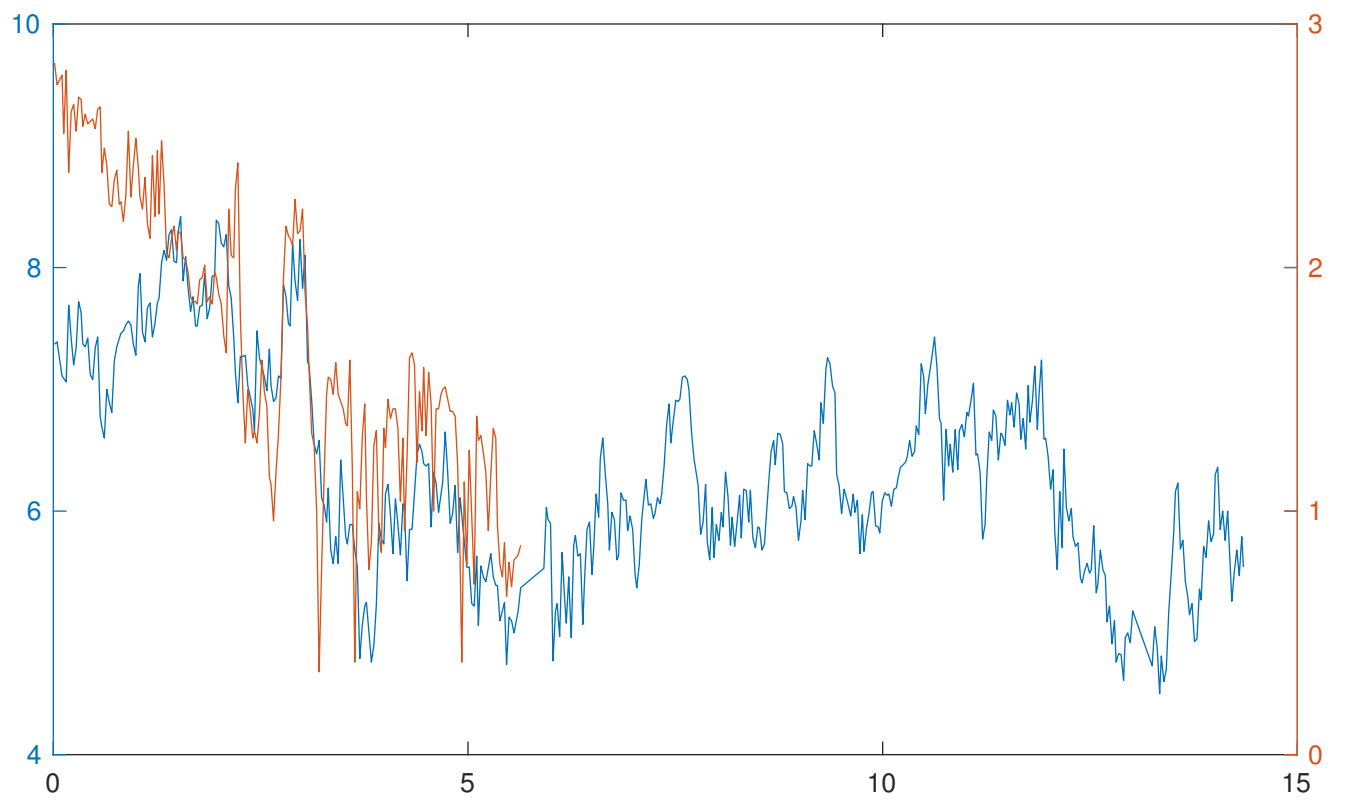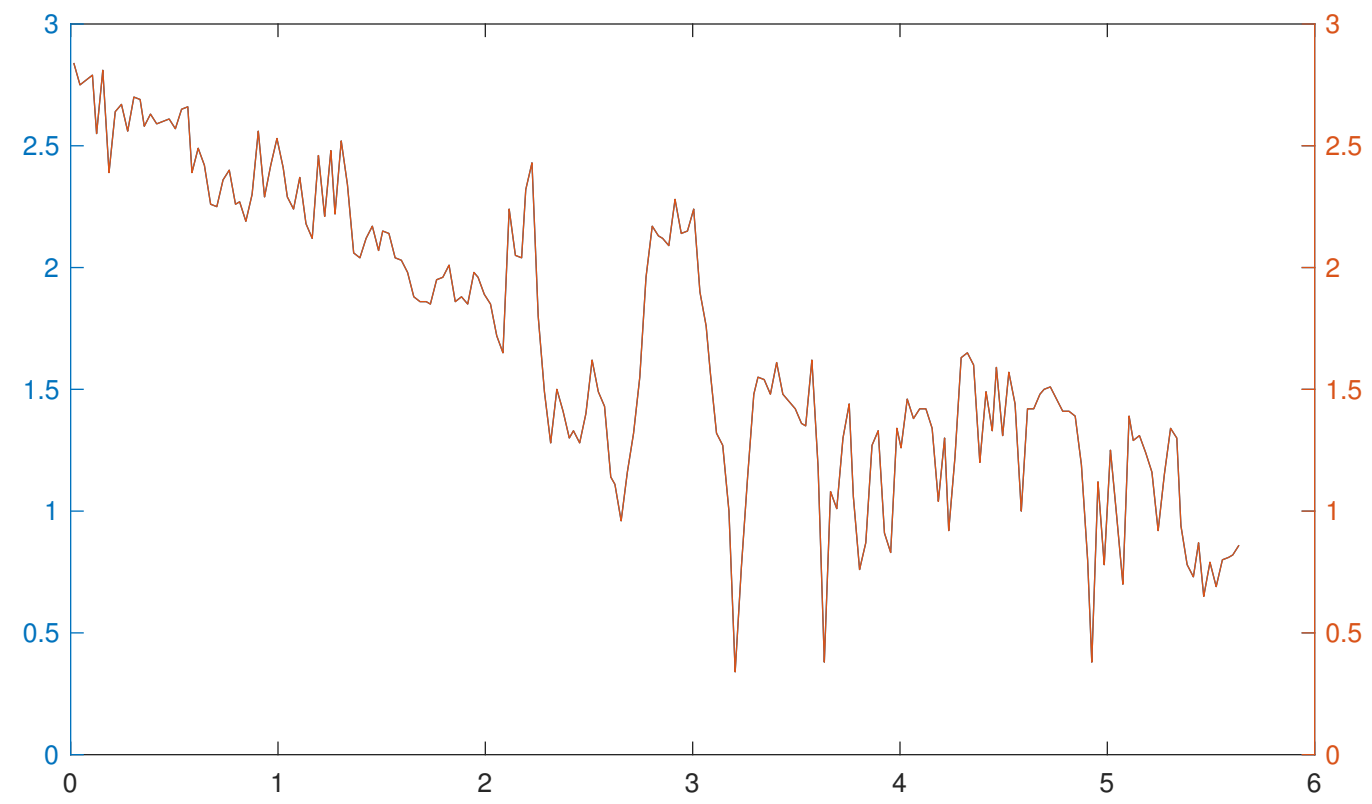

Supplement: S2 File — A zipped archive of all down-core plots and correlation tables used in this paper. (ZIP) [file pone.0199420.s006.zip › Downcore Plots and Correlation Tables/santabarbara/figures/ODP1017.depth.curves.pdf]

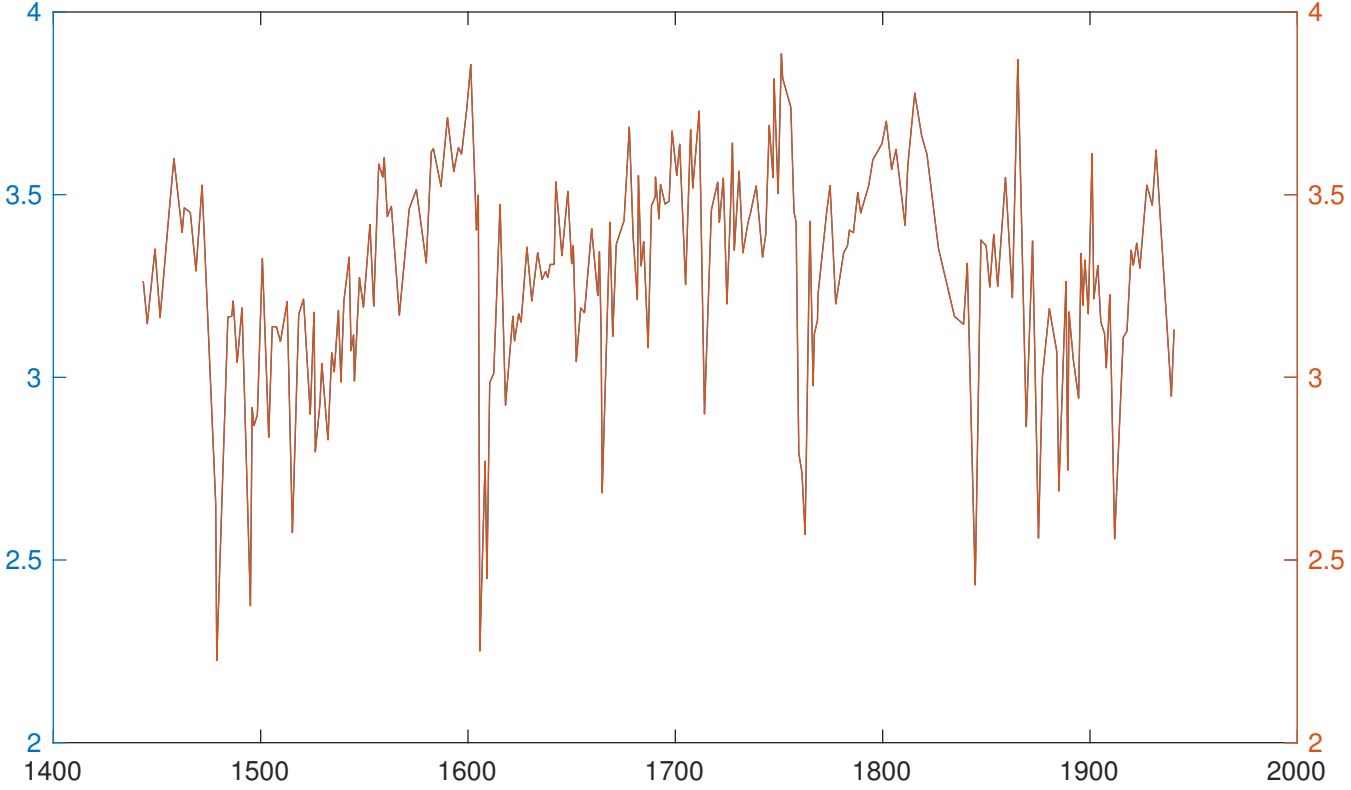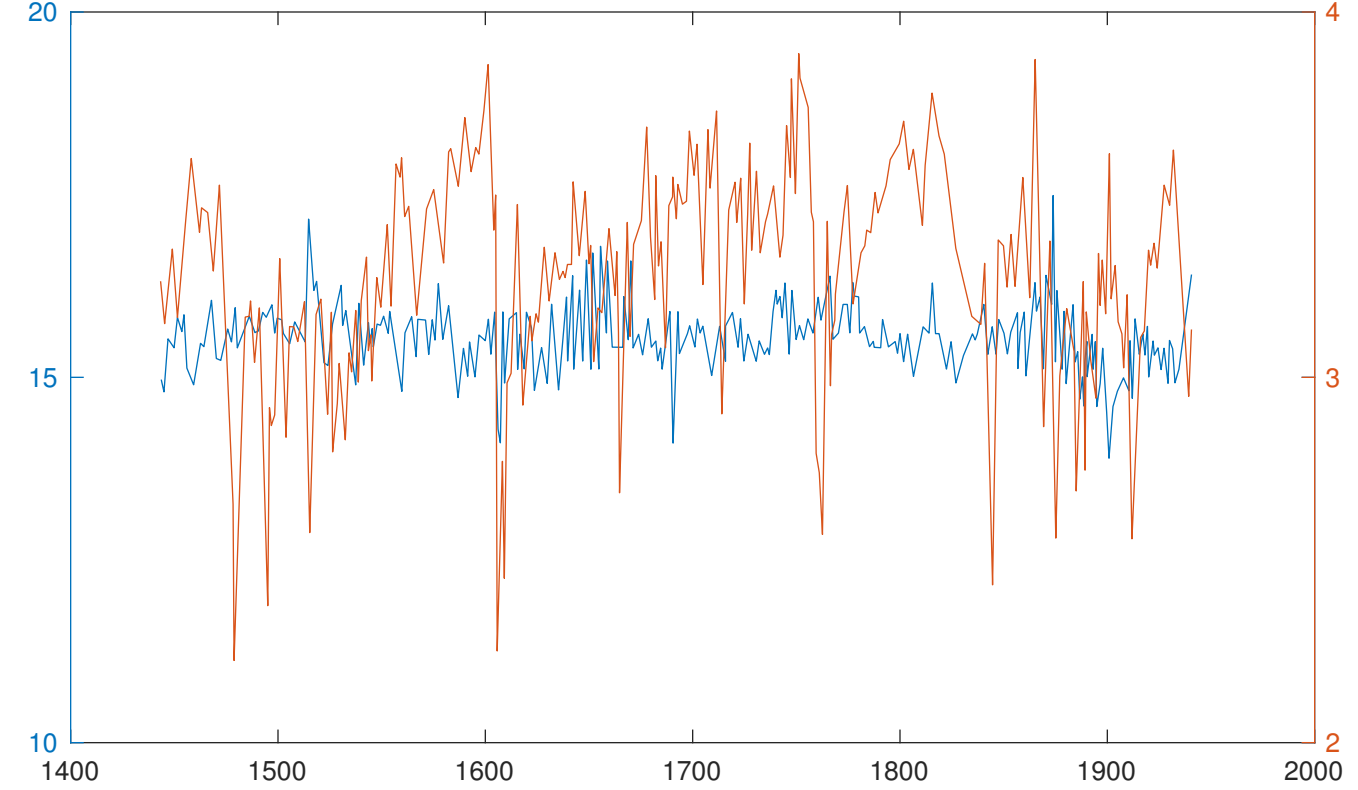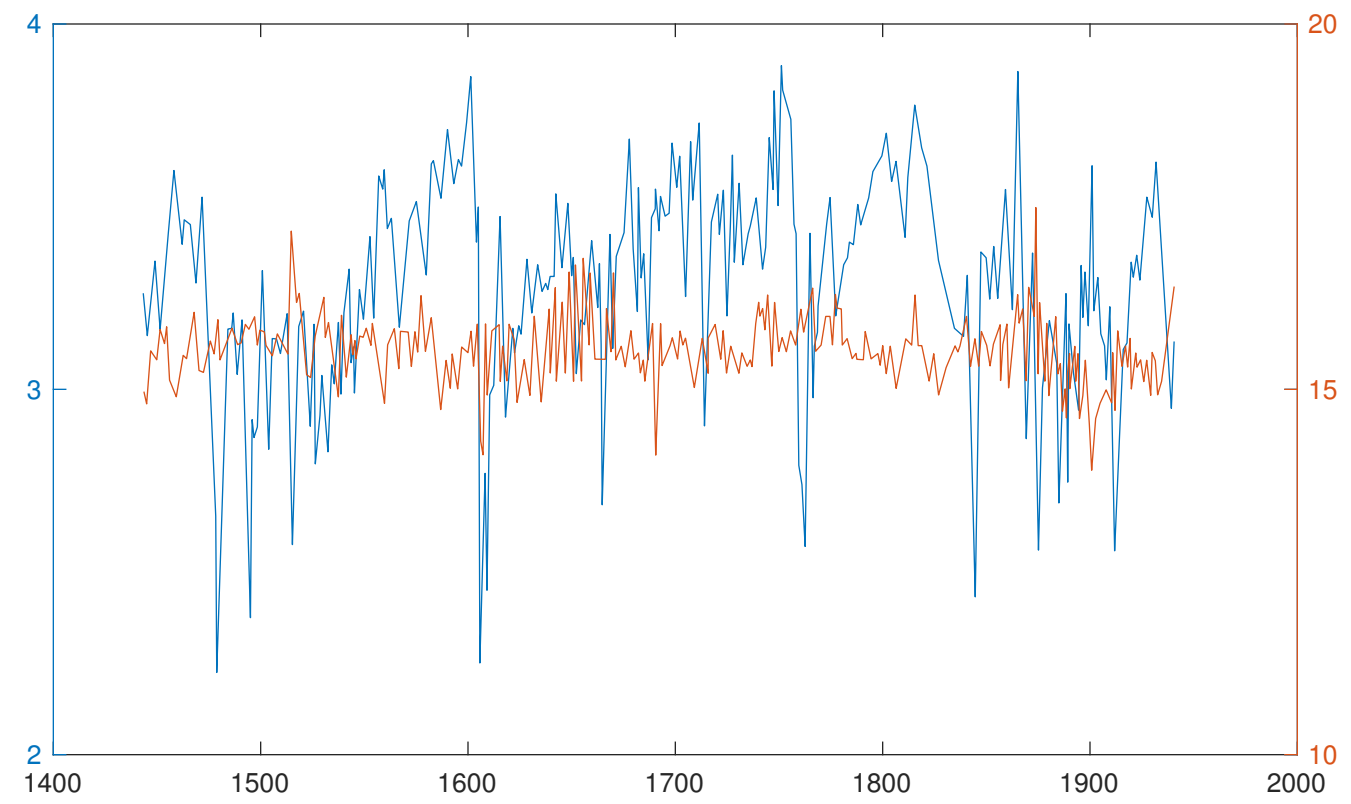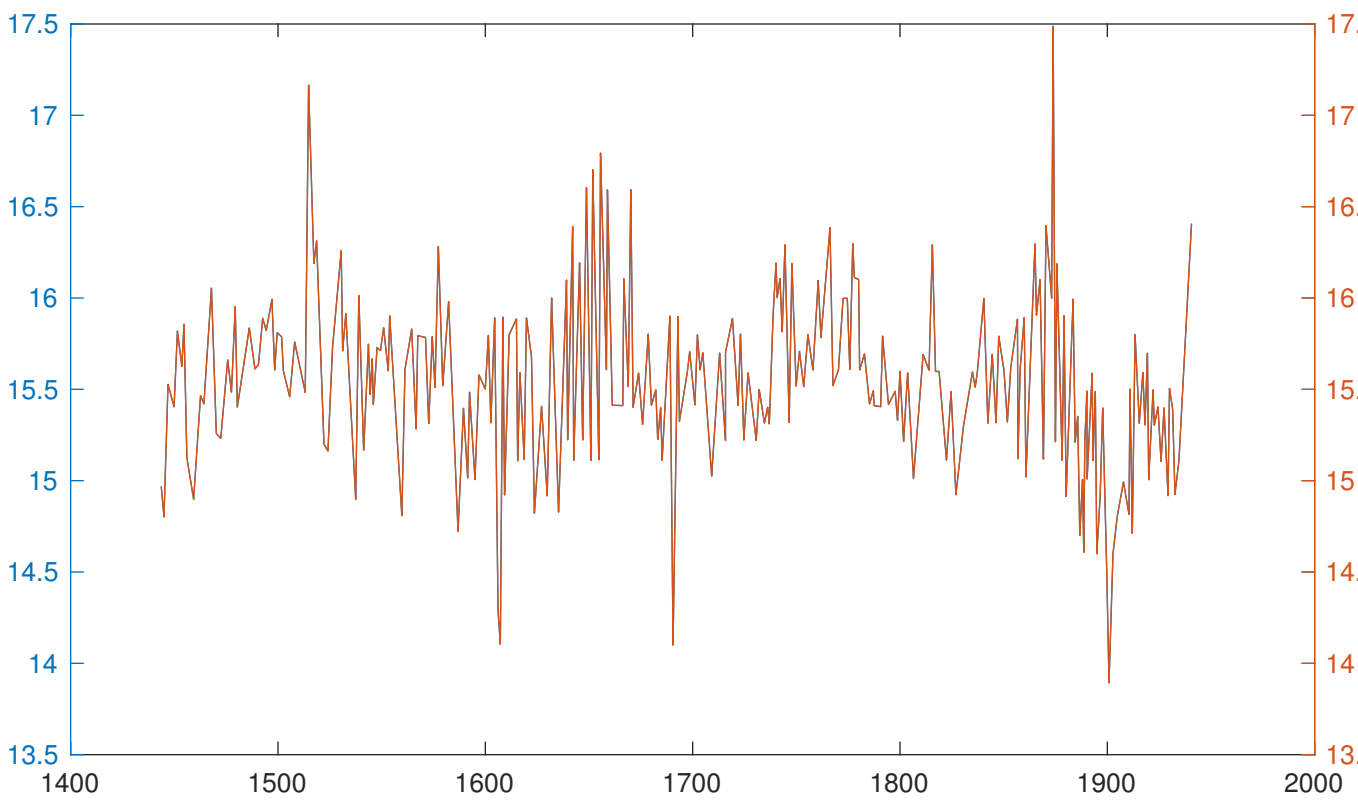

Supplement: S2 File — A zipped archive of all down-core plots and correlation tables used in this paper. (ZIP) [file pone.0199420.s006.zip › Downcore Plots and Correlation Tables/santabarbara/figures/SABA87-1.year.curves.pdf]

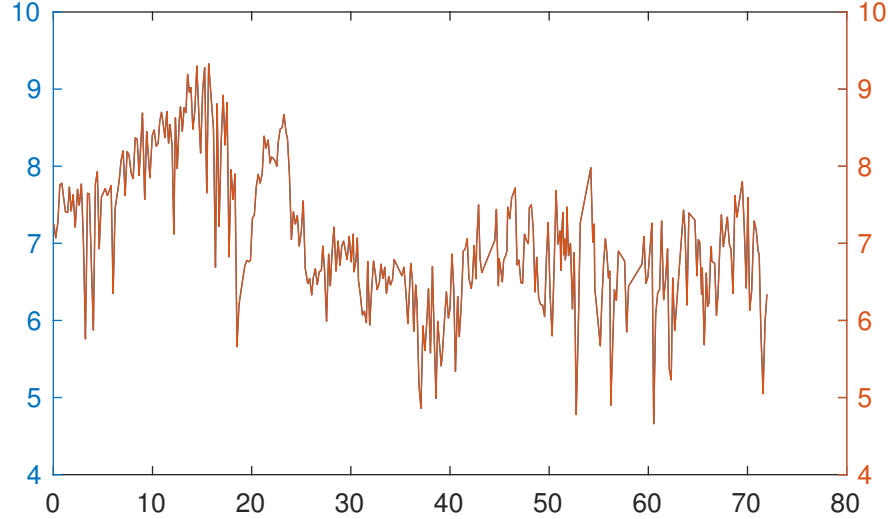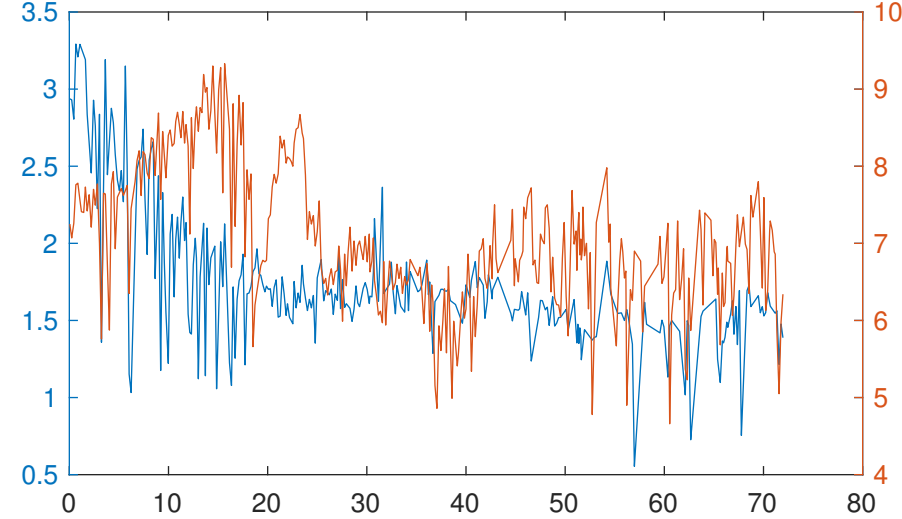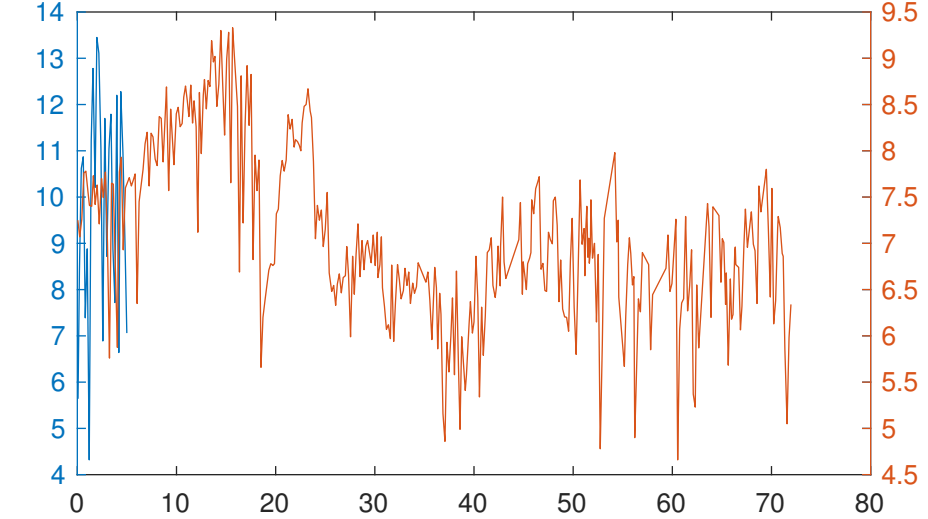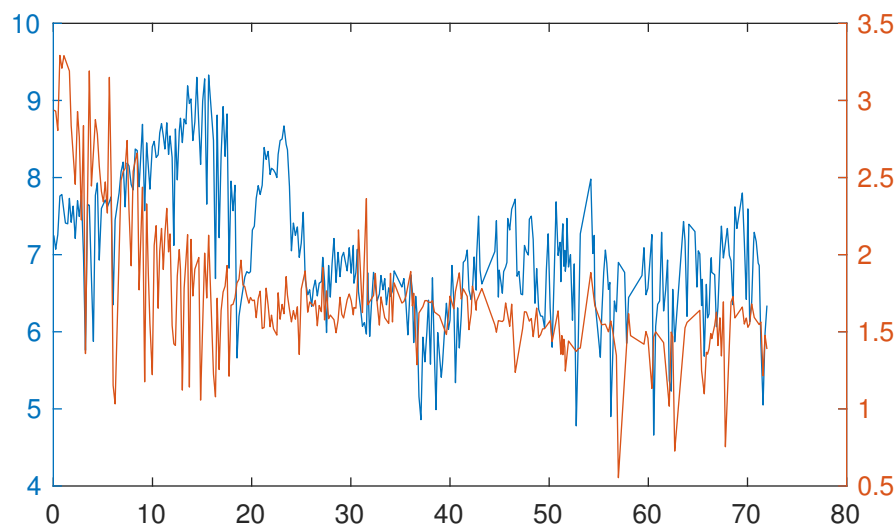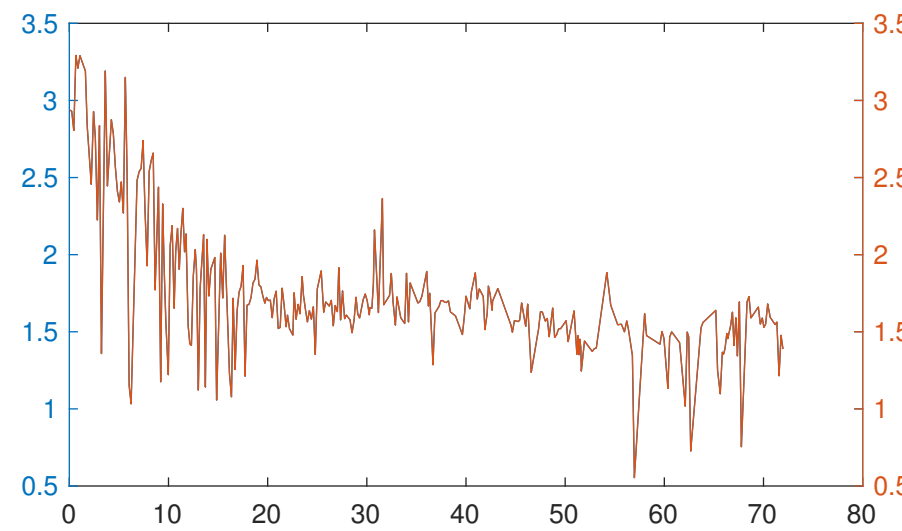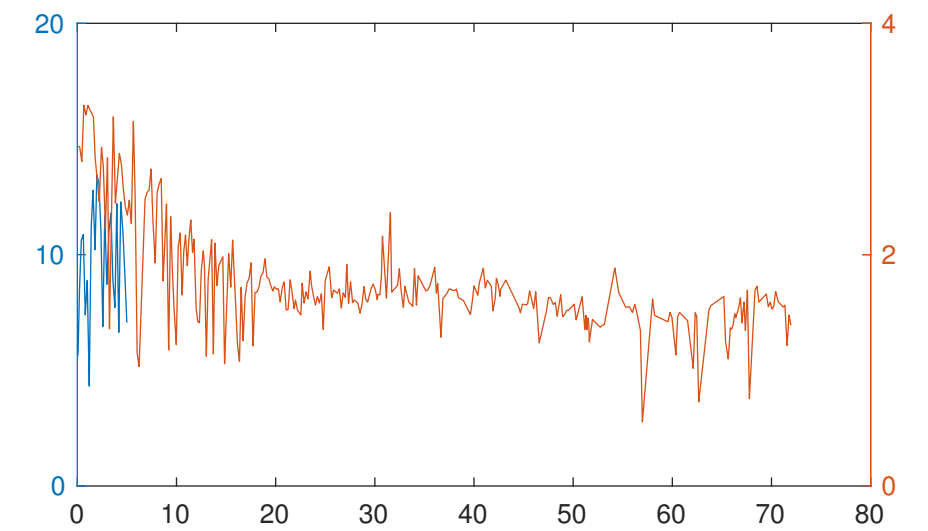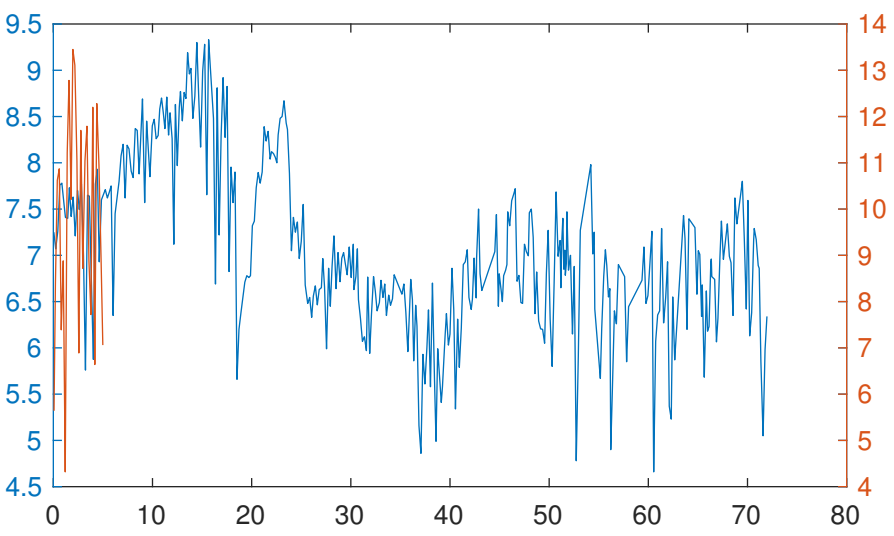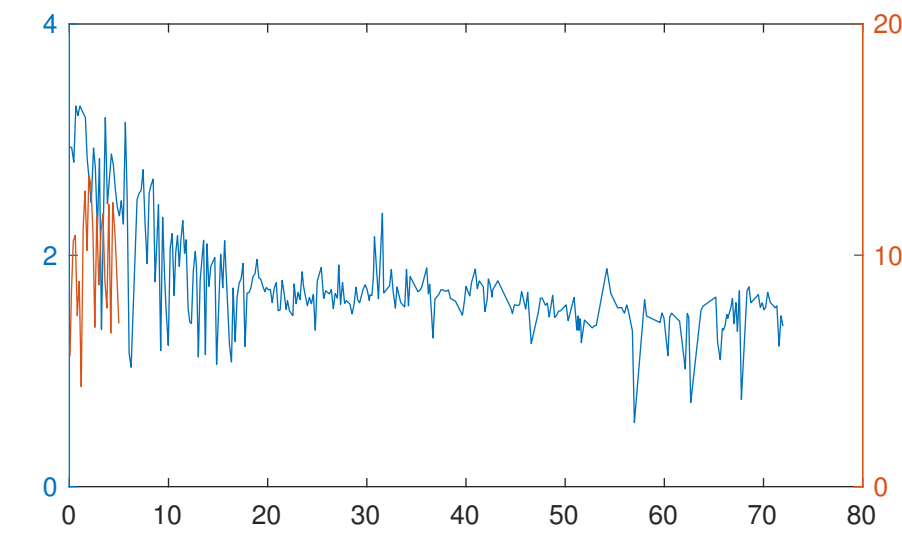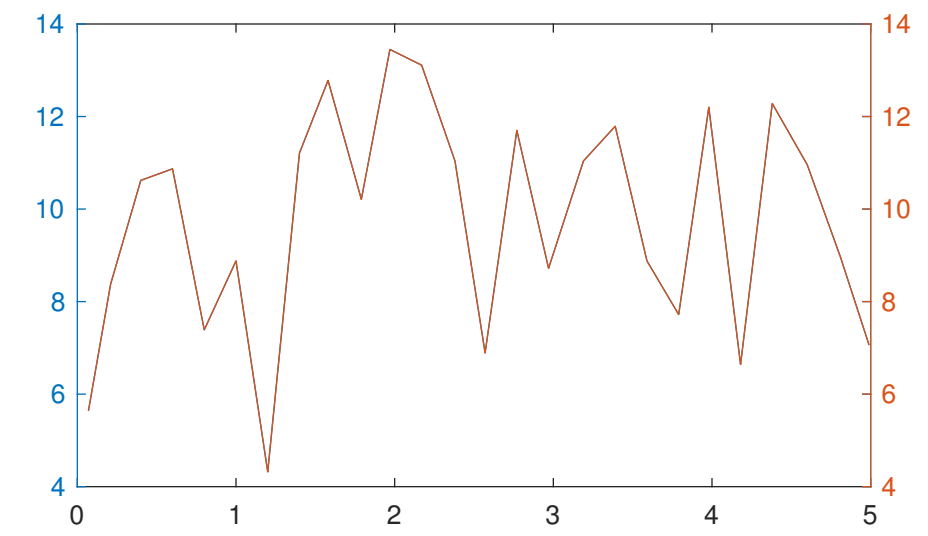

Supplement: S2 File — A zipped archive of all down-core plots and correlation tables used in this paper. (ZIP) [file pone.0199420.s006.zip › Downcore Plots and Correlation Tables/santabarbara/figures/ODP893.depth.curves.pdf]

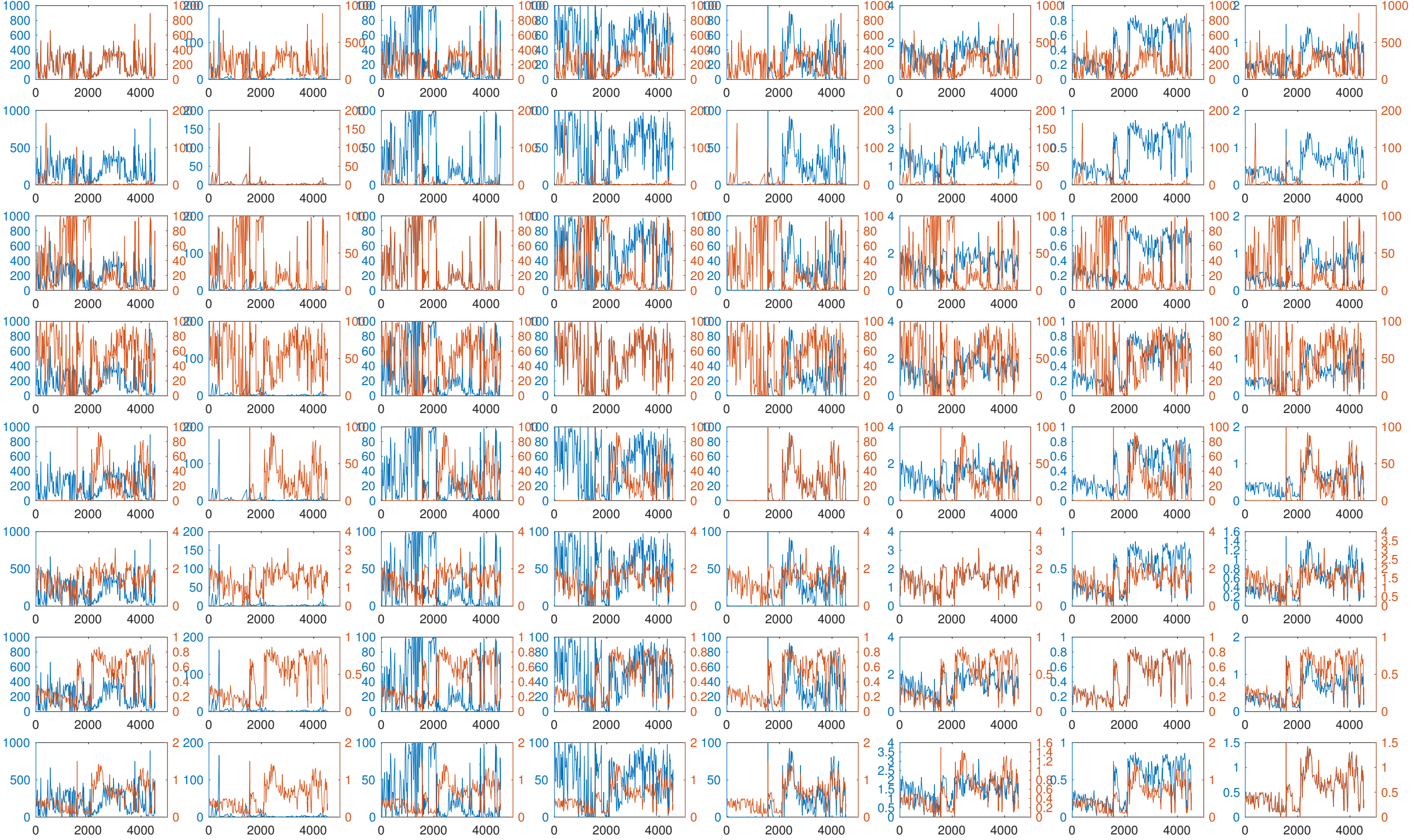

Supplement: S2 File — A zipped archive of all down-core plots and correlation tables used in this paper. (ZIP) [file pone.0199420.s006.zip › Downcore Plots and Correlation Tables/santabarbara/figures/MD2503.depth.curves.pdf]

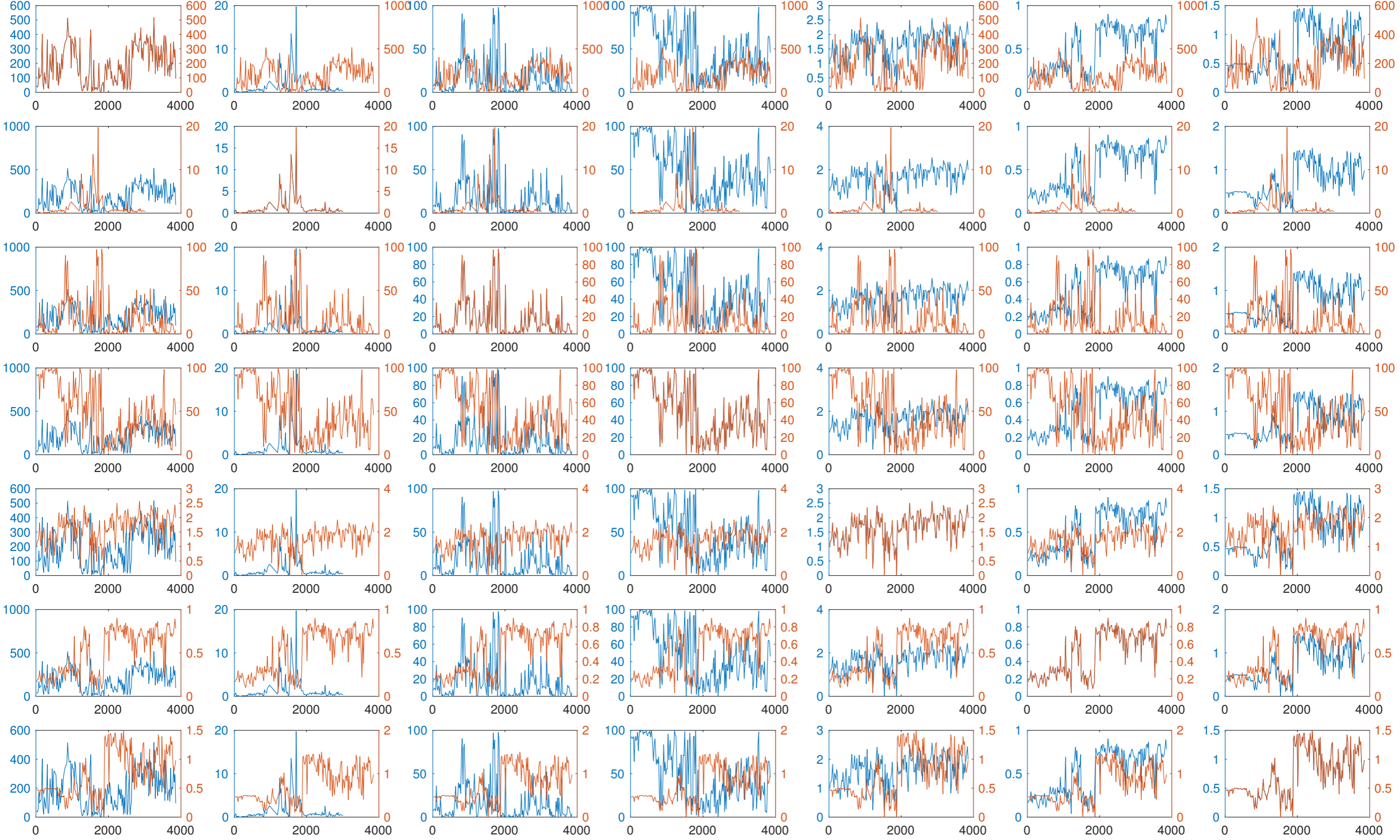

Supplement: S2 File — A zipped archive of all down-core plots and correlation tables used in this paper. (ZIP) [file pone.0199420.s006.zip › Downcore Plots and Correlation Tables/santabarbara/figures/MD2504.depth.curves.pdf]

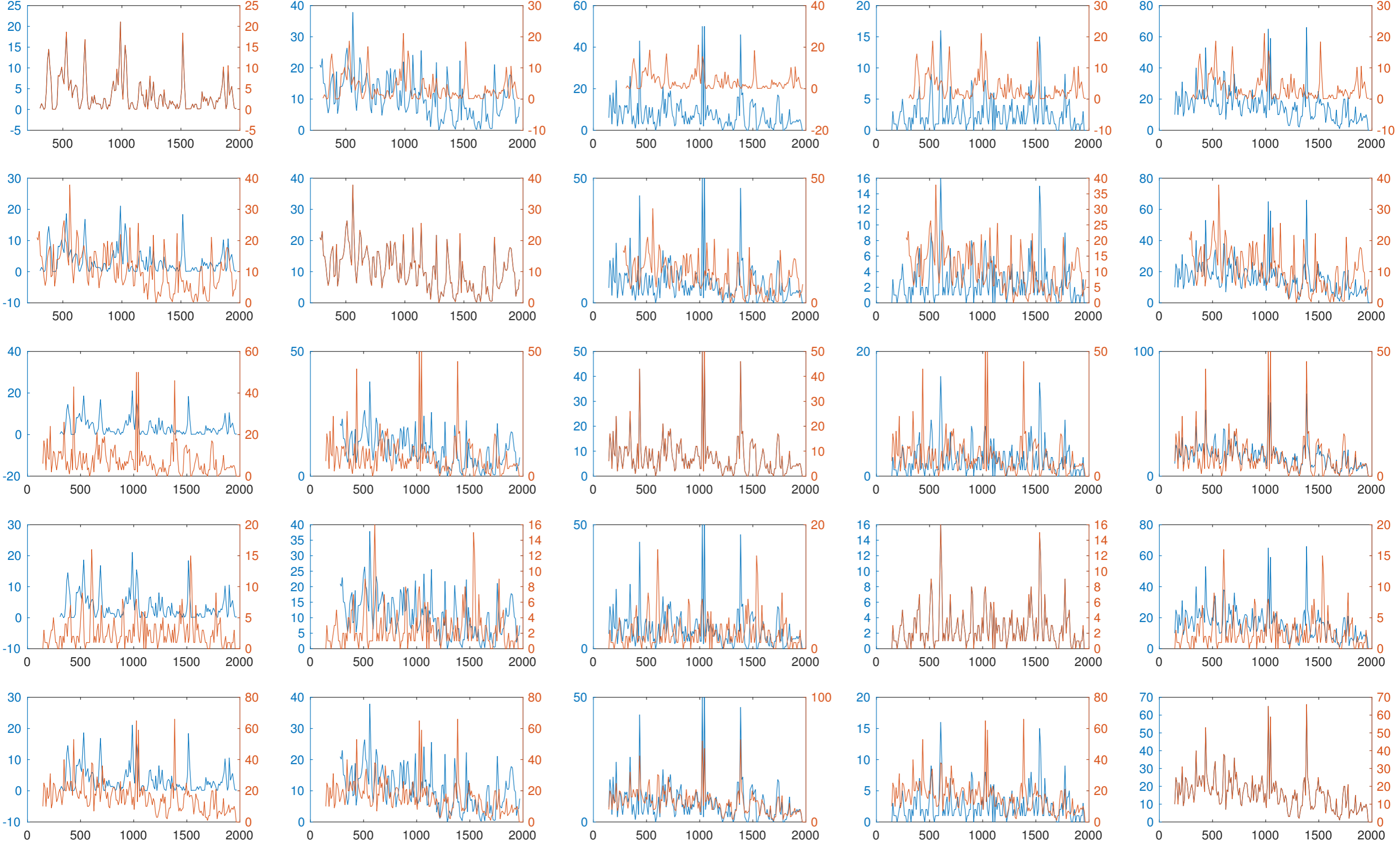

Supplement: S2 File — A zipped archive of all down-core plots and correlation tables used in this paper. (ZIP) [file pone.0199420.s006.zip › Downcore Plots and Correlation Tables/santabarbara/figures/214.year.curves.pdf]

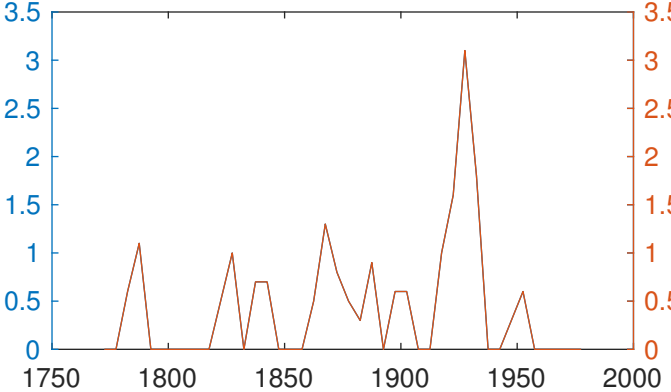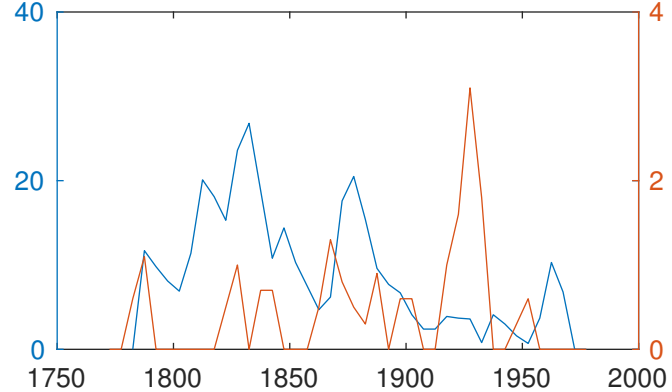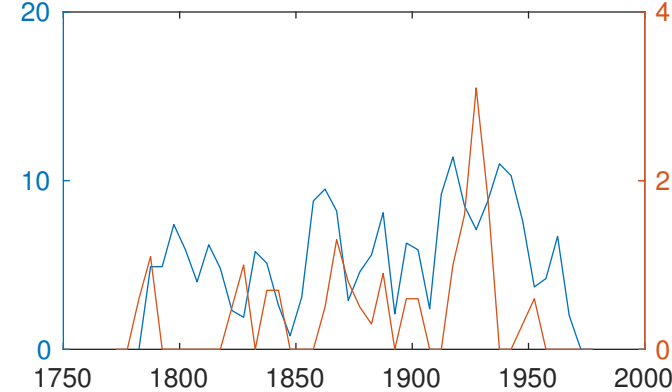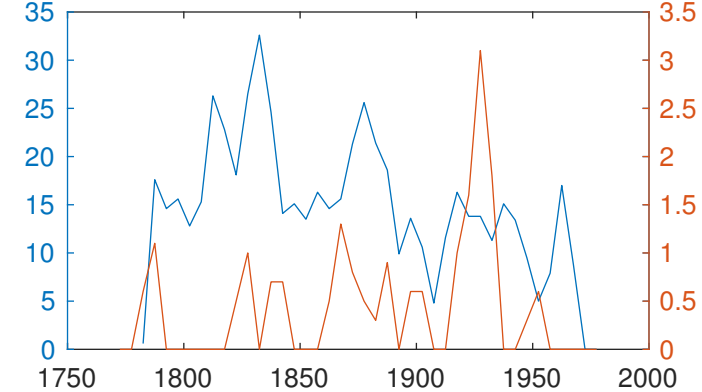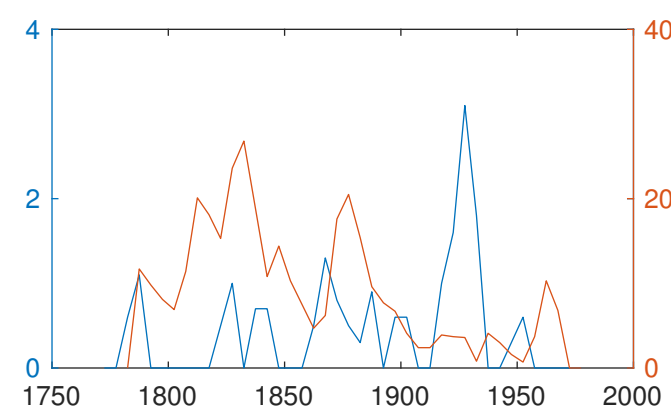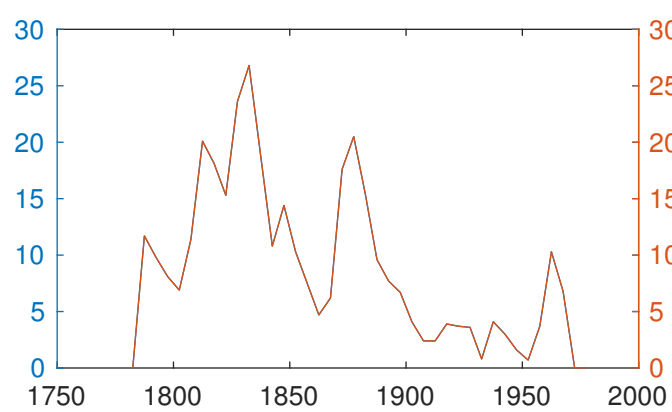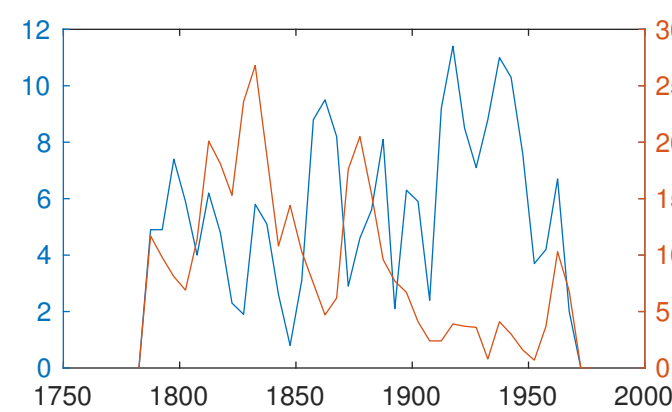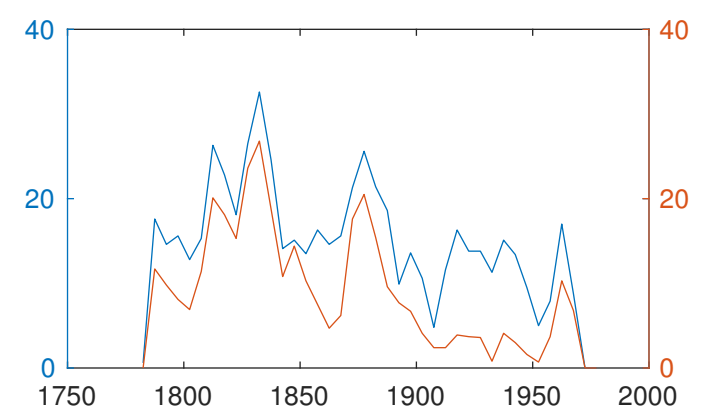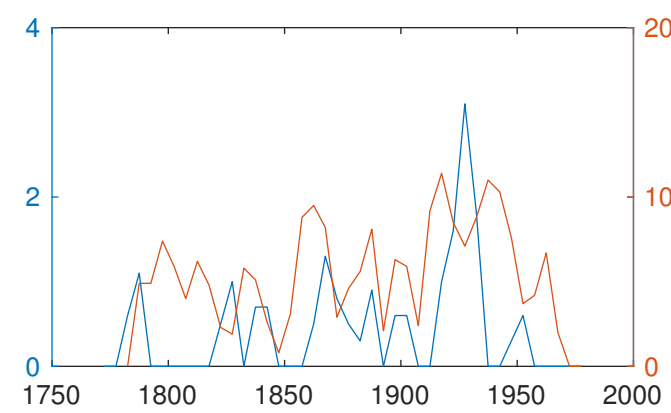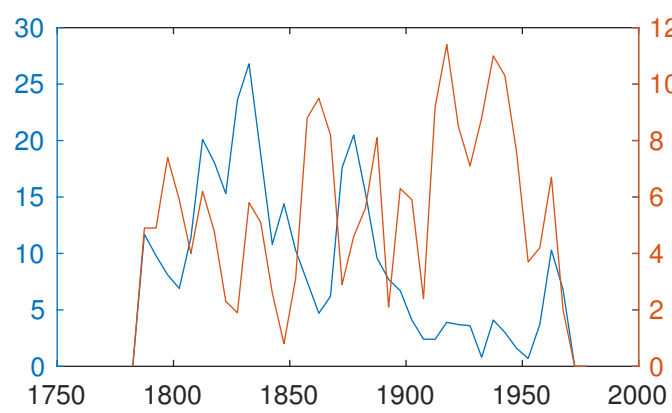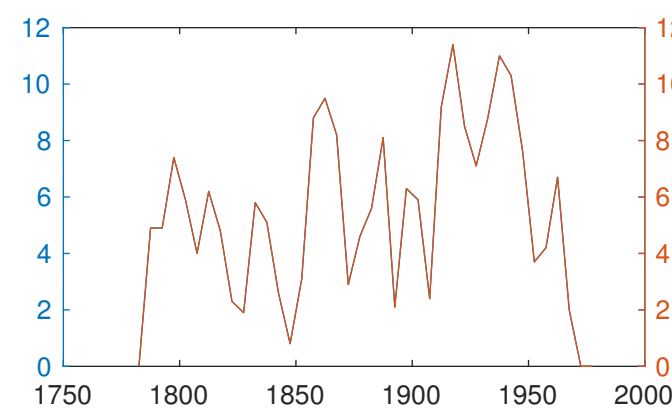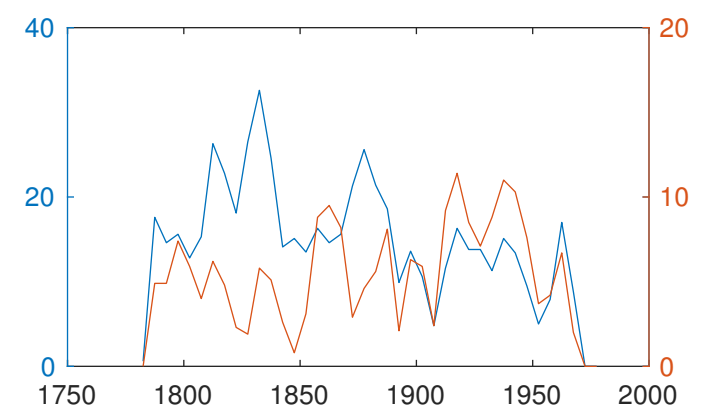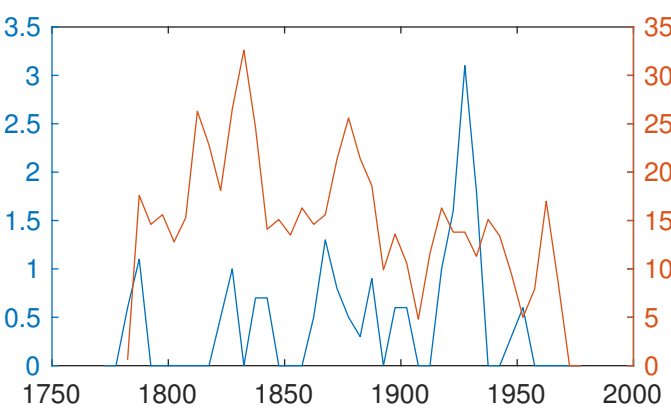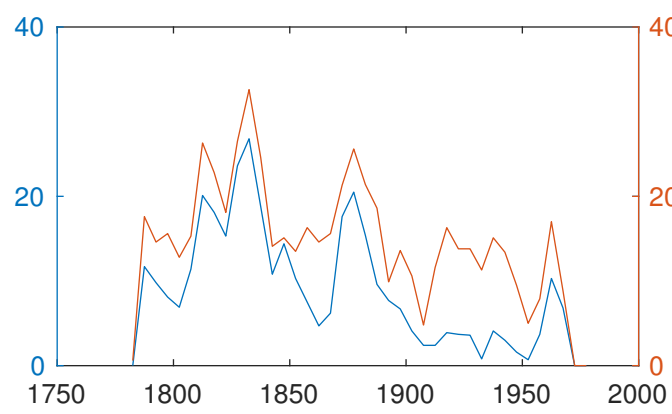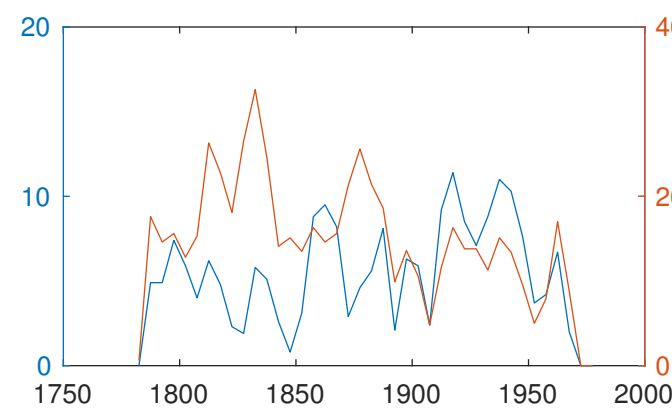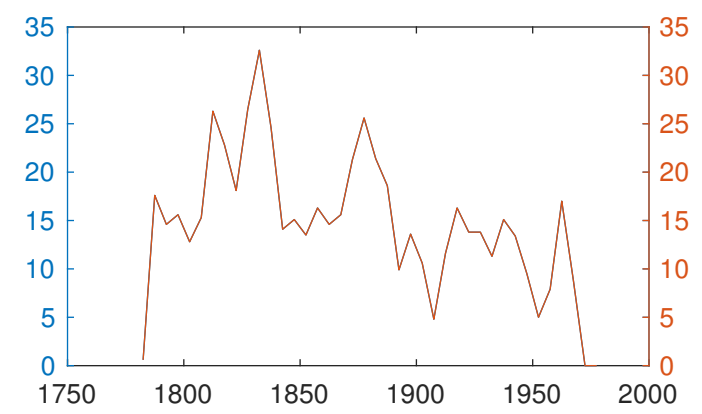

Supplement: S2 File — A zipped archive of all down-core plots and correlation tables used in this paper. (ZIP) [file pone.0199420.s006.zip › Downcore Plots and Correlation Tables/soledad/figures/244.year.curves.pdf]

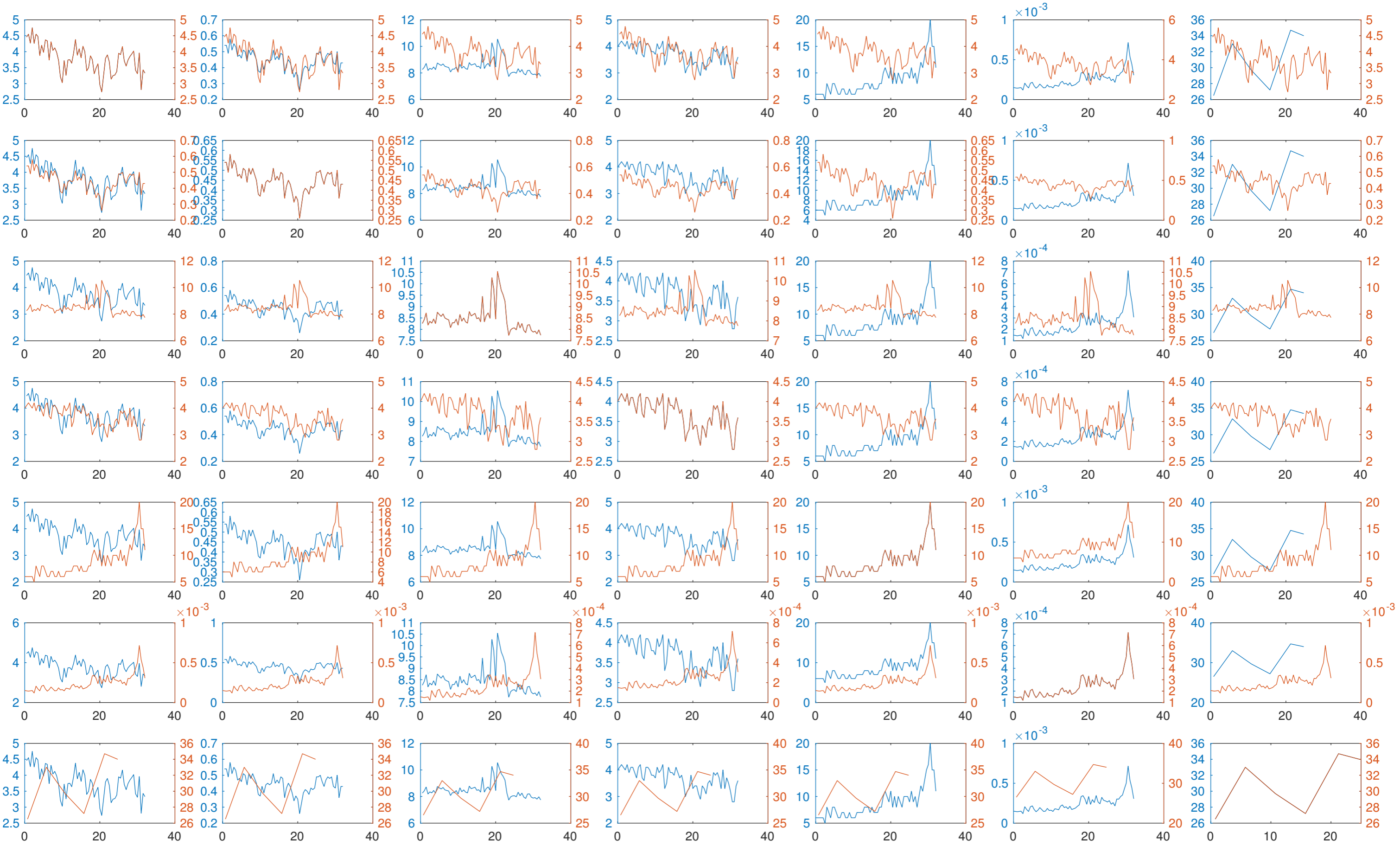

Supplement: S2 File — A zipped archive of all down-core plots and correlation tables used in this paper. (ZIP) [file pone.0199420.s006.zip › Downcore Plots and Correlation Tables/guaymas/figures/BC50.depth.curves.pdf]

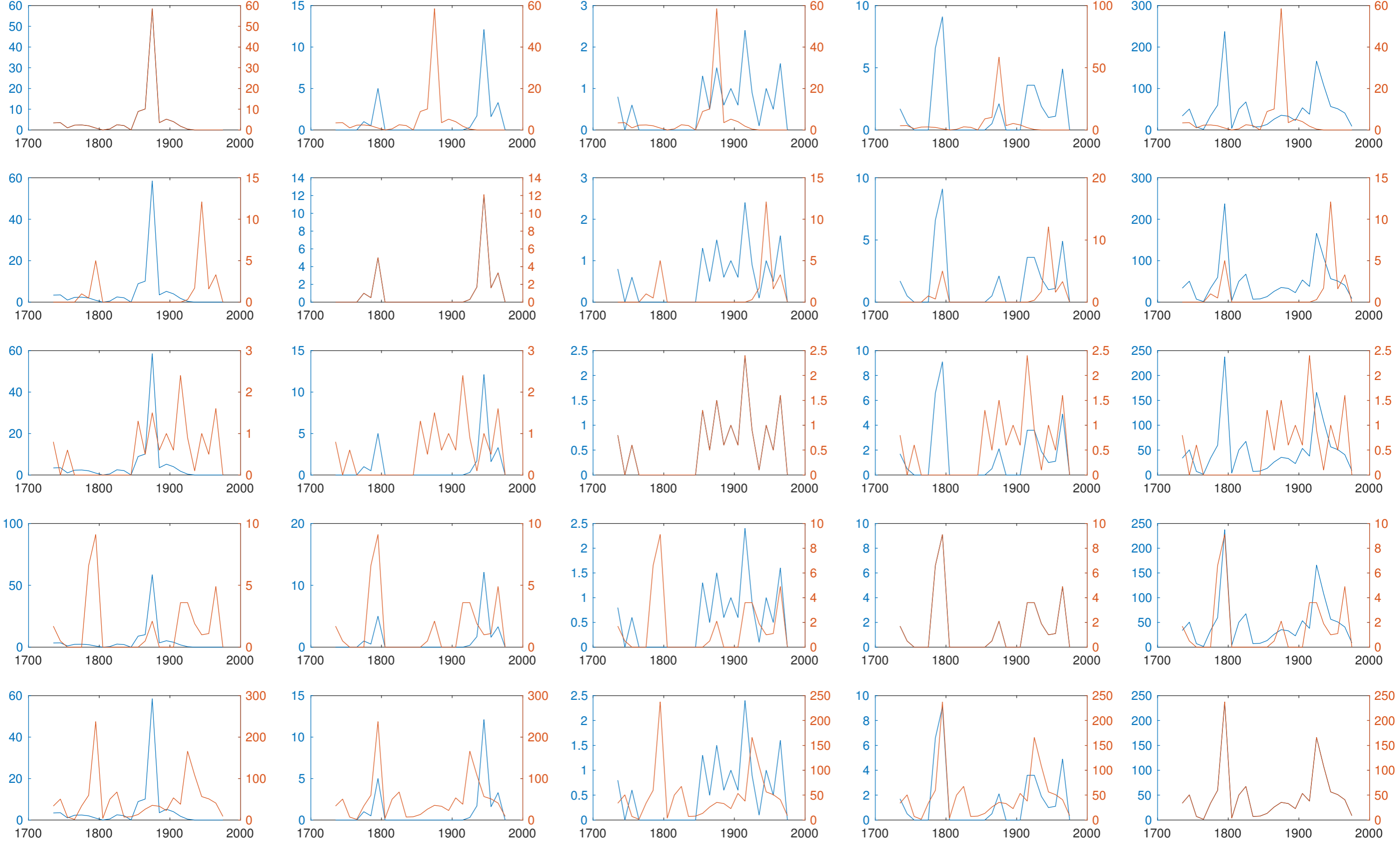

Supplement: S2 File — A zipped archive of all down-core plots and correlation tables used in this paper. (ZIP) [file pone.0199420.s006.zip › Downcore Plots and Correlation Tables/guaymas/figures/7807-1305.year.curves.pdf]

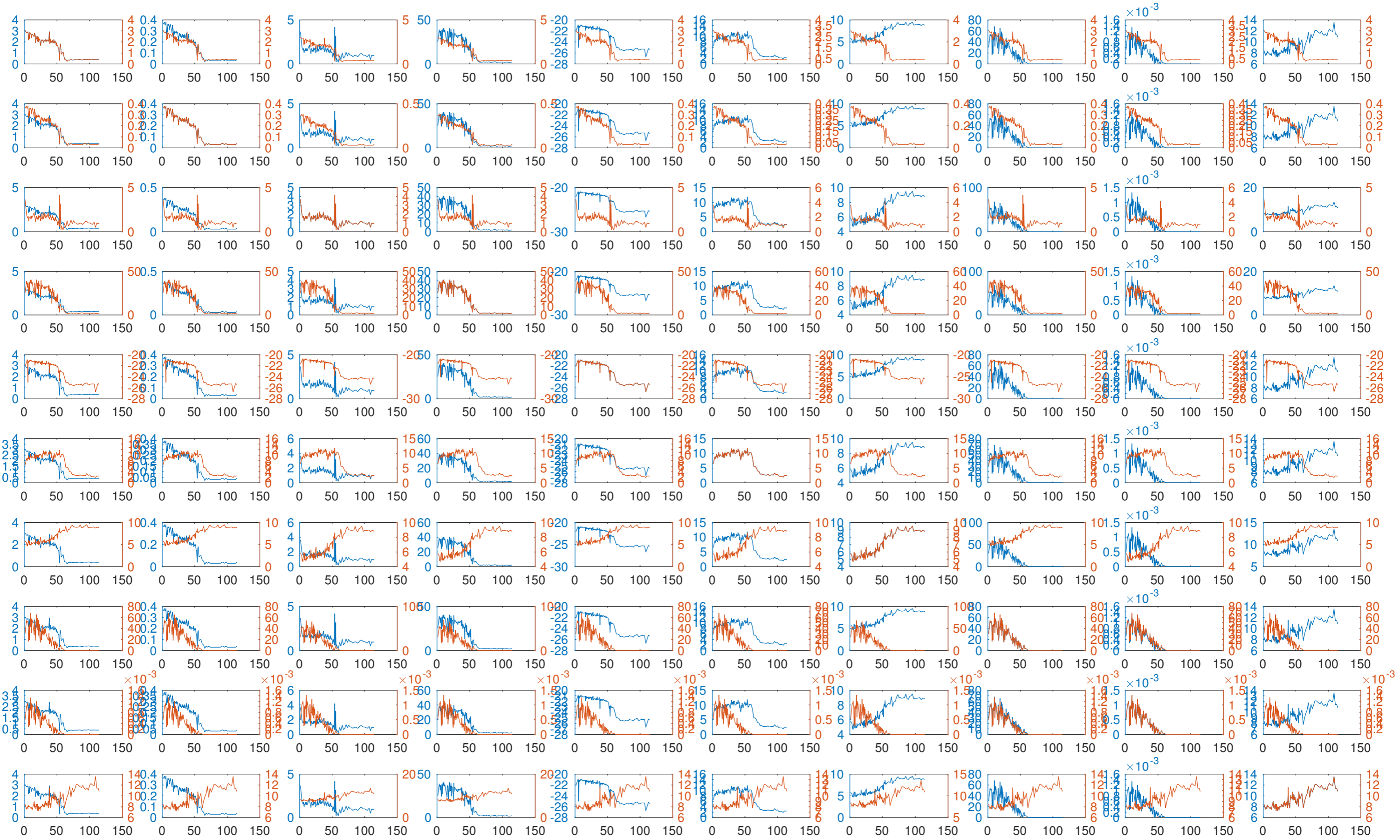

Supplement: S2 File — A zipped archive of all down-core plots and correlation tables used in this paper. (ZIP) [file pone.0199420.s006.zip › Downcore Plots and Correlation Tables/saanich/figures/ODP1033.depth.curves.pdf]

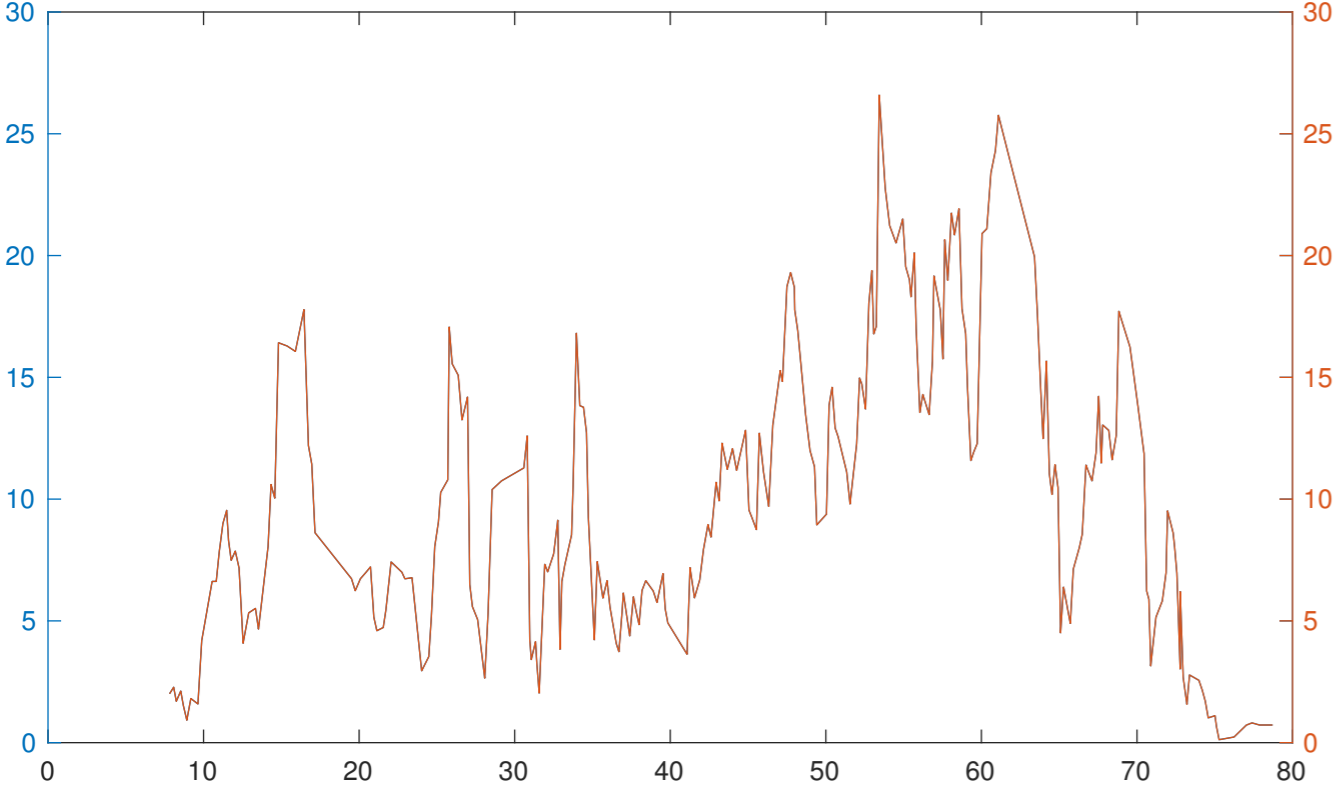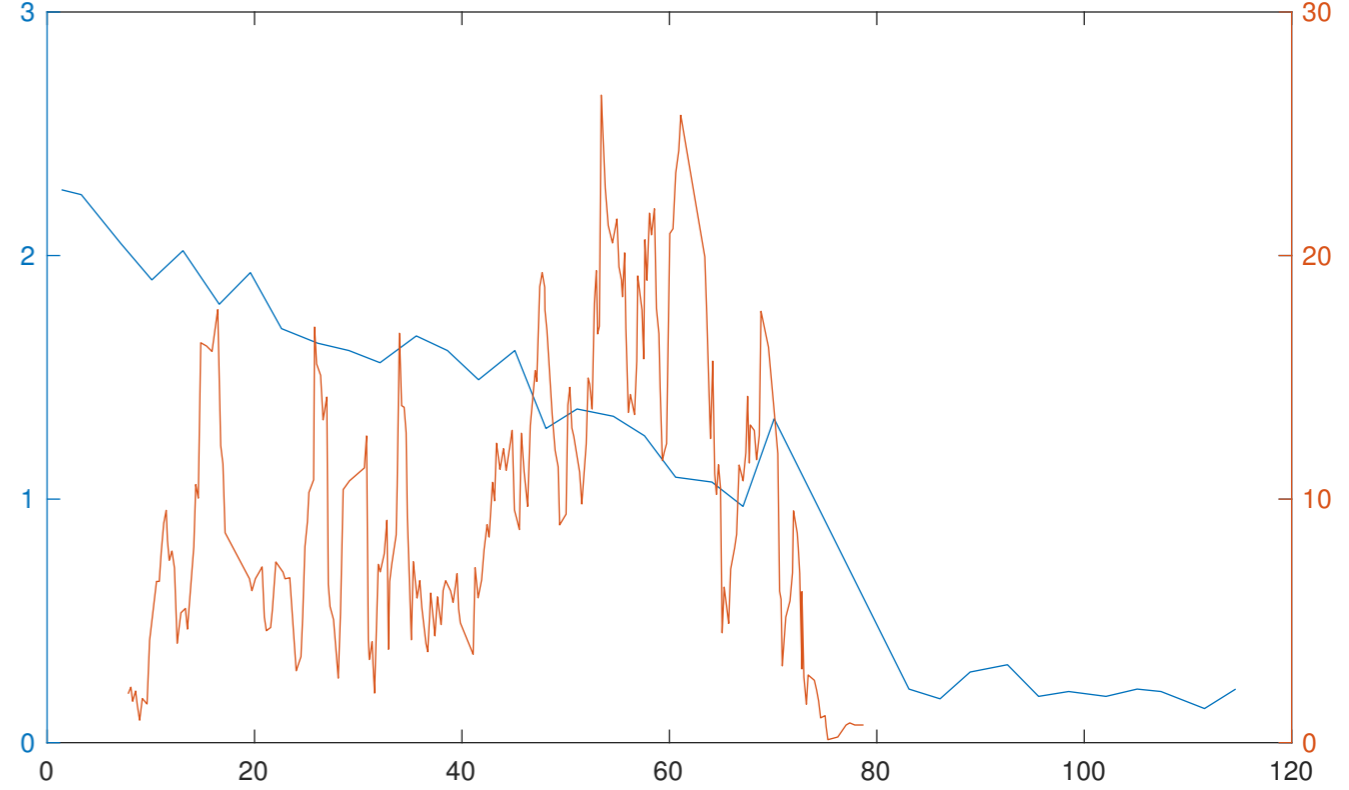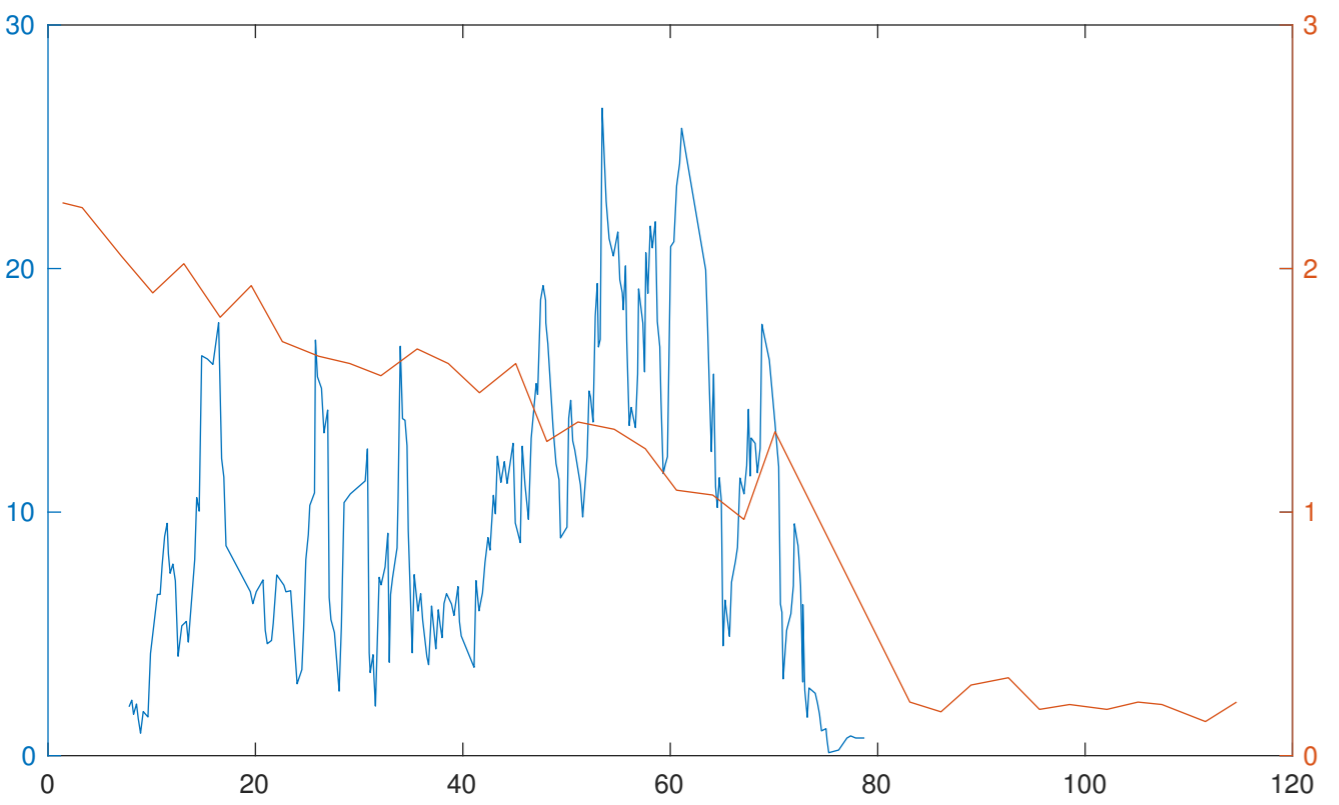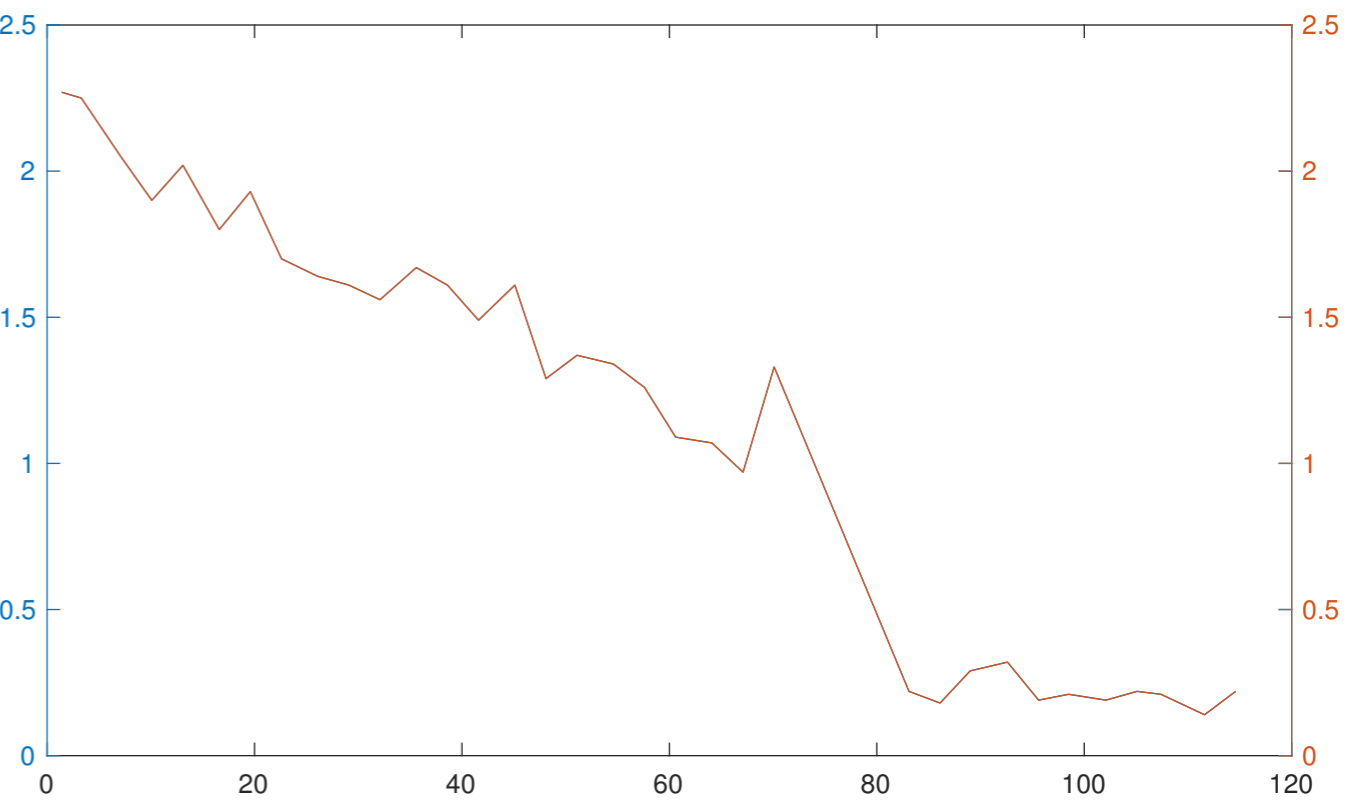

Supplement: S2 File — A zipped archive of all down-core plots and correlation tables used in this paper. (ZIP) [file pone.0199420.s006.zip › Downcore Plots and Correlation Tables/saanich/figures/ODP1034.depth.curves.pdf]

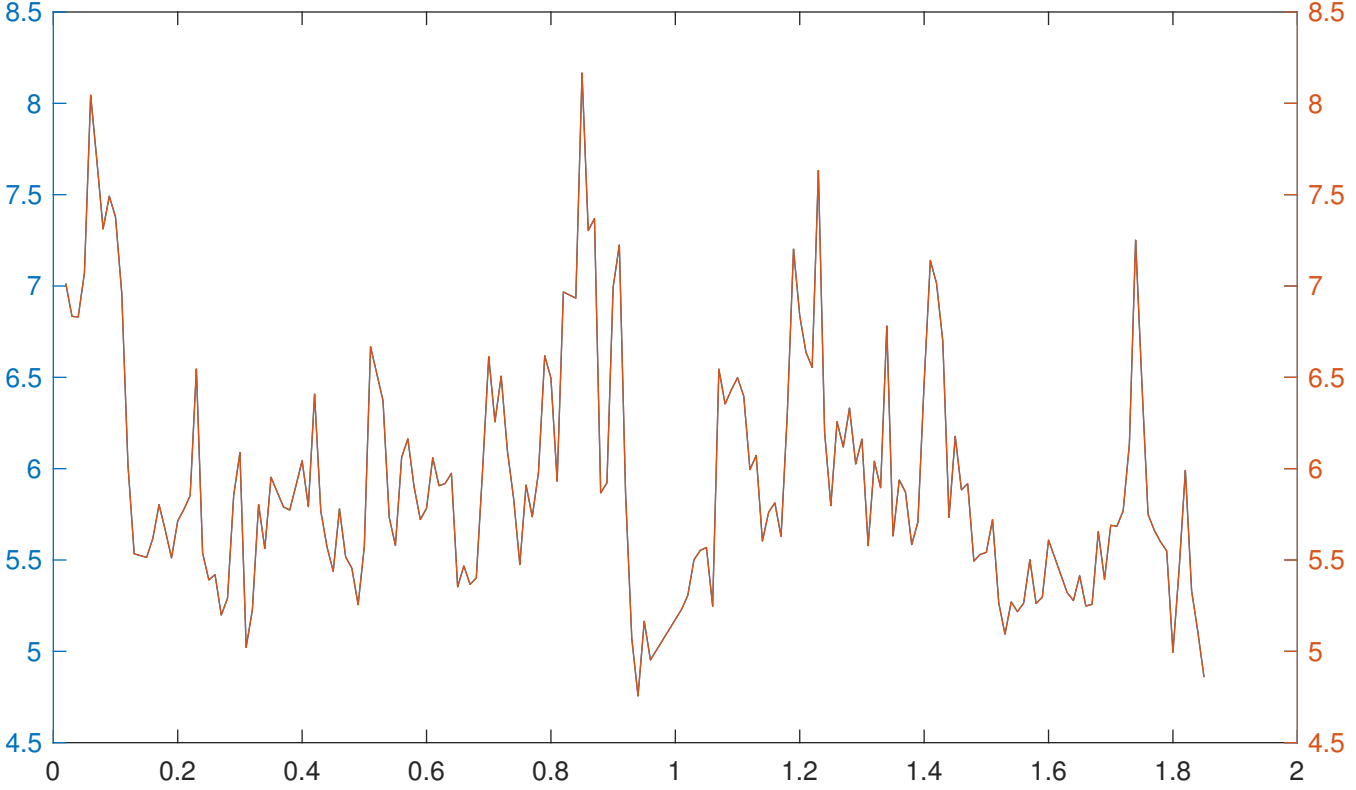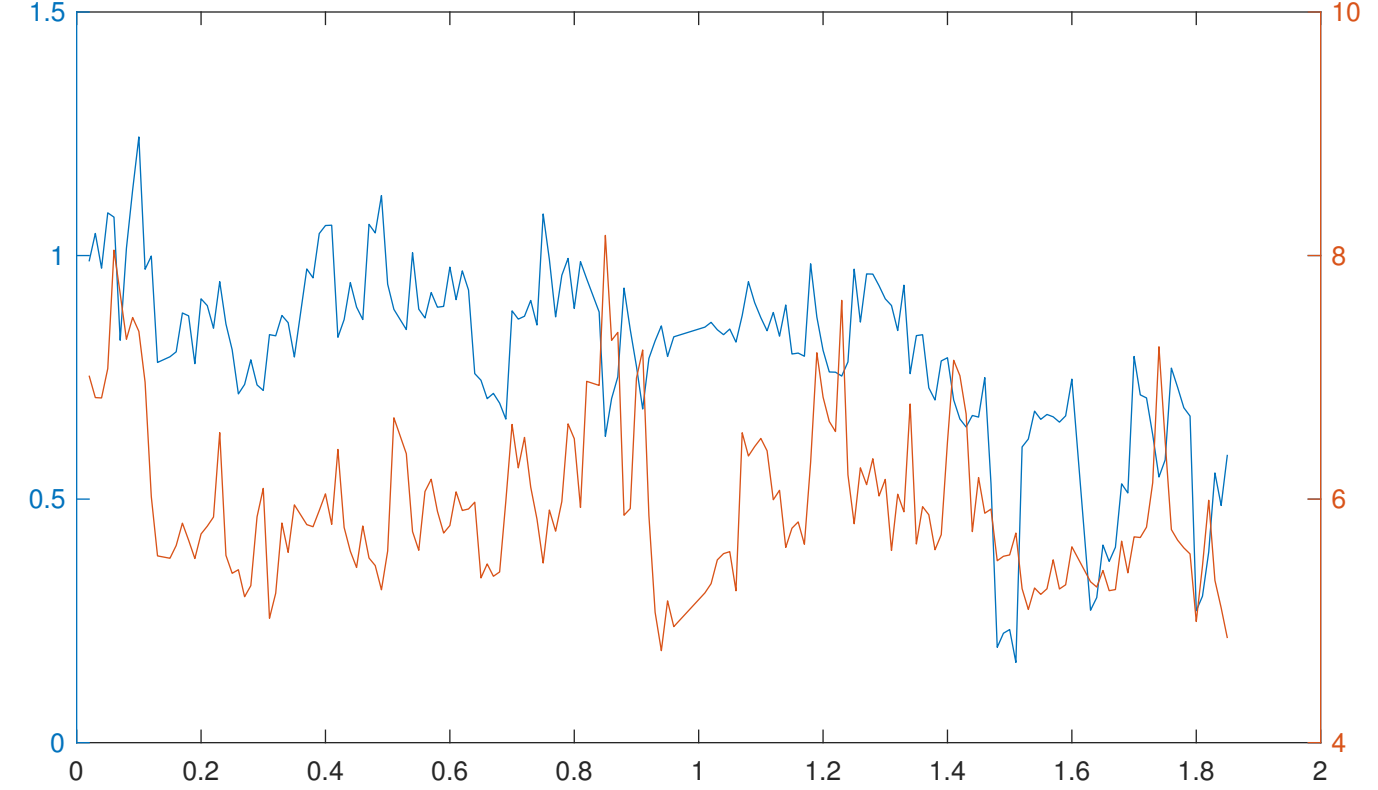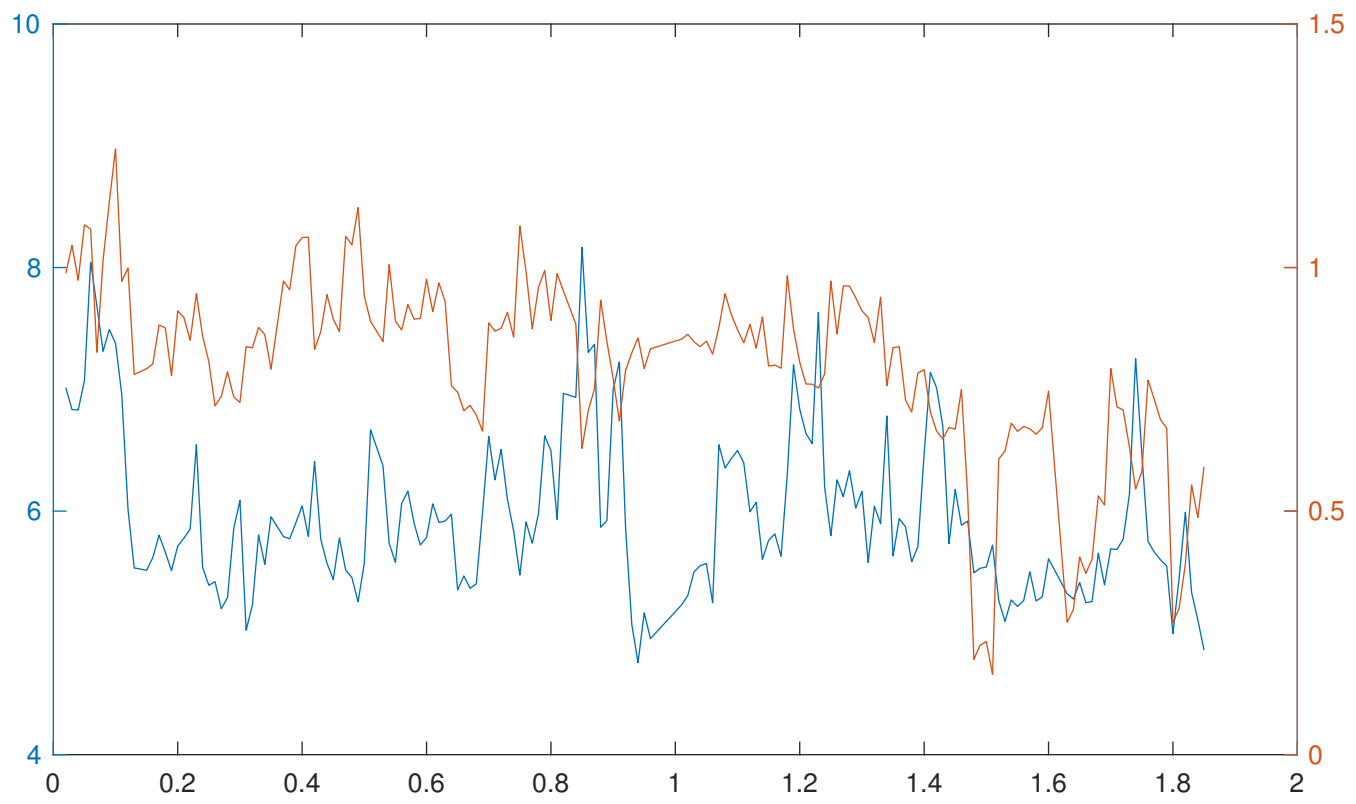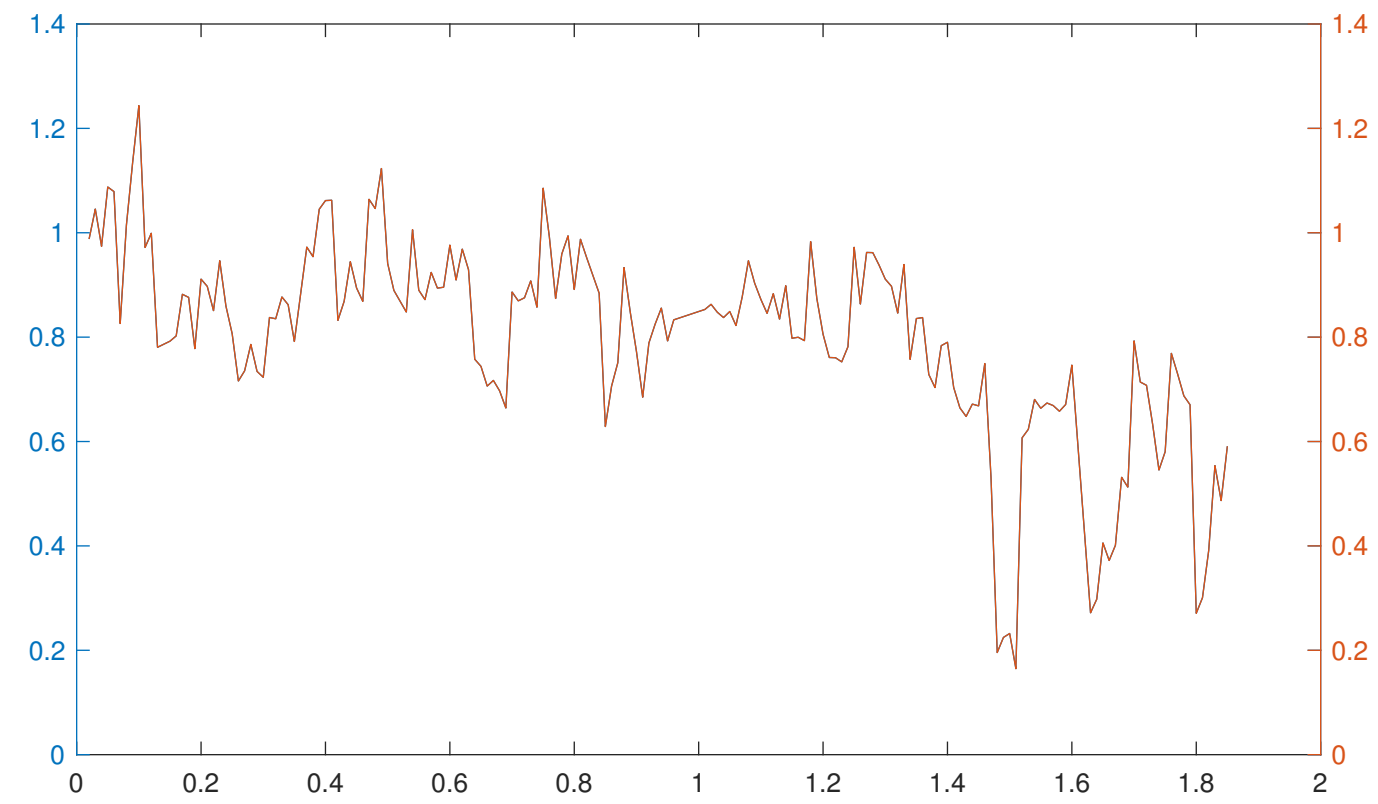

Supplement: S2 File — A zipped archive of all down-core plots and correlation tables used in this paper. (ZIP) [file pone.0199420.s006.zip › Downcore Plots and Correlation Tables/callao/figures/W7706-40.depth.curves.pdf]

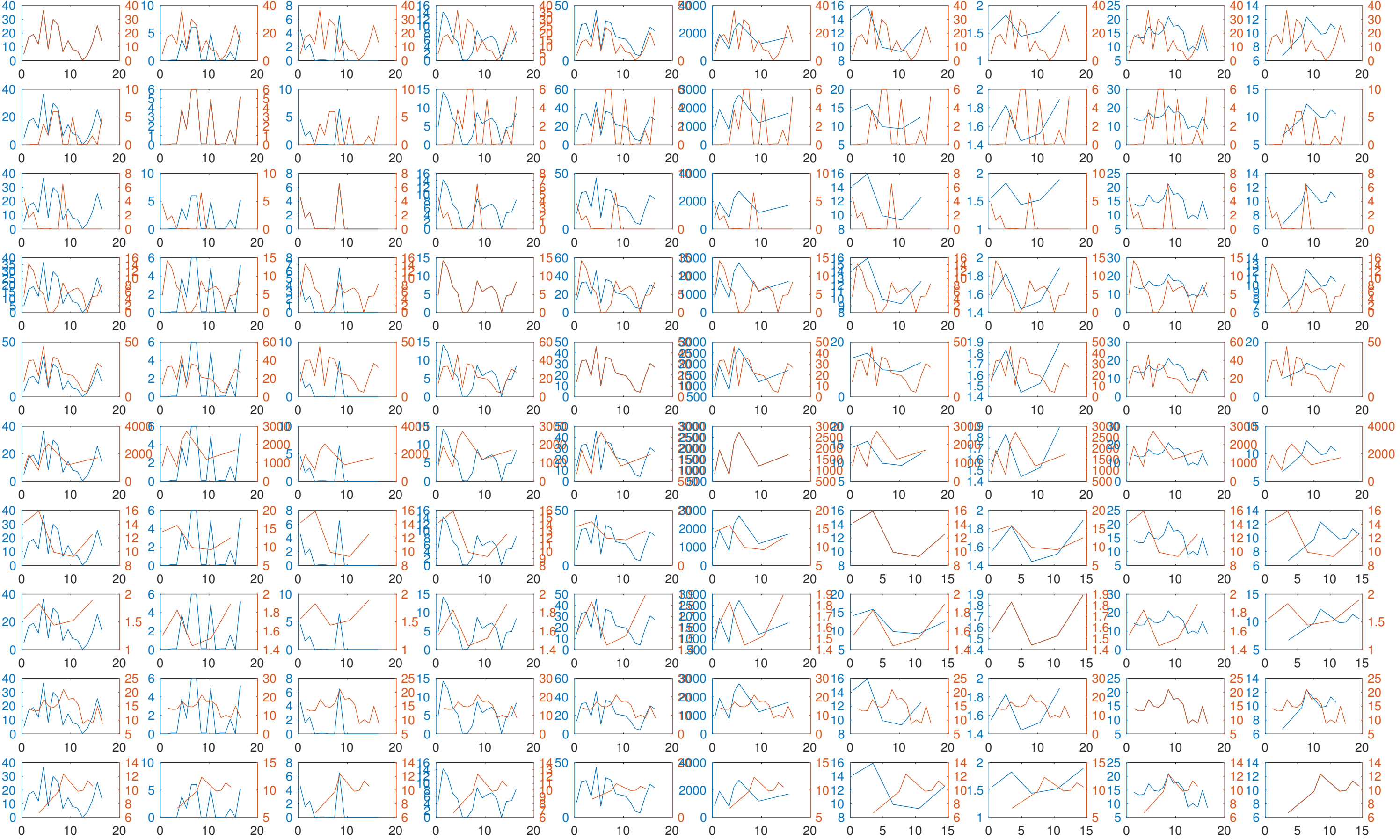

Supplement: S2 File — A zipped archive of all down-core plots and correlation tables used in this paper. (ZIP) [file pone.0199420.s006.zip › Downcore Plots and Correlation Tables/callao/figures/C0329.depth.curves.pdf]

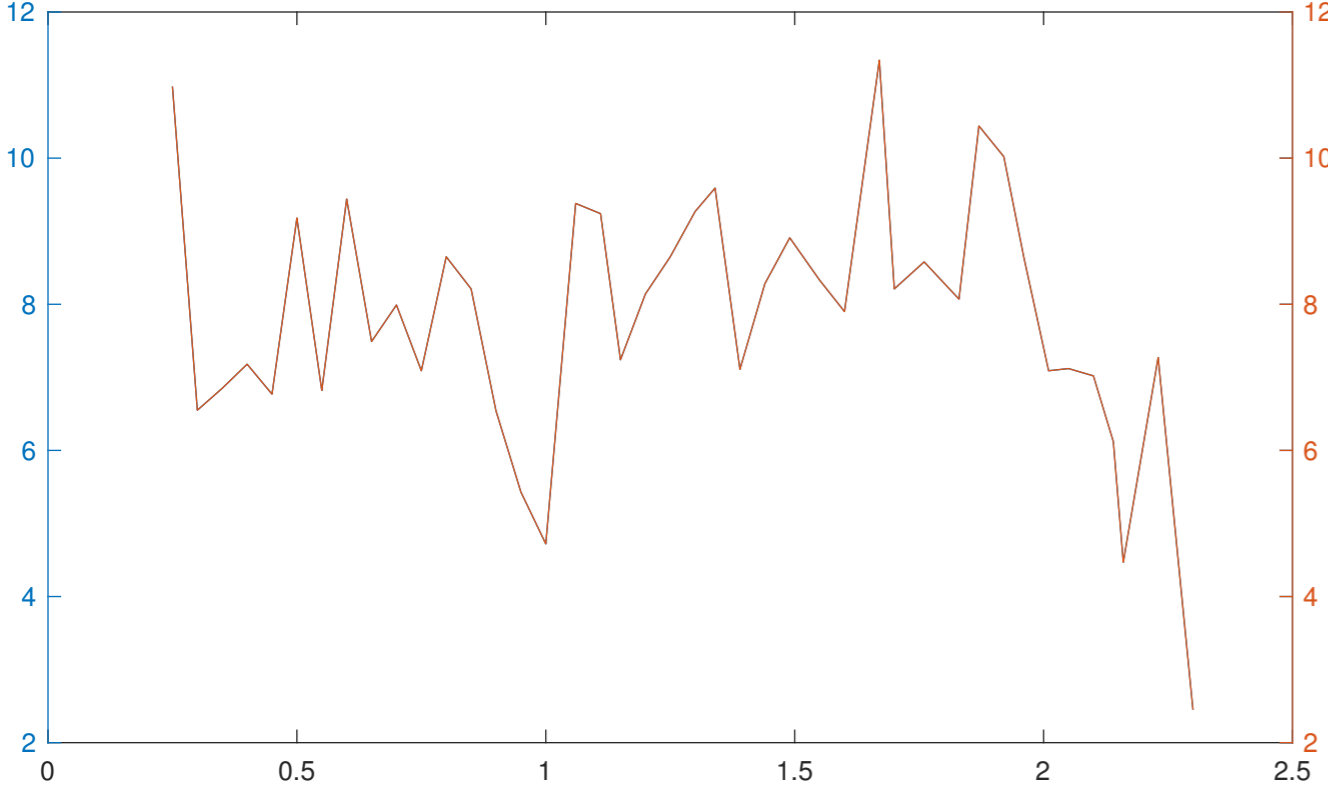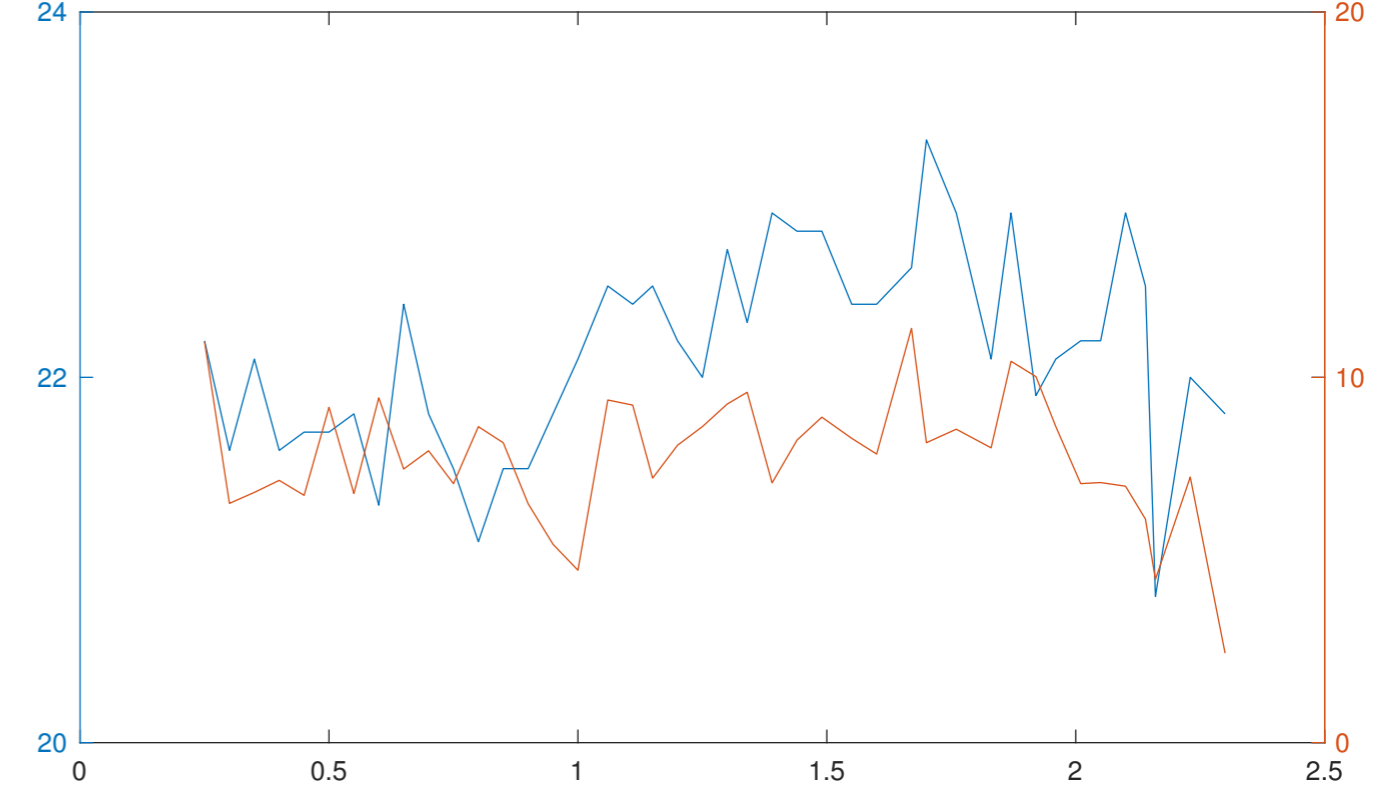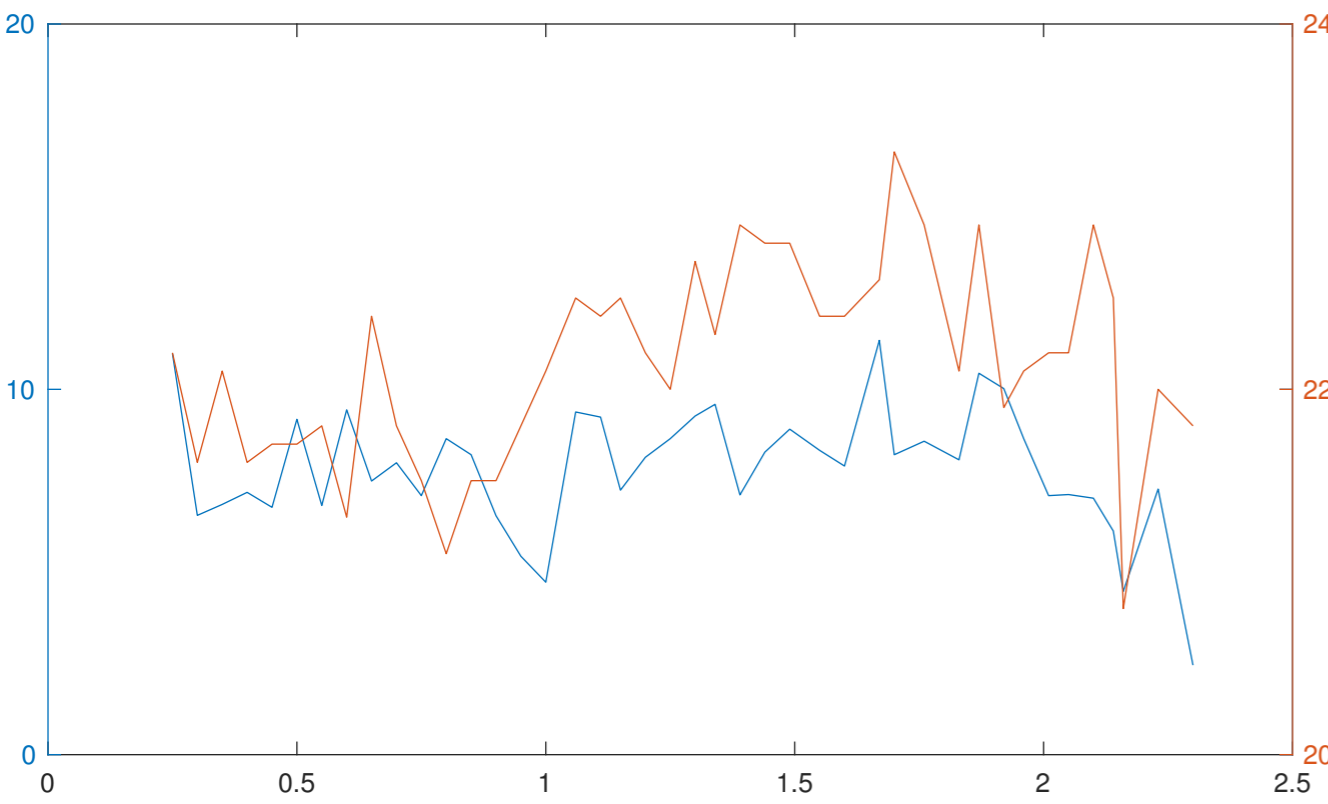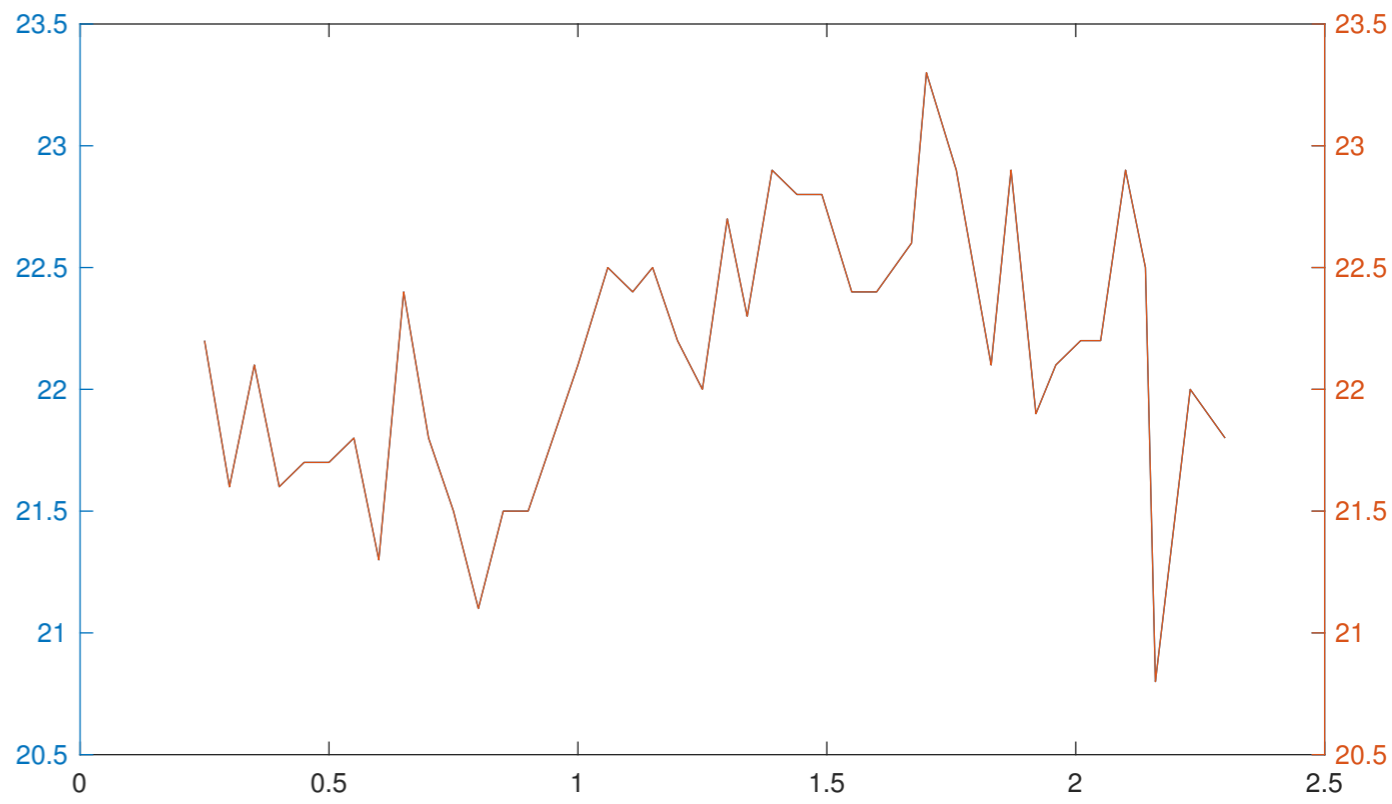

Supplement: S2 File — A zipped archive of all down-core plots and correlation tables used in this paper. (ZIP) [file pone.0199420.s006.zip › Downcore Plots and Correlation Tables/callao/figures/SO78-173-4.depth.curves.pdf]

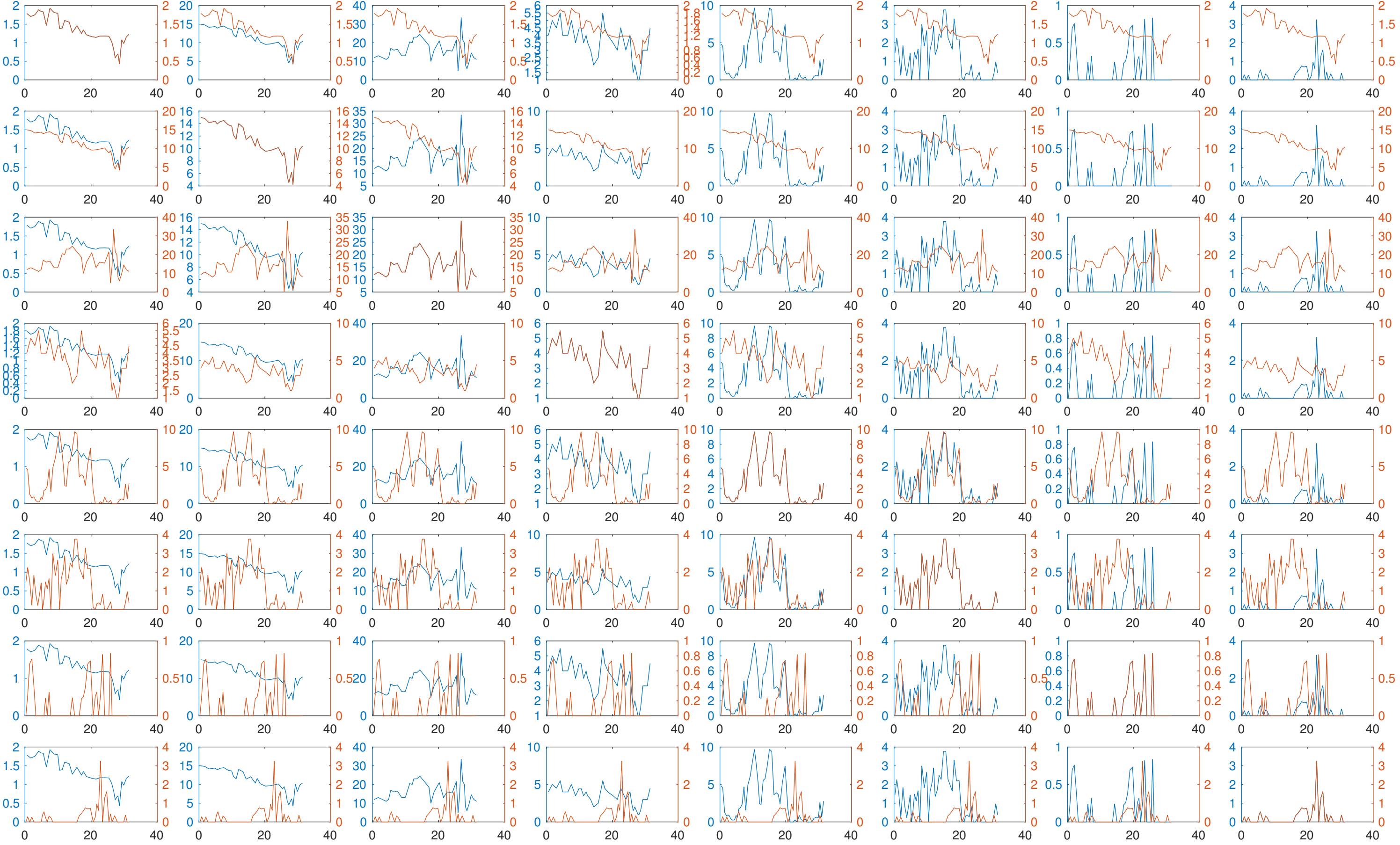

Supplement: S2 File — A zipped archive of all down-core plots and correlation tables used in this paper. (ZIP) [file pone.0199420.s006.zip › Downcore Plots and Correlation Tables/callao/figures/B0405-13.depth.curves.pdf]

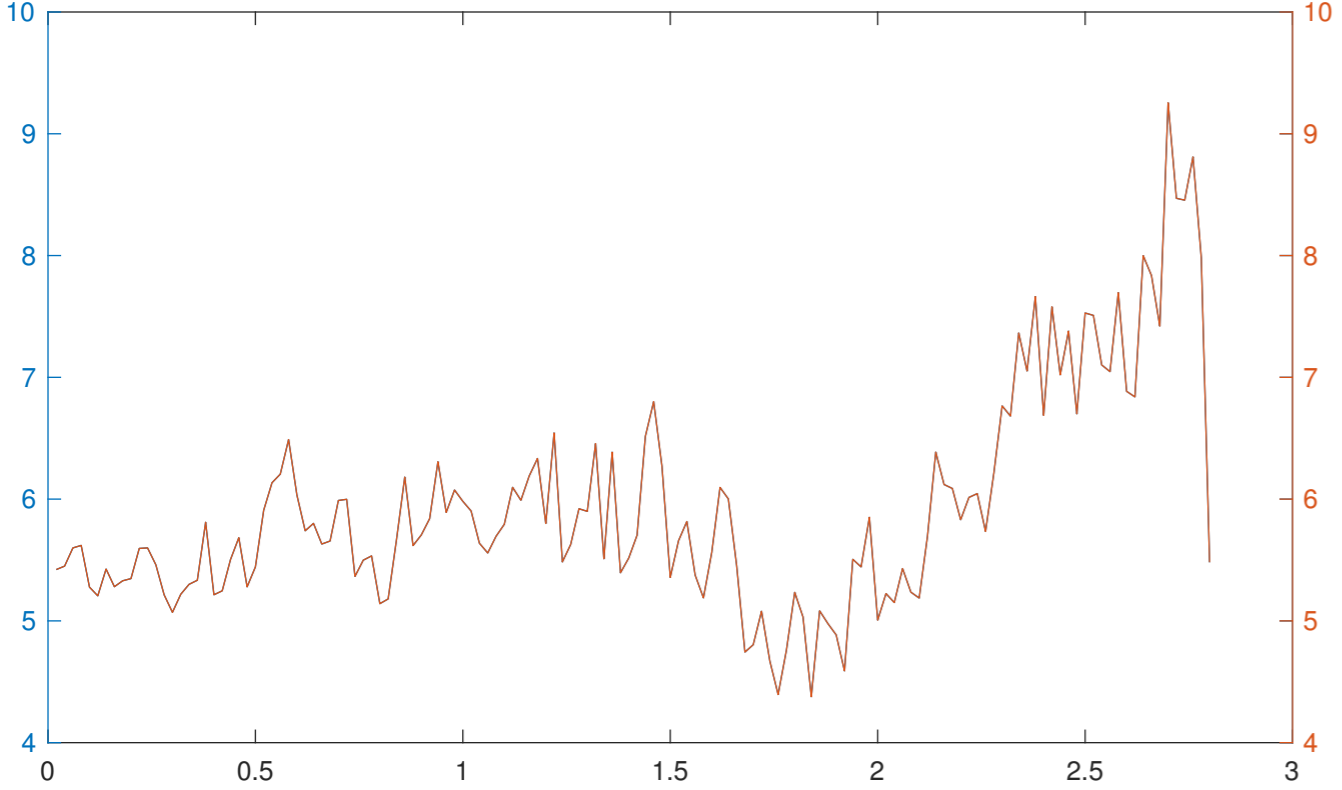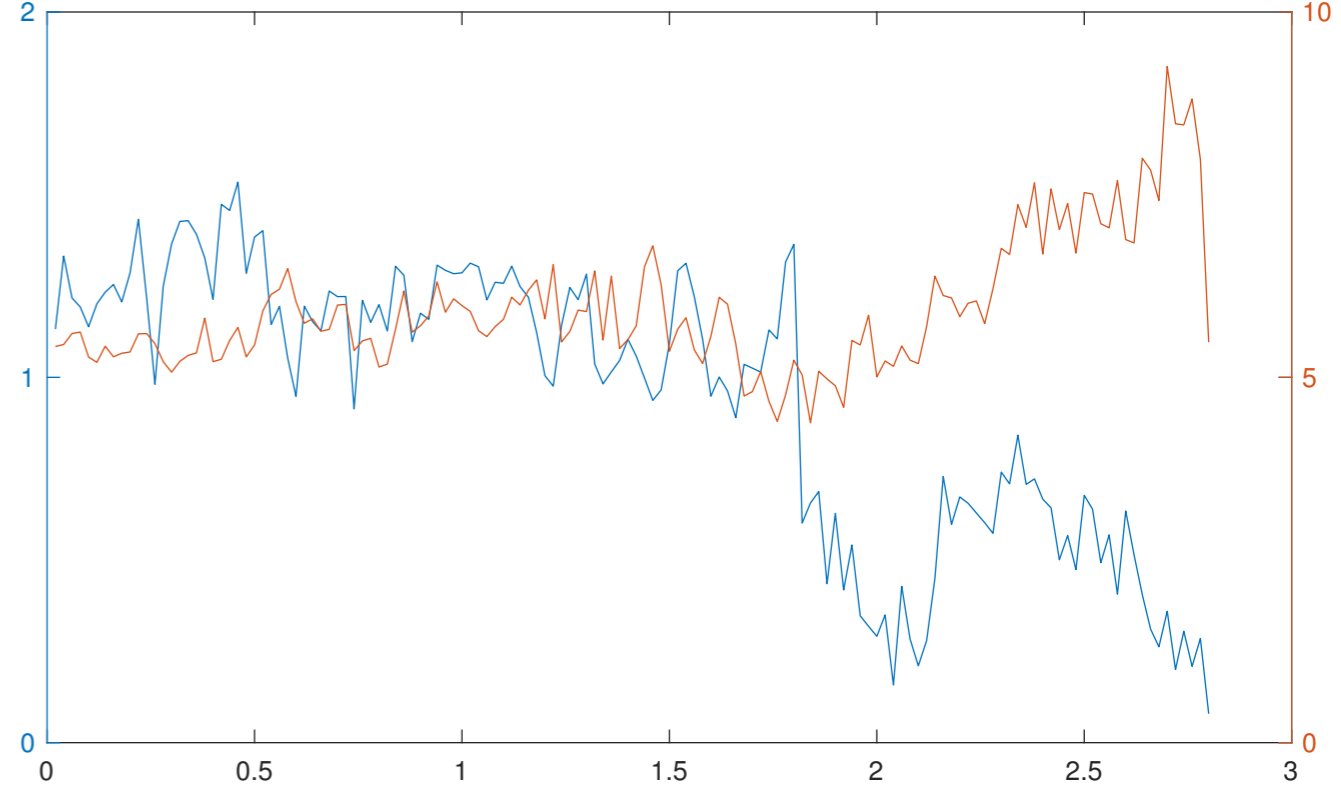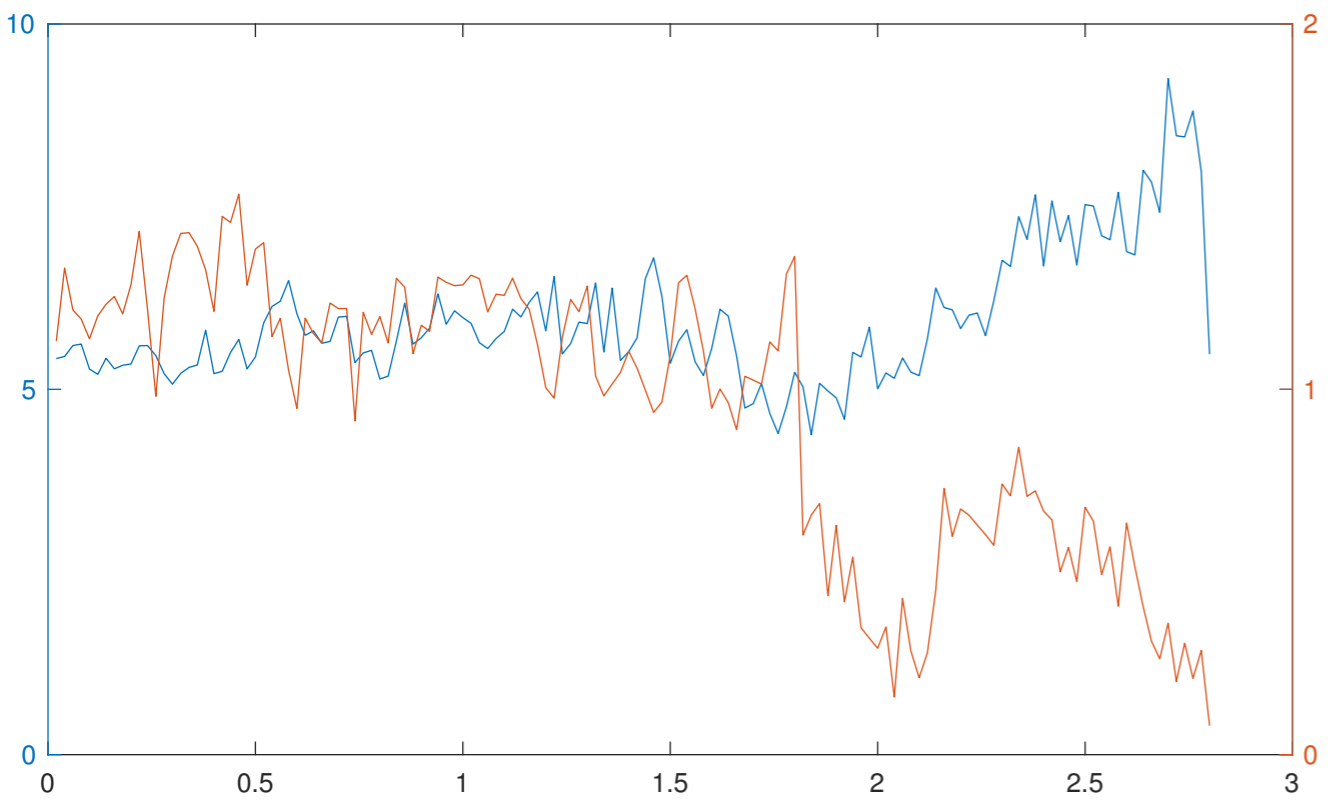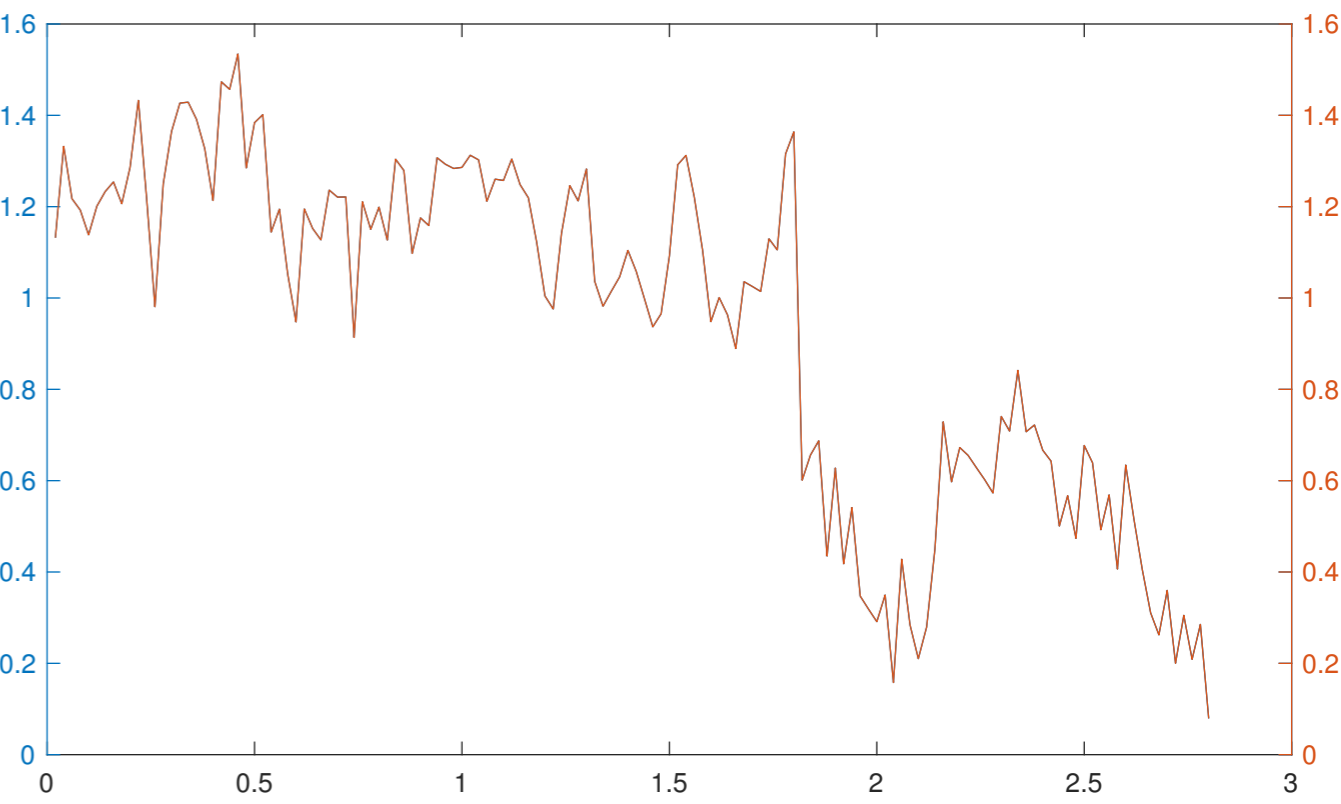

Supplement: S2 File — A zipped archive of all down-core plots and correlation tables used in this paper. (ZIP) [file pone.0199420.s006.zip › Downcore Plots and Correlation Tables/callao/figures/ODP1228.depth.curves.pdf]
